# A Modular Synthetic Approach to Isosteric Sulfonic Acid Analogues of the Anticoagulant Pentasaccharide Idraparinux

**DOI:** 10.3390/molecules21111497

**Published:** 2016-11-11

**Authors:** Erika Mező, Dániel Eszenyi, Eszter Varga, Mihály Herczeg, Anikó Borbás

**Affiliations:** Department of Pharmaceutical Chemistry, University of Debrecen, Egyetem tér 1, H-4032 Debrecen, Hungary; mezzo.erika@science.unideb.hu (E.M.); eszenyi.daniel@science.unideb.hu (D.E.); esztervargaa@gmail.com (E.V.); herczeg.mihaly@science.unideb.hu (M.H.)

**Keywords:** heparin, carbohydrates, glycosylation, sulfonic acid, uronic acid

## Abstract

Heparin-based anticoagulants are drugs of choice in the therapy and prophylaxis of thromboembolic diseases. Idraparinux is a synthetic anticoagulant pentasaccharide based on the heparin antithrombin-binding domain. In the frame of our ongoing research aimed at the synthesis of sulfonic acid-containing heparinoid anticoagulants, we elaborated a modular pathway to obtain a series of idraparinux-analogue pentasaccharides bearing one or two primary sulfonic acid moieties. Five protected pentasaccharides with different *C*-sulfonation patterns were prepared by two subsequent glycosylation reactions, respectively, using two monosaccharide and four disaccharide building blocks. Transformation of the protected derivatives into the fully *O*-sulfated, *O*-methylated sulfonic acid end-products was also studied.

## 1. Introduction

Venous and arterial thromboembolic disorders, including pulmonary embolism and deep vein thrombosis represent a serious medical and socioeconomic problem worldwide. Untreated thromboembolism leads to cardiac or cerebral infarction or, in more severe cases, to death. Anticoagulants are used in the prevention and treatment of venous thrombosis and in the prevention of systemic embolism [1,2,3,4]. The sulfated polysaccharide heparin and its fractionated derivatives have successfully been used in anticoagulant therapy and thromboprophylaxis since the late 1930s until today. Heparin derivatives indirectly inhibit the coagulation enzymes thrombin or factor Xa through activation of the serine protease inhibitor antithrombin, which is an endogenous regulatory protein in the coagulation cascade [5]. Despite their effectiveness in therapy, heparin polysaccharides may incur side effects including inflammation, bleeding or heparin induced thrombocytopenia (HIT) due to its highly polyanionic and heterogeneous nature [6].

After the antithrombin-binding pentasaccharide domain of heparin (**1**), termed **DEFGH**, was identified, its closely related synthetic analogue, fondaparinux **2**, has been developed into a novel antithrombotic under the name Arixtra [7,8]. This pentasaccharide selectively inhibits factor Xa and minimizes the bleeding risk and many other unfavorable factors in anticoagulant therapy. Further research efforts led to the development of the non-glycosaminoglycan derivative, idraparinux **3** [9], possessing a simplified structure and an increased anticoagulant activity compared to Arixtra.

The interaction between heparin and antithrombin are primarily mediated by negatively charged groups of heparin and the positively charged lysine and arginine residues from the protein. Structure-activity relationship (SAR) studies of synthetic analogues of heparin pentasaccharides revealed that the type of negative charge is crucial; the carboxylate groups cannot be exchanged for sulfate esters, and sulfate moieties cannot be exchanged for phosphate groups without destroying the anticoagulant activity [1,3].

**Figure 1:**
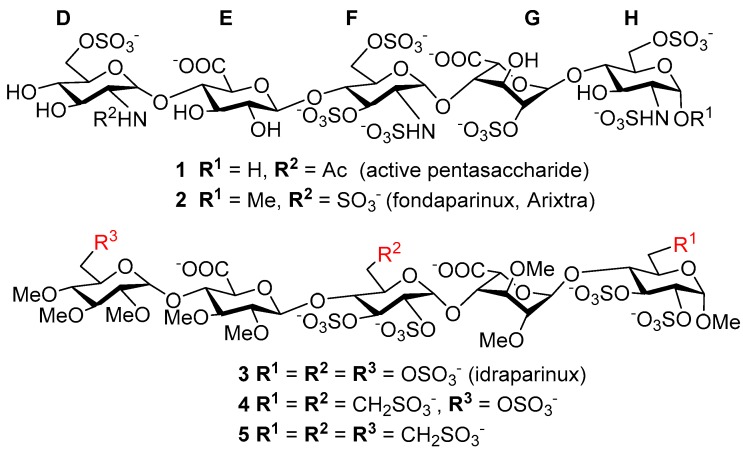
The antithrombin-binding pentasaccharide domain of heparin (**1**) and structures and factor Xa inhibitory activities of the synthetic analogues **2**–**5**.

With the aim of developing a novel class of heparinoid anticoagulants, our group has started a programme to study whether the sulfate ester groups of the active pentasaccharide can be exchanged with sulfonic acid moieties without detriment to the antithrombin-binding ability [10,11,12,13,14,15,16,17,18,19]. Two pentasaccharide sulfonic acids (**4** and **5**) and the reference compound idraparinux **3** have been prepared until now. An in vitro coagulation study of **2**–**5** clearly demonstrated that the position and/or number of the sulfonic acid moieties have a substantial impact on the anticoagulant activity (Figure 1). While the disulfonate analogue **4** displayed higher activity than the reference compounds **2** and **3**, the introduction of a third sulfonic-acid moiety to the terminal sugar unit of compound **5** resulted in a dramatic decrease in anti-Xa activity [14]. The difference in the biological activity of the disulfonic and trisulfonic acids was attributed to the different conformation of their l-iduronic-acid residues.

**Figure 2:**
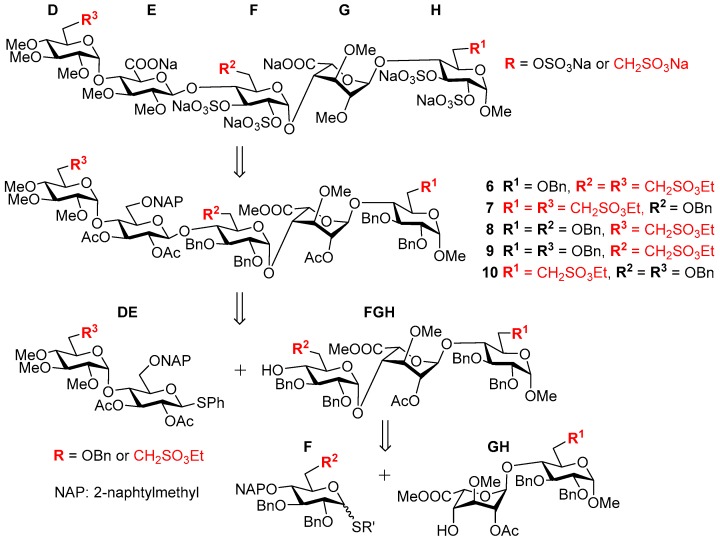
Retrosynthetic analysis for modular [2 + 3] block syntheses of the targeted pentasaccharide sulfonic acids **6**–**10**.

These results prompted us to prepare a series of heparinoid pentasaccharides by systematic replacement of the sulfate esters with a sodium sulfonatomethyl moiety for further structure-activity relationship studies. To get an easy access to all possible sulfonic acid isosters of idraparinux bearing the sulfonic acid moieties at primary positions, we elaborated a modular synthetic pathway based on the retrosynthetic analysis of the targeted pentasaccharides (Figure 2). According to this modular approach, the synthesis of all planned pentasaccharide sulfonic acids could be accomplished by using two **DE** disaccharide donors, two **F** building blocks and two **GH** disaccharide acceptors. Multigram-scale syntheses of the 6-deoxy-6-sulfonatomethyl-containing **F**, **DE** and **GH** building blocks have been published recently [17]. Herein, we present the synthesis of the **FGH** acceptors and assembly of five protected pentasaccharide sulfonic acids via [2 + 3] block syntheses. Our study to convert the protected mono- and disulfonic acid derivatives into the corresponding fully *O*-sulfated and *O*-methylated end-products via two different reaction sequences is also described.

## 2. Results and Discussion

### 2.1. Synthesis of the Protected Pentasaccharides

**Scheme 1:**
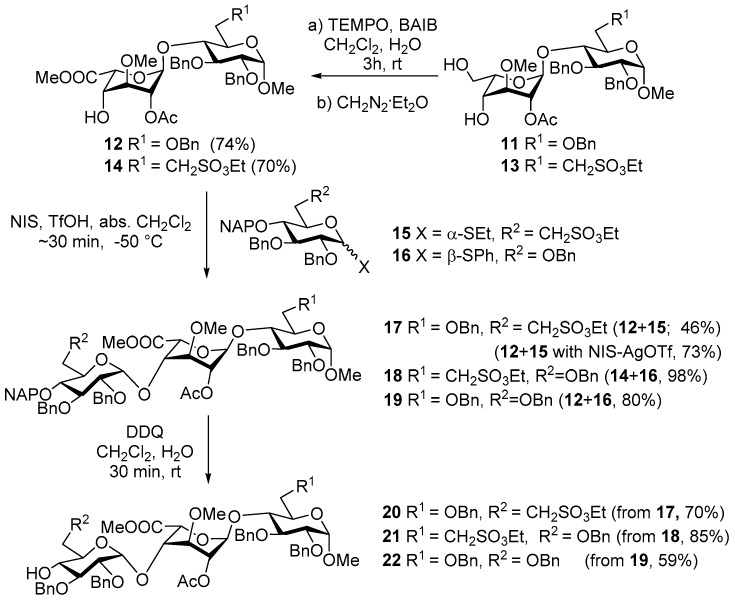
Preparation of the FGH building blocks.

The synthetic route to the **FGH** trisaccharide acceptors **20**–**22** is shown in Scheme 1. First, the l-idose-containing diol **11** [18] was converted to iduronide acceptor **12** by selective oxidation using (2,2,6,6-tetramethylpiperidin-1-yl)oxyl (TEMPO) as the oxidant and [bis(acetoxy)iodo]benzene (BAIB) as the co-oxidant [20,21]. The corresponding sulfonic acid isoster **14** [17] was prepared from **13** [17] in an analogous way. Next, **GH** disaccharide acceptors **12** and **14** were reacted with **F** monosaccharide donors **15** [16] and **16** [18], respectively, in the presence of *N*-iodosuccinimide (NIS) and trifluoromethanesulfonic acid (TfOH). This promoter system proved to be highly efficient for stereoselective condensation of the phenylthio-glucoside donor **16** with either of the acceptors, and the corresponding trisaccharides **18** and **19**, with the required α-interglycosidic linkage, were obtained in 98% and 80% yields, respectively. NIS-TfOH-promoted glycosylation of **12** with the sulfonatomethyl donor **15** also occurred with full α-selectivity affording trisaccharide **17** as the only product. However, the yield was moderate due to insufficient conversion of the acceptor. Fortunately, by changing the promotors to NIS-AgOTf, the conversion could significantly be increased, and the yield of **17** reached 73%. Liberation of the 4-OH group of the terminal glucose unit of the fully protected trisaccharides **17**–**19** was accomplished by oxidative removal of the (2-naphthyl)methyl (NAP) group using 2,3-dichloro-5,6-dicyano-1,4-benzoquinone (DDQ) as the reagent [22,23], furnishing the FGH acceptors **20**–**22** in good to excellent yields.

**Scheme 2:**
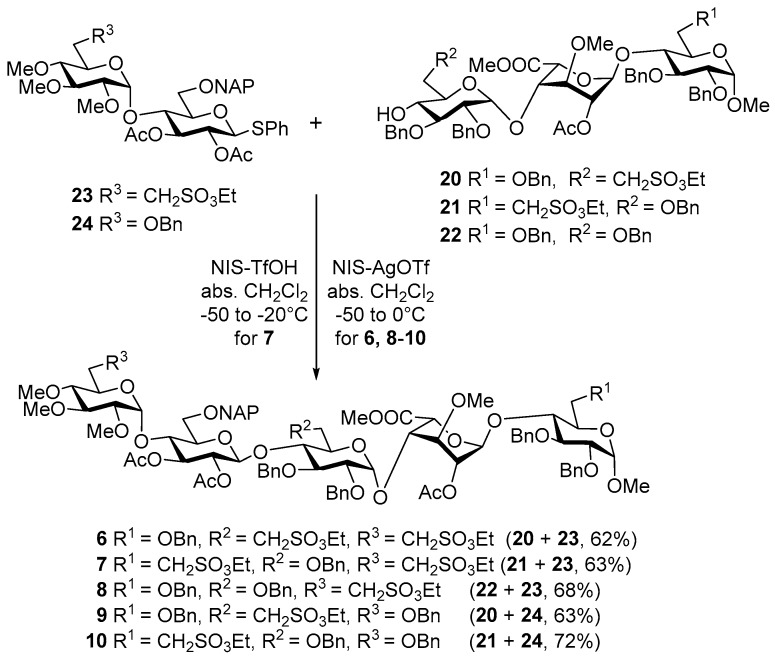
Synthesis of the protected pentasaccharide derivatives.

To avoid inefficient glycosylations with glucuronic acid donors of inherent low reactivity, which were observed in earlier syntheses [9,15,24], donors **23** [17] and **24** [13] containing a non-oxidized precursor of the glucuronide unit were used for [2 + 3] block syntheses of the targeted pentasaccharides (Scheme 2). Condensation reactions of the trisaccharide acceptors **20**–**22** with the disaccharide donors **23** and **24** were carried out upon NIS-AgOTf or NIS-TfOH activation, respectively. All reactions took place in a stereoselective way providing the protected pentasaccharides **6**–**10**, with the required β-linkage between units **E** and **F**, in good yields.

### 2.2. Transformation of the Protected Pentasaccharides into the End-Products

**Scheme 3:**
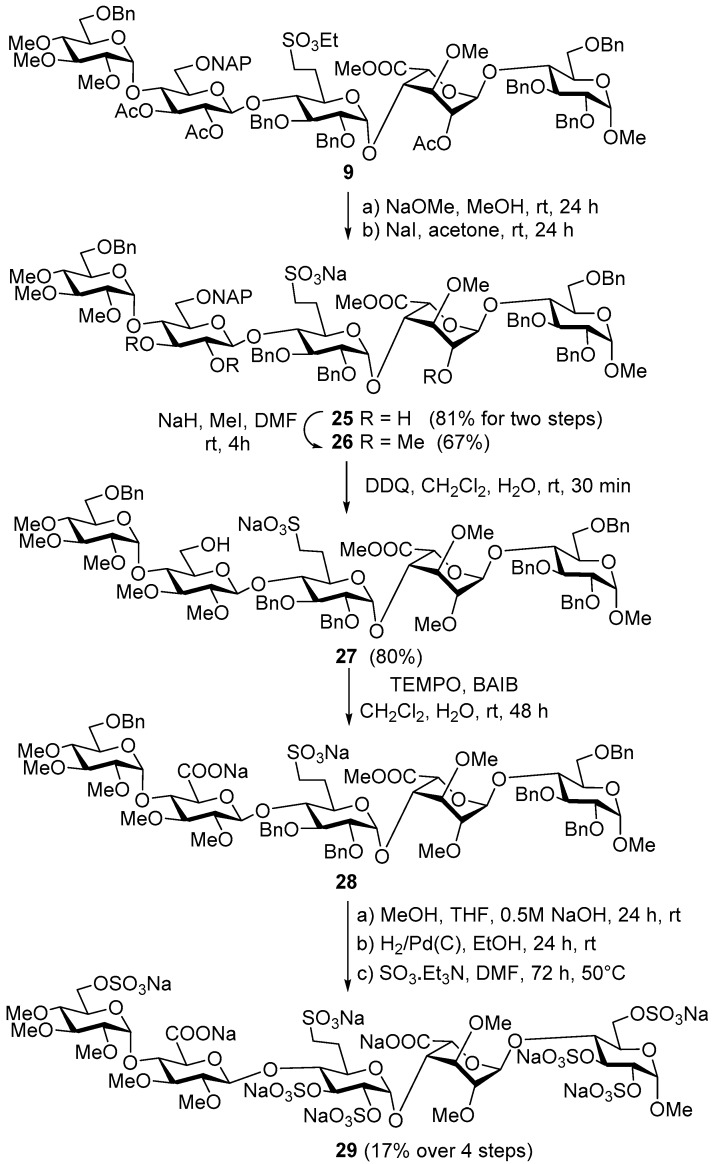
Transformation of **9** into final product **29**, a novel sulfonic acid analogue of idraparinux.

Transformation of compounds **6**–**10** into the fully *O*-methylated and *O*-sulfated mono- and disulfonic acid end-products requires eight further synthetic steps including acetyl-, NAP- and benzyl-deprotections, formation of the glucuronic acid unit, liberation of carboxylic and sulfonic esters and the introduction of methyl ether and sulfate ester functions. We envisioned a reaction sequence in which the introduction of methyl ethers precedes the oxidative formation of the glucuronide residue **E**. To study the efficacy of this procedure, compound **9** was subjected to Zemplén deacetylation to liberate the hydroxyls to be methylated (Scheme 3). Upon deacetylation with NaOMe, nucleophilic cleavage of the sulfonic-acid ester of unit **F** also occurred in some extent. Hence, the obtaining mixture of the sulfonate ester and sodium sulfonate derivatives was unified by treating with sodium iodide in acetone to give sulfonic acid salt **25** in an 81% yield over two steps. Introduction of the methyl ethers to the liberated hydroxyls was accomplished by alkylation using methyl iodide and sodium hydride to afford the desired **26** in a 67% yield. Subsequently, the 6-position of the penultimate glucose unit was unmasked by oxidative de-*O*-(2-napthyl)methylation to produce **27** in 80%. TEMPO-BAIB mediated oxidation of **27** proceeded slowly and required large amounts of the co-oxidant BAIB to eventually produce, after 48 h, the glucuronide derivative **28**, along with its partially debenzylated derivatives. Compound **28** could not be separated from the by-products, thus, this mixture was subjected to the remaining transformation, including basic hydrolysis of the iduronic ester, catalytic hydrogenolysis and *O*-sulfation to furnish **29**, a monosulfonic acid analogue of idraparinux.

**Scheme 4:**
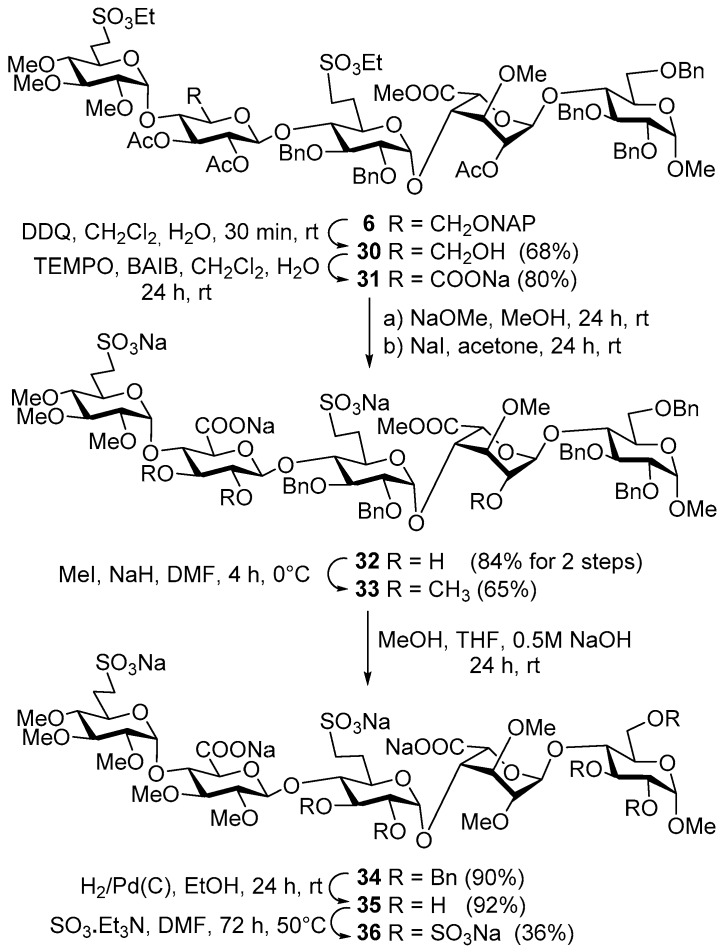
Transformation of **6** into the corresponding pentasaccharide disulfonic acid final product.

For conversion of **6** into the final sulfated analogue, another reaction sequence, starting with the formation of the glucuronide residue, was used. The NAP protecting group of unit **E** was cleaved with DDQ to afford **30**. Oxidation of the liberated 6-OH by TEMPO and BAIB proceeded smoothly to provide the required glucuronic acid-containing pentasaccharide **31** in 80%. Zemplén deacetylation followed by deprotection of the sulfonic acid esters by nucleophilic displacement with sodium iodide gave the trisodium salt **32**. Methylation of the free hydroxyl groups by using methyl iodide and sodium hydride afforded the desired **33**, which possessed all of the required methyl ethers. Deprotection of the carboxylic-ester group of **33** by saponification gave tetrasodium salt **34**, de-*O*-benzylation of which, by catalytic hydrogenolysis, furnished compound **35** in a high yield. *O*-Sulfation of the pentaol by using SO_3_·Et_3_N was surprisingly sluggish and the use of a high excess reagent and a prolonged reaction time were needed for completion of the sulfate ester formation. Finally, the reaction gave, after treatment with Dowex Na^+^ ion-exchange resin, compound **36** as a new isosteric disulfonic acid analogue of idraparinux (Scheme 4).

The ^1^H-NMR, and ^13^C-NMR spectra of the new compounds are reported in the Supplementary data.

## 3. Materials and Methods

### 3.1. General Information

Optical rotations were measured at room temperature with a Perkin-Elmer 241 automatic polarimeter. Thin layer chromatography (TLC) was performed on Kieselgel 60 F254 (Merck) with detection by immersing into 5% ethanolic sulfuric acid solution followed by heating. Column chromatography was performed on Silica gel 60 (Merck 0.063–0.200 mm). Organic solutions were dried over MgSO_4_, and concentrated in a vacuum. The ^1^H-NMR (360 and 400 MHz) and ^13^C-NMR (90.54 and 100.28 MHz) spectra were recorded with Bruker DRX-360 and DRX-400 spectrometers at 25 °C. Chemical shifts are referenced to Me_4_Si or 4,4-dimethyl-4-silapentane-1-sulfonic acid (DSS) (0.00 ppm for ^1^H) and to the solvent signals (CDCl_3_: 77.00 ppm for ^13^C). The ^1^H- and ^13^C-NMR assignments have been established from ^1^D-NMR spectra and for compounds **6**, **7**, **8**, **9**, **10**, **20** and **21** the proton-signal assignments were supported by analysis of two-dimensional ^1^H–^1^H correlation spectra (COSY), as well as the carbon-signal assignments by two-dimensional ^13^C–^1^H correlation maps (HSQC). Infrared (IR) spectra were recorded on a Perkin–Elmer 16 PC FTIR (Program counter Fourier transform infrared) spectrometer. Matrix-assisted laser desorption/ionization-time-of-flight mass spectrometric (MALDI-TOF MS analyses of the compounds were carried out in the positive reflectron mode using a BIFLEX III mass 13 spectrometer (Bruker, Rheinstetten, Germany) equipped with delayed-ion extraction. The matrix solution was a saturated 2,4,6-trihydroxy-acetophenone (THAP) solution in MeCN. Elemental analyses (C, H, S) were performed using an Elementar Vario MicroCube instrument. 

### 3.2. General Method A for TEMPO-BAIB Oxidation *(**12***, ***28***, ***31**)*

To a vigorously stirred solution of the appropriate alcohol (1 mmol) in CH_2_Cl_2_ (3.5 mL) and H_2_O (1.5 mL), TEMPO (0.2 mmol) and BAIB (2 mmol) were added and the reaction mixture was stirred until TLC showed complete conversion of the starting material. The reaction time was 45 min for **12**, 24 h for **31** and 48 h for **28**. The reaction mixture was quenched by the addition of 10% aq Na_2_S_2_O_3_ solution (20 mL). The mixture was then extracted twice with EtOAc (10 mL), and the combined organic layers were dried, and concentrated.

### 3.3. General Method B for Glycosylation Reaction Using NIS-TfOH Promoter System *(**7***, ***17***, ***18***, ***19**)*

To a solution of donor (1.5 mmol) and acceptor (1 mmol) in dry CH_2_Cl_2_ (20 mL), 4 Å molecular sieves were added. The stirred mixture was cooled to −60 °C (**17**, **18**, **19**) and −50 °C (**7**) under argon. After 30 min at this temperature, NIS (2.25 mmol) and TfOH (0.045 mmol) dissolved in THF (155 μL) were added. The temperature was increased to −50 °C (**17**, **18**, **19**) or −20 °C (**7**). The reaction mixture was quenched with Et_3_N (50 μL), diluted with CH_2_Cl_2_ (100 mL) and the reaction mixture was extracted with saturated Na_2_S_2_O_3_ solution (20 mL), saturated NaHCO_3_ solution (20 mL) and with distilled water (20 mL). The organic phase was dried and concentrated.

### 3.4. General Method C for Glycosylation Using NIS-AgOTf Promoter System *(**6***, ***8***, ***9***, ***10***, ***17**)*

To a solution of acceptor (1 mmol) and donor (1.5 mmol) in dry CH_2_Cl_2_ (20 mL), 4 Å molecular sieves were added. The stirred mixture was cooled to between −60 °C and −35 °C under argon. After 30 min at this temperature, 2.25 mmol NIS dissolved in THF (1 mL) and 0.36 mmol AgOTf dissolved in toluene (1 mL) were added. After 1–4 h stirring at −50 °C (**17**), −20 °C (**9**), −15 °C (**6**), −10 °C (**10**) or 0 °C (**8**) Et_3_N (50 μL) was added. The reaction mixture was diluted with CH_2_Cl_2_ (100 mL) and filtered through a pad of Celite. The filtrate was concentrated.

### 3.5. General Method D for Removal of the (2-Naphthyl)methyl Ether Group *(**20***, ***21***, ***22***, ***27***, ***30**)*

To a vigorously stirred solution of starting material (1 mmol) in CH_2_Cl_2_: H_2_O (9:1, 10 mL) DDQ (1.5 mmol) was added. The reaction mixture was stirred at room temperature for 30–40 min, diluted with CH_2_Cl_2_ (30 mL), washed successively with satd aq NaHCO_3_ solution (15 mL) and H_2_O (15 mL). The organic phase was dried and concentrated.

### 3.6. Product Characterization

*Methyl [methyl (2-O-acetyl-3-O-methyl-α-l-idopyranosyl)uronate](1→4)-2,3,4-tri-O-benzyl-α-d-glucopyranoside* (**12**). The starting material **11** [18] (3.20 g, 4.7 mmol) was oxidized according to general method A. The crude uronic acid was dissolved in THF (4.5 mL) and treated with ethereal diazomethane at 0 °C. After complete disappearance of the uronic acid, the mixture was concentrated. The crude product was purified by column chromatography to give **12** (2.47 g, 74%) as a colourless syrup; *R*_f_ = 0.36 (7:3 *n*-hexane/acetone); [α]_d_ −2.67 (*c* 1.00, CHCl_3_); IR ν_max_ (KBr): 3480, 3474, 3031, 2936, 2902, 1744, 1633, 1496, 1454, 1372, 1225, 1167, 1103, 1045, 911, 890, 854, 740, 700, 605 cm^−1^; ^1^H-NMR (360 MHz, CDCl_3_) δ 7.37–7.20 (m, 15H, arom.), 5.08 (s, 1H), 4.98 (d, *J* = 11.4 Hz, 2H), 4.87 (s, 1H), 4.81 (d, *J* = 11.4 Hz, 1H, PhC*H*_2_), 4.67 (d, *J* = 12.0 Hz, 1H, PhC*H*_2_), 4.56–4.49 (m, 4H), 3.99–3.80 (m, 3H), 3.78–3.70 (m, 2H), 3.67–3.62 (m, 1H), 3.57 (dd, *J* = 9.4, 3.6 Hz, 1H), 3.50–3.44 (m, 1H), 3.47, 3.40, 3.34 (3s, 9H, 3 × C*H*_3_), 2.68 (d, *J* = 11.8 Hz, 1H, OH), 2.01 (s, 3H, COC*H*_3_) ppm; ^13^C-NMR (91 MHz, CDCl_3_) δ 169.7, 169.2 (COOCH_3_, COCH_3_), 139.0, 138.0, 138.0 (3C, *C*_q_ arom.), 128.4, 128.4, 128.2, 128.1, 127.9, 127.6, 127.6, 127.2, 127.0 (15C, arom.), 98.0, 97.6 (C-1-H, C-1-G), 80.3, 79.7, 76.8, 74.7, 70.1, 68.0, 67.2, 67.1 (C-2-G, C-2-H, C-3-G, C-3-H, C 4 G, C-4-H, C-5-G, C-5-H), 74.8, 73.4, 73.3 (3 × Ph*C*H_2_), 68.5 (C-6-H), 58.1 (O*C*H_3_, C-3-G), 55.2 (O*C*H_3_, C-1-H), 51.9 (COO*C*H_3_), 21.00 (CO*C*H_3_) ppm; MALDI-TOF MS: *m*/*z* 733.16 [M + Na]^+^ (Calcd. 733.28); Anal. Calcd. for C_38_H_46_O_13_ (710.29): C, 64.21; H, 6.52; O, 29.26. Found: C, 64.28; H, 6.55.

*Methyl [2,3-di-O-Benzyl-4-O-(2-naphthyl)methyl-6-deoxy-6-C-(ethylsulfonatomethyl)-α-d-glucopyranosyl]-(1→4)-[methyl (2-O-acetyl-3-O-methyl-α-l-idopyranosyl)uronate]-(1→4)-(2,3,6-tri-O-benzyl-α-d-glucopyranoside)* (**17**)

**I**. To a solution of acceptor **12** (2.80 g, 3.94 mmol) and donor **15** [16] (3.85 g, 5.91 mmol) in dry CH_2_Cl_2_ (20 mL), 4 Å molecular sieves (0.50 g) were added. The stirred mixture was cooled to −60 °C under argon and activated by method B. The reaction mixture was allowed to warm up to −50 °C in 1 h. The crude product was purified by column chromatography (7:3 *n*-hexane/EtOAc) to give **17** (2.35 g, 46%). Unreacted **12** (1.11 g, 28%) was recovered as a colourless syrup.

**II**. To a solution of acceptor **12** (1.11 g, 1.41 mmol) and donor **15** (1.37 g, 2.11 mmol) in dry CH_2_Cl_2_ (20 mL), 4 Å molecular sieves (0.50 g) were added. The stirred mixture was cooled to −60 °C under argon and activated by method **C**. The reaction mixture was allowed to warm up to −50 °C for 1 h. The crude product was purified by column chromatography (7:3 *n*-hexane/EtOAc) to give **17** (1.47 g, 73%) as a white foam; *R*_f_ = 0.34 (6:4 *n*-hexane/EtOAc); [α]_d_ +11.30 (*c* 0.81, CHCl_3_); IR ν_max_ (KBr): 3447, 3087, 3061, 3030, 2933, 1763, 1737, 1636, 1604, 1497, 1455, 1369, 1357, 1238, 1169, 1107, 1028, 1002, 917, 857 cm^−1^; ^1^H-NMR (360 MHz, CDCl_3_) δ 7.83–7.66 (m, 4H, arom.), 7.49–7.42 (m, 2H, arom.), 7.39–7.16 (m, 26H, arom.), 5.13 (s, 1H), 5.03 (d, *J* = 11.3 Hz, 1H, ArC*H*_2_), 4.93 (m, 2H), 4.85–4.72 (m, 7H), 4.66 (t, *J* = 12.6 Hz, 2H), 4.60–4.54 (m, 3H), 4.12 (q, *J* = 7.1 Hz, 2H, SO_3_C*H*_2_CH_3_), 3.96–3.61 (m, 9H), 3.54 (dd, *J* = 9.4, 3.5 Hz, 1H), 3.44–3.34 (m, 1H), 3.41, 3.36, 3.34 (3s, 9H, 3 × OC*H*_3_), 3.24–3.05 (m, 3H), 2.36–2.24 (m, 1H, H-7_a_), 2.00–1.83 (m, 4H, COC*H*_3_, H-7_b_), 1.25 (t, *J* = 7.1 Hz, 3H, SO_3_CH_2_C*H*_3_) ppm; ^13^C-NMR (91 MHz, CDCl_3_) δ 170.1, 169.4 (2 × *C*O), 139.1, 138.4, 138.1, 138.1, 138.1, 135.6, 133.3, 133.0 (8C, *C*_q_ arom.), 128.6, 128.5, 128.4, 128.4, 128.2, 128.2, 128.0, 127.9, 127.9, 127.7, 127.6, 127.3, 127.1, 126.5, 126.2, 126.0, 125.7 (32C, arom.), 98.7, 98.0, 97.7 (3 × C-1), 81.5, 81.2, 80.4, 80.2, 79.8, 76.5, 74.7, 74.7, 70.1, 69.5, 68.3, 67.7 (12C, skeleton carbons), 75.5, 75.1, 74.9, 73.6, 73.4, 73.4 (6 × Ar*C*H_2_), 68.5 (C-6-F), 66.2 (SO_3_*C*H_2_CH_3_), 58.3, 55.2, 51.8 (3 × O*C*H_3_), 46.5 (C-7-H), 25.9 (C-6-H), 21.1 (CO*C*H_3_), 15.1 (SO_3_CH_2_*C*H_3_) ppm; MALDI-TOF MS: *m*/*z* 1321.57 [M + Na]^+^ (Calcd. 1321.50); Anal. Calcd. for C_72_H_82_O_20_S (1298.51): C, 66.55; H, 6.36; O, 24.62; S, 2.47. Found: C, 66.62; H, 6.40; S, 2.45.

*Methyl [2,3,6-tri-O-benzyl-4-O-(2-naphthyl)methyl-α-d-glucopyranosyl]-(1→4)-[methyl (2-O-acetyl-3-O-methy-α-l-idopyranosyl)uronate]-(1→4)-2,3-di-O-benzyl-6-deoxy-6-C-(ethylsulfonatomethyl)-α-d-glucopyranoside* **18**. To a solution of acceptor (**14**) [17] (620 mg, 0.85 mmol) and donor **16** [17] (874 mg, 1.28 mmol) in dry CH_2_Cl_2_ (20 mL) 4 Å molecular sieves (0.50 g) were added. The stirred mixture was cooled to −60 °C under argon and activated by method **B**. The reaction mixture was allowed to warm up to −50 °C for 1 h. The crude product was purified by column chromatography (7:3 *n*-hexane/EtOAc) to give **18** (1.07 g, 98%) as a white foam; *R*_f_ = 0.63 (1:1 *n*-hexane/ EtOAc); [α]_d_ +6.62 (*c* 0.35, CHCl_3_); IR ν_max_ (KBr): 3446, 3087, 3061, 3030, 2931, 2869, 1739, 1636, 1604, 1497, 1455, 1362, 1236, 1165, 1107, 1045, 1028, 1004, 917, 857, 818 cm^−1^; ^1^H-NMR (360 MHz, CDCl_3_) δ 7.85–7.67 (m, 3H, arom.), 7.55 (s, 1H, arom), 7.49–7.41 (m, 2H, arom.), 7.38–7.14 (m, 26H, arom.), 5.25 (s, 1H), 4.99–4.35 (m, 16H), 4.27 (q, *J* = 7.1 Hz, 2H, SO_3_C*H*_2_CH_3_), 3.97–3.63 (m, 7H), 3.62–3.24 (m, 4H), 3.47, 3.40, 3.32 (3 × OC*H*_3_), 3.16–3.04 (m, 1H), 2.44–2.32 (m, 1H, H-7_a_), 2.03 (s, 3H, COC*H*_3_), 2.01–1.85 (m, 1H, H-7_b_), 1.37 (t, 3H, SO_3_CH_2_C*H*_3_) ppm; ^13^C-NMR (91 MHz, CDCl_3_) δ 170.2, 169.6 (2 × *C*O), 139.0, 138.7, 138.3, 138.1, 138.0, 136.3, 133.3, 132.9 (8C, *C*_q_ arom.), 128.5, 128.4, 128.2, 128.1, 128.0, 127.9, 127.7, 127.2, 126.1, 125.9, 125.8, 125.6 (32C, arom.), 99.6, 98.0, 98.0 (3 × C-1), 81.8, 80.3, 80.0, 79.5, 78.6, 77.0, 75.1, 71.4, 69.2, 68.4, 68.1, 57.9 (12C, skeleton carbons), 75.5, 75.2, 74.7, 73.5, 73.5, 73.4 (6 × Ar*C*H_2_), 68.1 (C-6-H), 66.2 (SO_3_*C*H_2_CH_3_), 58.7, 55.6, 51.9 (3 × O*C*H_3_), 46.7 (C-7-F), 26.0 (C-6-F), 21.1 (CO*C*H_3_), 15.2 (SO_3_CH_2_*C*H_3_) ppm; MALDI-TOF MS: *m*/*z* 1321.57 [M + Na]^+^ (Calcd. 1321.50); Anal. Calcd. for C_72_H_82_O_20_S (1298.51): C, 66.55; H, 6.36; O, 24.62; S, 2.47. Found: C, 66.50; H, 6.42; S, 2.51.

*Methyl [2,3,6-tri-O-benzyl-4-O-(2-naphthyl)methyl-α-d-glucopyranosyl]-(1→4)-[methyl (2-O-acetyl-3-O-methyl-α-l-idopyranosyl)uronate]-(1→4)-(2,3,6-tri-O-benzyl-α-d-glucopyranoside)* (**19**). To a solution of acceptor **12** (1.50 g, 2.10 mmol) and donor **16** (2.16 g, 3.16 mmol) in dry CH_2_Cl_2_ (20 mL) 4 Å molecular sieves (0.50 g) were added. The stirred mixture was cooled to −60 °C under argon and activated by method **B**. The reaction mixture was allowed to warm up to −50 °C for 30 min. The crude product was purified by column chromatography (7:3 *n*-hexane/EtOAc) to give **18** (2.41 g, 80%) as a white foam. *R*_f_ = 0.47 (6:4 *n*-hexane/EtOAc).

*Methyl [2,3-di-O-benzyl-6-deoxy-6-C-(ethylsulfonatomethyl)-α-d-glucopyranosyl]-(1→4)-[methyl (2-O-acetyl-3-O-methyl-α-l-idopyranosyl)uronate]-(1→4)-(2,3,6-tri-O-benzyl-α-d-glucopyranoside)* (**20**). Compound **17** (2.50 g, 1.92 mmol) was converted to **20** according to general method **D**. The crude product was purified by column chromatography (7:3 *n*-hexane/EtOAc) to give compound **20** (1.55 g, 70%) as a white foam; *R*_f_ = 0.40 (1:1 *n*-hexane/EtOAc); [α]_d_ +13.46 (*c* 0.56, CHCl_3_); IR ν_max_ (KBr): 3502, 3088, 3063, 3031, 2933, 1738, 1629, 1497, 1455, 1370, 1356, 1236, 1168, 1108, 1047, 1028, 917, 820, 740, 698, 606, 545, 466 cm^−1^; ^1^H-NMR (400 MHz, CDCl_3_) δ 7.38–7.19 (m, 25H, arom.), 5.15 (s, 1H, H-1-G), 4.93 (d, *J* = 11.4 Hz, 2H, BnC*H*_2_), 4.85–4.76 (m, 4H, H-2-G, H-5-G, H-1-F, BnC*H*_2_), 4.75–4.61 (m, 4H, BnC*H*_2_), 4.55 (m, 3H, H-1-H, BnC*H*_2_), 4.24 (q, *J* = 7.1 Hz, 2H, SO_3_C*H*_2_CH_3_), 3.94–3.79 (m, 3H, H-3-H, H-4-G), 3.77–3.59 (m, 6H, H-3-G, H-3-F, H-5-F, H-4-H, H-6_a,b_-H), 3.54 (dd, *J* = 9.3, 3.5 Hz, 1H, H-2-H), 3.43–3.31 (m, 2H, H-2-F, H-5-H), 3.40, 3.37, 3.34 (3s, 3 × C*H*_3_) 3.28–3.09 (m, 3H, H-4-F, H-7_a,b_), 2.48 (s, 1H, OH), 2.33–2.21 (m, 1H, H-6_a_-F), 1.93 (s, 3H, COC*H*_3_), 1.98–1.84 (m, 1H, H-6_b_-F), 1.35 (t, *J* = 7.1 Hz, 3H, SO_3_CH_2_C*H*_3_) ppm; ^13^C-NMR (101 MHz, CDCl_3_) δ 170.0, 169.3 (2 × *C*O), 139.1, 138.5, 138.1, 138.0, 137.9 (5C, *C*_q_ arom.), 128.6, 128.5, 128.4, 128.3, 128.1, 128.1, 128.0, 127.9, 127.8, 127.8, 127.7, 127.5, 127.3, 127.1 (25C, arom.), 98.5 (C-1-F), 98.0 (C-1-H), 97.7 (C-1-G), 80.6 (C-3-F), 80.1 (C-2-H), 79.9 (C-2-F), 79.8 (C-3-H), 79.8 (C-4-G), 76.5 (C-5-F), 74.8 (C-2-G), 74.4 (C-4-F), 75.0, 74.9, 73.4, 73.3, 73.2 (5 × Ph*C*H_2_), 70.1 (C-4-H), 69.8 (C-5-H), 68.5 (C-3-F), 68.4 (C-6-H), 68.0 (C-5-G), 66.2 (SO_3_*C*H_2_CH_3_), 58.4, 55.2, 51.7 (3 × O*C*H_3_), 46.3 (C-7-F), 25.8 (C-6-F), 21.0 (CO*C*H_3_), 15.1 (SO_3_CH_2_*C*H_3_) ppm; MALDI-TOF MS: *m*/*z* 1181.59 [M + Na]^+^ (Calcd. 1181.44); Anal. Calcd. for C_61_H_74_O_20_S (1158.45): C, 63.20; H, 6.43; O, 27.60; S, 2.77. Found: C, 63.25; H, 6.37; S, 2.72.

*Methyl [2,3,6-tri-O-benzyl-α-d-glucopyranosyl]-(1→4)-[methyl(2-O-acetyl-3-O-methyl-α-l-idopyranosyl)uronate]-(1→4)-2,3-di-O-benzyl-6-deoxy-6-C-(ethylsulfonatomethyl)-α-d-glucopyranoside* (**21**). Compound **18** (1.06 g, 0.83 mmol) was converted to **21** according to general method **D**. The crude product was purified by column chromatography (65:35 *n*-hexane/acetone) to give compound **21** (807 mg, 85%) as a white foam; *R*_f_ = 0.38 (65:35 *n*-hexane/acetone); [α]_d_ +14.44 (*c* 0.04, CHCl_3_); IR ν_max_ (KBr): 3481, 3063, 3031, 2929, 1740, 1626, 1497, 1455, 1370, 1234, 1167, 1105, 1046, 1028, 926, 820, 739, 698, 606, 548, 460, 418 cm^−1^; ^1^H-NMR (400 MHz, CDCl_3_) δ 7.30–7.08 (m, 25H, arom.), 5.17 (d, *J* = 3.3 Hz, 1H, H-1-G), 4.86–4.65 (m, 3H, H-1-F, H-2-G, H-5-G, BnC*H*_2_), 4.59 (m, 3H, BnC*H*_2_), 4.50–4.34 (m, 4H, H-1-H, BnC*H*_2_), 4.18 (q, *J* = 7.1 Hz, 2H, SO_3_C*H*_2_CH_3_), 3.85–3.14 (m, 13H, skeleton protons), 3.36, 3.29, 3.23 (3s, 9H, OC*H*_3_), 3.05–2.95 (m, 1H, H-7_b_-H), 2.46 (s, 1H, OH), 2.34–2.24 (m, 1H, H-6_a_-H), 1.93 (s, 3H, COC*H*_3_), 1.89–1.76 (m, 1H, H-6_b_-H), 1.28 (t, *J* = 7.1 Hz, 3H, SO_3_CH_2_C*H*_3_) ppm; ^13^C-NMR (101 MHz, CDCl_3_) δ 170.2, 169.6 (2 × *C*O), 139.0, 138.8, 138.2, 138.1, 138.0 (5C, *C*_q_ arom.), 128.6, 128.5, 128.4, 128.2, 128.0, 127.9, 127.8, 127.8, 127.3 (25C, arom.), 99.5 (C-1-F), 98.1 (C-1-H), 98.0 (C-1-G), 81.1, 80.2, 79.5, 79.5, 78.9, 77.0, 75.0, 71.2, 70.9, 69.8 (12C, skeleton carbons), 75.3, 75.1,73.7, 73.6, 73.3 (5 × Ph*C*H_2_), 69.3 (C-6-F), 69.0, 68.2, 66.3 (SO_3_*C*H_2_CH_3_), 58.9, 55.6, 51.9 (3 × O*C*H_3_), 46.8 (C-7-H), 25.9 (C-6-H), 21.1 (CO*C*H_3_), 15.2 (SO_3_*C*H_2_CH_3_) ppm; MALDI-TOF MS: *m*/*z* 1181.64 [M + Na]^+^ (Calcd. 1181.44); Anal. Calcd. for C_61_H_74_O_20_S (1158.45): C, 63.20; H, 6.43; O, 27.60; S, 2.77. Found: C, 63.34; H, 6.47; S, 2.79.

*Methyl [2,3,6-tri-O-benzyl-α-d-glucopyranosyl]-(1→4)-[methyl-(2-O-acetyl-3-O-methyl-α-l-idopyranosyl)uronate]-(1→4)-(2,3,6-tri-O-benzyl-α-d-glucopyranoside)* (**22**) [15]. Compound **19** (2.4 g, 1.87 mmol) was converted to **22** according to general method **D**. The crude product was purified by column chromatography (7:3 *n*-hexane/EtOAc) to give compound **22** (1.26 g, 59%) as a white foam; *R*_f_ = 0.33 (6:4 *n*-hexane/EtOAc); [α]_d_ +8.41 (*c* 0.62, CHCl_3_) (lit. [15] [α]_d_ +2.3 (*c* 0.10, CHCl_3_); IR ν_max_ (KBr): 3087, 3062, 3031, 2932, 2906, 1739, 1605, 1497, 1455, 1371, 1237, 1104, 1046, 1028, 908, 738, 697, 606, 538, 459, 419 cm^−1^. The NMR spectroscopic and analytical data of **22** are consistent with those given in the literature [15].

*Methyl [2,3,4-tri-O-methyl-6-deoxy-6-C-(ethylsulfonatomethyl)-α-d-glucoopiranosyl]-(1→4)-[2,3-di-O-acetyl-6-O-(2-naphthyl)methyl-β-d-glucopyranosyl]-(1→4)-[2,3-di-O-benzyl-6-deoxy-6-C-(ethylsulfonatomethyl)-α-d-glucopyranosyl]-(1→4)-[methyl (2-O-acetyl-3-O-methyl-α-l-idopyranosyl)uronate]-(1→4)-2,3,6-tri-O-benzyl-α-d-gluco-pyranoside* (**6**). To a solution of acceptor **20** (630 mg, 0.54 mmol) and donor **23** (660 mg, 0.82 mmol) in dry CH_2_Cl_2_ (20 mL), 4 Å molecular sieves (0.50 g) were added. The stirred mixture was cooled to −40 °C under argon and activated by method **C**. The reaction mixture was allowed to warm up to −15 °C for 4 h. The crude product was purified by column chromatography (6:4 *n*-hexane/EtOAc) to give **6** (626 mg, 62%) as a colourless syrup; *R*_f_ = 0.26 (1:1 *n*-hexane/EtOAc); [α]_d_ +23.69 (*c* 0.14, CHCl_3_); ^1^H-NMR (400 MHz, CDCl_3_) δ 7.84–7.75 (m, 3H, arom.), 7.65 (s, 1H, arom.), 7.48–7.40 (m, 2H, arom.), 7.37–7.13 (m, 26H, arom.), 5.18 (t, *J* = 9.2 Hz, 1H, H-3-E), 5.11 (s, 1H, H-1-G), 4.99–4.46 (m, 19H, H-1-D, H-2-E, H-5-G, H-2-G, H-1-E, H-1-H, H-1-F, 12 × ArC*H*_2_), 4.27, 4.07 (2q, 4H, SO_3_C*H*_2_CH_3_), 3.93–3.77 (m, 5H, H-4-H, H-4-E, H-3-F, H-4-G, H-3-H), 3.76–3.59 (m, 5H, H-5-H, H-5-F, H-6_a,b_-H, H-3-G), 3.58–3.17 (m, 9H, H-2-F, H-6_a,b_-E, H-5-D, H-4-F, H-2-H, H-3-D, H-5-E, H-7_a_-F), 3.52, 3.48, 3.40, 3.36, 3.33, 3.31 (6s, 18H, 6 × OC*H*_3_), 3.18–3.03 (m, 2H, H-7_b_-F, H-7_a_-D), 2.93–2.81 (m, 2H, H-2-D, H-7_b_-D), 2.65 (t, *J* = 9.2 Hz, 1H, H-4-D), 2.33–2.21 (m, 1H, H-6_a_-F), 2.21–2.08 (m, 1H, H-6_a_-D), 2.05, 1.99, 1.94 (3s, 9H, 3 × COC*H*_3_), 1.86–1.71 (m, 2H, H-6_b_-D, H-6_b_-F), 1.38, (t, *J* = 7.1 Hz, 3H, SO_3_CH_2_C*H*_3_), 1.25 (t, *J* = 6.8 Hz, 3H, SO_3_C*H*_2_CH_3_) ppm; ^13^C-NMR (101 MHz, CDCl_3_) δ 170.1, 169.8, 169.8, 169.3 (4 × *C*O), 139.1, 138.9, 138.0, 137.9, 137.7, 135.3, 133.2, 132.9 (8C, *C*_q_ arom.), 128.4, 128.3, 128.2, 128.1, 128.0, 128.0, 127.9, 127.8, 127.8, 127.6, 127.5, 127.1, 127.0, 126.4, 126.1, 125.8, 125.6 (32C, arom.), 101.1 (C-1-H), 98.0 (C-1-E), 97.8 (C-1-F), 97.4 (C-1-G), 96.8 (C-1-D), 83.2 (C-4-D), 82.3 (C-3-D), 82.1 (C-4-F), 81.9 (C-2-D), 80.0 (C-2-F), 79.7 (C-2-H), 79.6 (C-4-G), 79.4 (C-3-F), 76.0 (C-3-G), 75.1 (C-5-E), 74.7, 74.3, 73.6, 73.4, 73.2, 73.2, (6 × Ar*C*H_2_), 74.5 (C-3-E), 74.5 (C-4-H), 74.4 (C-4-E), 74.0 (C-3-H), 72.5 (C-2-E), 70.0 (C-5-H), 69.2 (C-5-D), 68.9 (C-5-F), 68.3 (C-6-H), 68.2 (C-5-G), 67.7 (C-6-E), 67.3 (C-2-G), 66.2, 65.8 (2 × SO_3_*C*H_2_CH_3_), 60.5, 60.5, 58.9, 58.1, 55.1, 51.7 (6 × O*C*H_3_), 46.6 (C-7-D), 46.4 (C-7-F), 26.0 (C-6-D), 25.7 (C-6-F), 21.0, 20.8, 20.5 (3 × CO*C*H_3_), 15.0, 15.0 (2 × SO_3_CH_2_*C*H_3_) ppm; MALDI-TOF MS: *m*/*z* 1877.77 [M + Na]^+^ (Calcd. 1877.68); Anal. Calcd. for C_94_H_118_O_34_S_2_ (1854.69): C, 60.83; H, 6.41; O, 29.31; S, 3.46. Found: C, 60.69; H, 6.35; S, 3.41.

*Methyl [2,3,4-tri-O-methyl-6-deoxy-6-C-(ethylsulfonatomethyl)-α-d-glucopyranosyl]-(1→4)-[2,3-di-O-acetyl-6-O-(2-naphthyl)methyl-β-d-glucopyranosyl]-(1→4)-[2,3,6-tri-O-benzyl-α-d-glucopyranosyl]-(1→4)-[methyl (2-O-acetyl-3-O-methyl-α-l-idopyranosyl)uronate]-(1→4)-2,3-di-O-benzyl-6-deoxy-6-C-(ethylsulfonatomethyl)-α-d-glucopyranoside* (**7**). To a solution of acceptor **21** (720 mg, 0.62 mmol) and donor **23** (752 mg, 0.93 mmol) in dry CH_2_Cl_2_ (20 mL) 4 Å molecular sieves (0.50 g) were added. The stirred mixture was cooled to −50 °C under argon and activated by method **B**. The reaction mixture was allowed to warm up to −20 °C for 3 h. The crude product was purified by column chromatography (65:35 *n*-hexane/EtOAc) to give **7** (726 mg, 63%) as a colourless syrup; *R*_f_ = 0.21 (1:1 *n*-hexane/EtOAc); [α]_d_ +32.21 (*c* 0.14, CHCl_3_); ^1^H-NMR (400 MHz, CDCl_3_) δ 7.83–7.71 (m, 3H, arom.), 7.62 (s, 1H, arom.), 7.44 (m, 2H, arom.), 7.41–7.12 (m, 26H, arom.), 5.21 (d, *J* = 2.3 Hz, 1H, H-1-G), 5.10–4.99 (m, 1H, H-3-E, ArC*H*_2_), 4.96–4.76 (m, 6H, H-1-D, H-2-G, H-2-E, H-1-F, 2 × ArC*H*_2_), 4.75–4.63 (m, 5H, H-5-G, 4 × ArC*H*_2_), 4.60–4.38 (m, 7H, H-1-E, H-1-H, 5 × ArC*H*_2_), 4.29 (q, *J* = 7.1 Hz, 2H, SO_3_C*H*_2_CH_3_), 4.06 (q, *J* = 7.1 Hz, 2H, SO_3_C*H*_2_CH_3_), 3.95 (t, *J* = 9.4 Hz, 1H, H-4-H), 3.91–3.67 (m, 8H, H-4-G, H-4-E, H-6_a_-E, H-5-H, H-3-H, H-3-F, H-6_a_-F, H-5-F), 3.67–3.25 (m, 9H, H-3-G, H-6_b_-F, H-6_b_-E, H-4-F, H-5-D, H-2-H, H-2-F, H-3-D, H-7-H), 3.57, 3.53, 3.43, 3.41, 3.33 (5s, 15H, 5 × OC*H*_3_), 3.23–3.04 (m, 3H, H-5-E, H-7_a_-D, H-7_a_-H), 2.97–2.87 (m, 2H, H-2-D, H-7_b_-D), 2.69 (t, *J* = 9.2 Hz, 1H, H-4-D), 2.44–2.30 (m, 1H, H-6_a_-H), 2.30–2.14 (m, 1H, H-6_a_-D), 2.02, 1.96, 1.95 (3s, 9H, 3 × COC*H*_3_), 2.06–1.74 (m, 2H, H-6_b_-D, H-6_b_-H), 1.40 (t, *J* = 7.1 Hz, 3H, SO_3_CH_2_C*H*_3_), 1.23 (t, *J* = 7.4 Hz, 3H, SO_3_CH_2_C*H*_3_) ppm. ^13^C-NMR (101 MHz, CDCl_3_) δ 170.3, 170.0, 169.7, 169.6 (4 × *C*O), 139.4, 139.0, 138.3, 138.1, 137.6, 135.8, 133.4, 133.0 (8C, *C*_q_ arom.), 128.8, 128.6, 128.5, 128.4, 128.4, 128.2, 128.2, 128.1, 128.0, 127.9, 127.8, 127.6, 127.3, 126.4, 126.1, 125.9, 125.8 (32C, arom.), 99.8 (C-1-E), 99.6 (C-1-F), 98.0 (C-1-H), 97.8 (C-1-G), 96.9 (C-1-D), 83.5 (C-4-D), 82.7 (C-3-D), 82.2 (C-2-D), 80.3 (C-4-F), 79.7 (C-3-H), 79.4 (C-3-F), 79.3 (C-2-F), 78.6 (C-2-H), 76.7 (C-4-H), 76.5 (C-3-G), 75.3 (C-5-E), 74.9 (C-4-G), 74.9 (C-3-E), 74.9 (C-4-E), 72.5 (C-2-E), 71.1 (C-5-F), 69.5 (C-5-D), 69.3 (C-5-G), 68.2 (C-5-H), 68.2 (C-2-G), 75.3, 75.1, 73.9, 73.7, 73.6, 73.6 (6 × Ar*C*H_2_), 68.1 (C-6-E), 67.4 (C-6-F), 66.3, 66.1 (2 × SO_3_*C*H_2_CH_3_), 60.8, 60.8, 59.2, 58.8, 55.6, 52.0 (6 × O*C*H_3_), 46.8, 46.8 (C-7-D, C-7-H), 26.2 (C-6-D), 26.0 (C-6-H), 21.1, 21.0, 20.8 (3 × CO*C*H_3_), 15.2, 15.1 (2 × SO_3_CH_2_*C*H_3_) ppm; MALDI-TOF MS: *m*/*z* 1877.77 [M + Na]^+^ (Calcd. 1877.68); Anal. Calcd. for C_94_H_118_O_34_S_2_ (1854.69): C, 60.83; H, 6.41; O, 29.31; S, 3.46. Found: C, 60.90; H, 6.44; S, 3.51.

*Methyl [2,3,4-tri-O-methyl-6-deoxy-6-C-(ethylsulfonatomethyl)-α-d-glucopyranosyl]-(1→4)-[2,3-di-O-acetyl-6-O-6-O-(2-naphthyl)methyl-β-d-glucopyranosyl]-(1→4)-[2,3,6-tri-O-benzyl-α-d-glucopyranosyl]-(1→4)-[methyl-(2-O-acetyl-3-O-methyl-α-l-idopyranosyl)uronate]-(1→4)-(2,3,6-tri-O-benzyl-α-d-glucopyranoside)* (**8**). To a solution of acceptor **22** (1.18 g, 1.03 mmol) and donor **23** (1.25 g, 1.55 mmol) in dry CH_2_Cl_2_ (40 mL), 4 Å molecular sieves (1 g) were added. The stirred mixture was cooled to −50 °C under argon and activated by method **C**. The reaction mixture was allowed to warm up to 0 °C for 4 h. The crude product was purified by column chromatography (6:4 *n*-hexane/EtOAc) to give **8** (1.29 g, 68%) as a colourless syrup; *R*_f_ = 0.51 (94:6 CH_2_Cl_2_/acetone); [α]_d_ +24.44 (*c* 0.05, CHCl_3_); ^1^H-NMR (400 MHz, CDCl_3_) δ 7.86–7.71 (m, 3H, arom.), 7.62 (s, 1H, arom.), 7.44 (m, 2H, arom.), 7.40–7.11 (m, 31H, arom.), 5.15 (d, *J* = 2.7 Hz, 1H, H-1-G), 5.05 (m, 2H, H-3-E, ArC*H*_2_), 4.95–4.75 (m, 7H, H-1-D, H-2-E, H-1-F, H-2-G, 3 × ArC*H*_2_), 4.74–4.65 (m, 5H, H-5-G, 4 × ArC*H*_2_), 4.62–4.46 (m, 6H, H-1-H, 5 × ArC*H*_2_), 4.45–4.40 (m, 2H, H-1-E, ArC*H*_2_), 4.05 (q, *J* = 7.1 Hz, 2H, SO_3_C*H*_2_CH_3_), 3.95 (t, *J* = 9.4 Hz, 1H, H-4-F), 3.91–3.45 (m, 16H, H-4-G, H-4-H, H-4-E, H-3-H, H-5-F, H-6_a,b_-H, H-3-F, H-5-H, H-3-G, H-6_a,b_-E, H-6_a,b_-F, H-5-D, H-2-H), 3.57, 3.53, 3.41, 3.35, 3.34, 3.32 (6s, 18H, 6 × OC*H*_3_), 3.44–3.27 (m, 4H, H-2-F, H-3-D, H-5-E, H-7_a_-D), 2.97–2.85 (m, 2H, H-2-D, H-7_b_-D), 2.69 (t, *J* = 9.2 Hz, 1H, H-4-D), 2.26–2.15 (m, 1H, H-6_a_-D), 2.01, 1.91, 1.89 (3s, 9H, 3 × COC*H*_3_), 1.87–1.75 (m, 1H, H-6_b_-D), 1.23 (t, *J* = 7.1 Hz, 3H, SO_3_CH_2_C*H*_3_) ppm; ^13^C-NMR (91 MHz, CDCl_3_) δ 170.2, 170.0, 169.7, 169.6 (4 × *C*O), 139.5, 139.2, 138.3, 138.3, 138.1, 137.7, 135.8, 133.4, 133.0 (9C, *C*_q_ arom.), 128.8, 128.5, 128.4, 128.2, 128.2, 128.0, 127.9, 127.8, 127.8, 127.6, 127.5, 127.3, 127.1, 126.4, 126.1, 125.9, 125.8 (37C, arom.), 99.8 (C-1-E), 99.7 (C-1-F), 98.1 (C-1-H), 97.8 (C-1-G), 96.9 (C-1-D), 83.6 (C-4-D), 82.7 (C-3-D), 82.2 (C-2-D), 80.1 (C-2-H), 79.9 (C-3-H), 79.8 (C-3-F), 79.3 (C-2-F), 76.8 (C-3-G), 76.5 (C-4-F), 75.3 (C-5-E), 75.1 (C-3-E), 75.1 (C-4-E), 74.9 (C-4-G), 74.9 (C-4-H), 75.1, 75.1, 75.1, 73.8, 73.6, 73.5, 73.5 (7 × Ar*C*H_2_), 72.5 (C-2-E), 71.0 (C-5-F), 70.2 (C-5-H), 69.5 (C-5-D), 69.2 (C-5-G), 68.5 (C-2-G), 68.3 (C-6-F), 68.3 (C-6-E), 67.4 (C-6-H), 66.0 (SO_3_*C*H_2_CH_3_), 60.8, 60.8, 59.2, 58.8, 55.3, 51.8 (6 × O*C*H_3_), 46.8 (C-7-D), 26.2 (C-6-D), 21.1, 21.1, 20.8 (3 × CO*C*H_3_), 15.1 (SO_3_CH_2_*C*H_3_) ppm; MALDI-TOF MS: *m*/*z* 1862.75 [M + Na]^+^ (Calcd. 1863.02); Anal. Calcd. for C_98_H_118_O_32_S (1840.03): C, 63.97; H, 6.46; O, 27.82; S, 1.74. Found: C, 64.03; H, 6.49; S, 1.68.

*Methyl-[6-O-benzyl-2,3,4-tri-O-methyl-α-d-glucopyranosyl]-(1→4)-[2,3-di-O-acetyl-6-O-(2-naphthyl)methyl-β-d-glucopyranosyl]-(1→4)-[2,3-di-O-benzyl-6-deoxy-6-C-(ethylsulfonatomethyl)-α-d-glucopyranosyl]-(1→4)-[methyl-(2-O-acetyl-3-O-methyl-α-l-idopyranosyl)uronate]-(1→4)-(2,3,6-tri-O-benzyl-α-d-glucopyranoside)* (**9**). To a solution of acceptor **20** (1.20 g, 1.04 mmol) and donor **24** (1.23 g, 1.55 mmol) in dry CH_2_Cl_2_ (40 mL), 4 Å molecular sieves (1 g) were added. The stirred mixture was cooled to −35 °C under argon and activated by method **C**. The reaction mixture was allowed to warm up to −20 °C for 4 h. The crude product was purified twice by column chromatography (**I**. 6:4 *n*-hexane/EtOAc **II**. 96:4 CH_2_Cl_2_/EtOAc) to give **9** (1.20 g, 63%) as colourless; *R*_f_ = 0.33 (1:1 *n*-hexane/EtOAc); [α]_d_ +26.28 (*c* 0.06, CHCl_3_); ^1^H-NMR (400 MHz, CDCl_3_) δ 7.84–7.71 (m, 3H, arom.), 7.63 (s, 1H, arom.), 7.50–7.41 (m, 2H, arom.), 7.38–7.11 (m, 31H, arom), 5.22 (t, *J* = 9.3 Hz, 1H, H-3-E), 5.12–5.03 (m, 2H, H-1-G, H-1-D), 4.95 (t, *J* = 12.3 Hz, 2H, 2 × ArC*H*_2_), 4.89–4.48 (m, 15H, H-2-E, H-5-G, H-2-G, H-1-E, H-1-F, H-1-H, 9 × ArC*H*_2_), 4.40 (d, *J* = 10.9 Hz, 2H, 2 × ArC*H*_2_), 4.26 (m, 3H, SO_3_C*H*_2_CH_3_, ArC*H*_2_), 3.98–3.43 (m, 14H, H-4-E, H-4-H, H-4-G, H-3-F, H-3-H, H-5-F, H-5-H, H-6_a,b_-H, H-3-G, H-6_a,b_-E, H-2-H, H-5-D), 3.55, 3.39, 3.39, 3.38, 3.34, 3.30 (6s, 18H, 6 × OC*H*_3_) 3.43–2.99 (m, 9H, H-6_a,b_-D, H-4-F, H-5-E, H-2-F, H-3-D, H-7_a,b_-F, H-4-D, H-2-D), 2.31–2.20 (m, 1H, H-6_a_-F), 2.04, 1.99, 1.93 (3s, 9H, 3 × OC*H*_3_), 1.85–1.70 (m, 1H, H-6_b_-F), 1.39 (t, *J* = 7.1 Hz, 3H, SO_3_CH_2_C*H*_3_) ppm; ^13^C-NMR (91 MHz, CDCl_3_) δ 170.3, 170.0, 169.9, 169.5 (4 × *C*O), 139.2, 139.1, 138.2, 138.1, 138.1, 137.9, 136.0, 133.3, 133.0 (9C, *C*_q_ arom.), 128.5, 128.5, 128.4, 128.4, 128.3, 128.2, 128.1, 128.0, 127.8, 127.7, 127.3, 127.2, 126.7, 126.2, 126.1, 125.9, 125.6 (37C, arom.), 101.1 (C-1-E), 98.3 (C-1-F), 98.1 (C-2-H), 97.9 (C-1-D), 97.6 (C-1-G), 83.2 (C-3-D), 82.1 (C-4-F), 81.8 (C-2-D), 80.2 (C-2-H), 79.9 (C-3-H), 79.8 (C-2-F), 79.5 (C-3-F), 79.3 (C-4-D), 76.1 (C-3-G), 75.1 (C-5-E), 75.0 (C-3-E), 74.8 (C-4-H), 74.5 (C-4-E), 74.2 (C-4-G), 74.9, 74.6, 73.8, 73.5, 73.4, 73.4, 73.4 (7 × Ar*C*H_2_), 72.9 (C-2-E), 71.2 (C-5-D), 70.2 (C-5-F), 69.1 (C-5-H), 68.6 (C-6-E), 68.5 (C-6-H), 68.4 (C-5-G), 68.3 (C-6-D), 67.4 (C-2-G), 66.3 (SO_3_*C*H_2_CH_3_), 60.7, 60.4, 59.4, 58.2, 55.3, 51.9 (6 × O*C*H_3_), 46.6 (C-7-F), 25.9 (C-6-F), 21.2, 21.0, 20.7 (3 × CO*C*H_3_), 15.2 (SO_3_CH_2_*C*H_3_) ppm; MALDI-TOF MS: *m*/*z* 1862.74 [M + Na]^+^ (Calcd. 1863.02); Anal. Calcd. for C_98_H_118_O_32_S (1840.03): C, 63.97; H, 6.46; O, 27.82; S, 1.74. Found: C, 63.95; H, 6.51; S, 1.78.

*Methyl [6-O-benzyl-2,3,4-tri-O-methyl-α-d-glucopyranosyl]-(1→4)-[2,3-di-O-acetyl-6-O-(2-naphthyl)methyl-β-d-glucopyranosyl]-(1→4)-[2,3,6-tri-O-benzyl-α-d-glucopyranosyl]-(1→4)-[methyl-(2-O-acetyl-3-O-methyl-α-l-idopyranosyl)uronate]-(1→4)-2,3-di-O-benzyl-6-deoxy-6-C-(ethylsulfonatomethyl)-α-d-glucopyranoside* (**10**). To a solution of acceptor **21**. (720 mg, 0.62 mmol) and donor **24** (737 mg, 0.93 mmol) in dry CH_2_Cl_2_ (20 mL), 4 Å molecular sieves (0.50 g) were added. The stirred mixture was cooled to −40 °C under argon and activated by method **C**. The reaction mixture was allowed to warm up to −10 °C for 90 min. The crude product was purified twice by column chromatography (**I**. 9:1 CH_2_Cl_2_/EtOAc **II**. 6:4 *n*-hexane/EtOAc) to give **10** (825 mg, 72%) as a colourless syrup; *R*_f_ = 0.31 (1:1 *n*-hexane/EtOAc); [α]_d_ +35.45 (*c* 0.05, CHCl_3_); ^1^H-NMR (400 MHz, CDCl_3_) δ 7.82–7.69 (m, 3H, arom.), 7.62 (s, 1H, arom.), 7.48–7.09 (m, 30H, arom.), 5.21 (d, *J* = 5.0 Hz, 1H, H-1-G), 5.12–5.03 (m, 3H, H-3-E, H-1-D, ArC*H*_2_), 4.92–4.76 (m, 5H, H-2-E, H-1-F, H-2-G, 2 × ArC*H*_2_), 4.75–4.61 (m, 5H, H-5-G, 4 × ArC*H*_2_), 4.59–4.51 (m, 3H, 3 × ArC*H*_2_), 4.49–4.37 (m, 5H, H-1-E, H-1-H, 3 × ArC*H*_2_), 4.34–4.25 (m, 3H, SO_3_C*H*_2_CH_3_, ArC*H*_2_), 4.01–3.84 (m, 3H, H-4-H, H-4-E, H-4-G), 3.83–3.67 (m, 7H, H-3-H, H-5-F, H-6_a_-F, H-6_a,b_-E, H-5-H, H-3-F), 3.66–3.52 (m, 3H, H-3-G, H-5-D, H-6_b_-F), 3.50–3.26 (m, 7H, H-6_a,b_-D, H-2-H, H-4-F, H-3-D, H-2-F, H-7_a_-H), 3.59, 3.44, 3.42, 3.41, 3.33, 3.32 (6s, 18H, 6 × OC*H*_3_), 3.26–3.02 (m, 4H, H-5-E, H-4-D, H-7_a_-H, H-2-D), 2.42–2.32 (m, 1H, H-6_a_-H), 2.01, 1.96, 1.94 (3s, 9H, 3 × COC*H*_3_),1.98–1.93 (m, 1H, H-7_b_-H) 1.39 (t, *J* = 12.7 Hz, 3H, SO_3_CH_2_C*H*_3_) ppm; ^13^C-NMR (101 MHz, CDCl_3_) δ 170.3, 170.0, 169.7, 169.6 (4 × *C*O), 139.4, 139.0, 138.4, 138.1, 138.1, 137.7, 136.2, 133.4, 133.0 (9C, *C*_q_ arom.), 128.8, 128.6, 128.4, 128.4, 128.3, 128.2, 128.1, 128.0, 127.8, 127.7, 127.6, 127.3, 127.2, 126.0, 125.9, 125.7 (37C, arom.), 99.8 (C-1-E), 99.6 (C-1-F), 98.0 (C-1-H), 97.9 (C-1-G), 97.9 (C-1-D), 83.3 (C-3-D), 81.9 (C-2-D), 80.3 (C-4-F), 79.8 (C-5-H), 79.8 (C-3-F), 79.4 (C-4-D), 79.4 (C-3-H), 79.2 (C-2-F), 78.6 (C-2-H), 76.7 (C-3-G), 76.5 (C-4-H), 75.1 (C-4-G), 75.1 (C-4-E), 75.1 (C-5-E) 74.9 (C-3-E), 75.3, 75.1, 73.9, 73.7, 73.6, 73.4, 73.4 (7 × Ar*C*H_2_), 72.7 (C-2-E), 71.4 (C-5-D), 71.1 (C-5-F), 69.3 (C-5-G), 68.9(C-6-E), 68.6 (C-6-D), 68.2 (C-2-G), 67.4 (C-6-F), 66.2 (SO_3_*C*H_2_CH_3_), 60.8, 60.5, 59.3, 58.8, 55.6, 51.9 (6 × O*C*H_3_), 46.8 (C-7-H), 26.1 (C-6-H), 21.1, 21.0, 20.8 (3 × CO*C*H_3_), 15.2 (SO_3_CH_2_*C*H_3_) ppm; ESI-MS: *m*/*z* 1862.75 [M + Na]^+^ (Calcd. 1863.02); Anal. Calcd. for C_98_H_118_O_32_S (1838,73): C, 63.97; H, 6.46; O, 27.82; S, 1.74. Found: C, 64.07; H, 6.53; S, 1.82.

*Methyl [6-O-benzyl-2,3,4-tri-O-methyl-α-d-glucopyranosyl]-(1→4)-[6-O-(2-naphthyl)-methyl-β-d-glucopyranosyl]-(1→4)-[sodium (2,3-di-O-benzyl-6-deoxy-6-C-sulfonato-methyl-α-d-glucopyranosyl)]-(1→4)-[methyl (3-O-methyl-α-l-idopyranosyl)uronate]-(1→4)-(2,3,6-tri-O-benzyl-α-d-glucopyranoside)* (**25**). NaOMe (36 mg) was added to the solution of compound **9** (1.22, 0.66 mmol) in MeOH (35 mL) at room temperature and stirred for 24 h. The reaction mixture was quenched by the addition of acetic acid (1–2 drops) and the solution was concentrated. Then, NaI (149 mg, 0.99 mmol) was added to the solution of crude product in acetone (40 mL) and the mixture was stirred at room temperature for 24 h. The mixture was concentrated and purified by gel chromatography (Sephadex LH-20, MeOH) to give **25** (919 mg, 81% for two steps) as a colourless syrup; *R*_f_ = 0.28 (95:5 CH_2_Cl_2_/MeOH); [α]_D_ +33.7 (*c* 0.11, CHCl_3_); ^1^H-NMR (CDCl_3_, 400 MHz): δ (ppm) 7.79–7.07 (m, 37H, arom.), 5.34–4.21 (m, 19H, 7 × ArC*H*_2_, 5 × H-1), 3.98–2.87 (m, 49H, 20 × skeleton protons, 6 × OC*H*_3_, 3 × H-6_a,b_, H-7_a,b_, 3 × O*H*), 2.43–2.38 (m, 1H, H-6_a_-F), 2.13–2.03 (m, 1H, H-6_b_-F); ^13^C-NMR (CDCl_3_, 100 MHz): δ (ppm) 171.3 (*C*O), 140.7, 140.1, 139.4, 139.3, 138.9, 137.4, 134.6, 134.3 (9 × *C*_q_ arom.), 129.4–126.8 (37C, arom.), 104.3, 102.2, 99.5, 98.9, 96.0 (5 × C-1), 84.6, 83.5, 81.7, 81.3, 81.2, 80.7, 80.3, 79.7, 78.0, 76.3, 75.9, 75.6, 72.6, 72.3, 71.6, 70.8, 69.2, 67.9 (20C, skeleton carbons), 75.7, 74.9, 74.4, 74.2, 73.9 (7 × Ar*C*H_2_), 69.8 (3 × C-6) 60.9, 60.8, 59.8, 58.5 (4 × O*C*H_3_), 55.5 (C-1-O*C*H_3_), 52.9 (COO*C*H_3_), 48.2 (C-7-F), 27.6 (C-6-F); MALDI-TOF MS: *m*/*z* 1729.72 [M + Na]^+^ (Calcd. 1729.64). Anal. Calcd. for C_90_H_107_NaO_29_S (1707.85): C, 63.29; H, 6.31; S, 1.88. Found: C, 63.37; H, 6.40; S, 1.97.

*Methyl [6-O-benzyl-2,3,4-tri-O-methyl-α-d-glucopyranosyl]-(1→4)-[2,3-di-O-methyl-6-O-(2-naphthyl)methyl-β-d-glucopyranosyl]-(1→4)-[sodium (2,3-di-O-benzyl-6-deoxy-6-C-sulfonatomethyl-α-d-glucopyranosyl)]-(1→4)-[methyl (2,3-di-O-methyl-α-l-idopyranosyl)uronate]-(1→4)-(2,3,6-tri-O-benzyl-α-d-glucopyranoside)* (**26**). An amount of 60 m/m% NaH (68 mg, 1.68 mmol) was added to the solution of compound **25** (798 mg, 0.467 mmol) in dry *N*,*N*-dimethylmethanamide (DMF) (15 mL) at 0 °C. After 30 min of stirring at room temperature, MeI (105 μL, 1.68 mmol) was added to the reaction mixture and it was stirred for 4 h. The reaction mixture was quenched by the addition of MeOH (4–5 drops). The solution was concentrated and the crude product was purified by gel chromatography (Sephadex LH-20 in MeOH) to give **26** (550 mg, 67%) as a colourless syrup; *R*_f_ = 0.58 (9:1 CH_2_Cl_2_/MeOH); [α]_D_ +49.4 (*c* 0.26, CHCl_3_); ^1^H-NMR (CDCl_3_, 400 MHz): δ (ppm) 7.79–7.12 (m, 37H, arom.), 5.58 (d, *J* = 3.6 Hz, 1H), 5.21 (d, *J* = 5.6 Hz, 1H), 5.00–4.33 (m, 17H), 3.86–2.98 (m, 55H, 20 × skeleton protons, 9 × OC*H*_3_, 3 × H6_a,b_, H-7_a,b_), 2.48–2.39 (m, 1H, H-6_a_-F), 2.02–2.01 (m, 1H, H-6_b_-F); ^13^C-NMR (CDCl_3_, 100 MHz): δ (ppm) 169.8 (*C*O), 139.3, 138.9, 138.3, 138.2, 138.1, 138.0, 136.1, 133.2, 132.8 (9 × *C*_q_ arom.), 128.3–125.6 (37C, arom.), 103.0, 100.1, 99.7, 98.0, 96.0 (5 × C-1), 86.3, 84.8, 83.3, 81.6, 80.7, 80.2, 79.8, 79.6, 79.5, 79.1, 78.3, 76.4, 74.3, 74.2, 72.0, 71.5, 70.7, 70.2, 69.9, 69.6 (20C, skeleton carbons), 75.2, 75.1, 74.6, 73.5, 73.3, 73.2, 72.9 (7 × Ar*C*H_2_), 69.1, 68.3, 68.0 (3 × C-6), 60.6, 60.4, 60.2, 59.7, 59.6, 59.4, 59.3 (7 × O*C*H_3_), 55.2 (C-1-O*C*H_3_), 51.7 (COO*C*H_3_), 46.8 (C-7-F), 26.4 (C-6-F); MALDI-TOF MS: *m*/*z* 1771.76 [M + Na]^+^ (Calcd. 1771.69). Anal. Calcd. for C_93_H_113_NaO_29_S (1749.93): C, 63.83; H, 6.51; S, 1.83. Found: C, 63.92; H, 7.03; S, 2.01.

*Methyl [6-O-benzyl-2,3,4-tri-O-methyl-α-d-glucopyranosyl]-(1→4)-[2,3-di-O-methyl-β-d-glucopyranosyl]-(1→4)-[sodium (2,3-di-O-benzyl-6-deoxy-6-C-sulfonatomethyl-α-d-glucopyranosyl)]-(1→4)-[methyl (2,3-di-O-methyl-α-l-idopyranosyl)uronate]-(1→4)-(2,3,6-tri-O-benzyl-α-d-glucopyranoside)* (**27**). Compound **26** (550 mg, 0.314 mmol) was converted to **27** according to general method **D**. The crude product was purified by column chromatography (9:1 CH_2_Cl_2_/MeOH) to give **27** (404 mg, 80%) as a colourless syrup; *R*_f_ = 0.52 (9:1 CH_2_Cl_2_/MeOH); [α]_D_ +24.2 (*c* 0.06, CHCl_3_); ^1^H-NMR (CDCl_3_, 400 MHz): δ (ppm) 7.35–7.24 (m, 30H, arom.), 5.50 (s, 1H), 5.18 (s, 2H), 4.82–4.57 (m, 15H), 3.94–3.15 (m, 55H, 20 skeleton protons, 9 × OC*H*_3_, 3 × H-6_a,b_, H-7_a,b_), 2.52–2.41 (m, 1H, H-6_a_-F), 2.08–1.99 (m, 1H, H-6_b_-F); ^13^C-NMR (CDCl_3_, 100 MHz): δ (ppm) 171.7 (*C*O), 140.3–139.5 (6 × *C*_q_ arom.), 129.5–128.4 (30C, arom.), 103.5, 100.6, 99.0, 97.8, 97.0 (5 × C-1), 87.6, 86.4, 84.4, 83.0, 81.7, 81.4, 80.9, 80.6, 80.5, 80.4, 79.7, 76.8, 76.6 (20C, skeleton carbons), 76.6, 76.0, 74.9, 74.5, 74.1 (6 × Ar*C*H_2_), 70.0, 69.7, 62.8 (3 × C-6), 61.0, 60.8, 60.6, 59.9, 59.7 (7 × O*C*H_3_), 55.6 (C-1-O*C*H_3_), 52.6 (COO*C*H_3_), 49.0 (C-7-F), 27.8 (C-6-F); MALDI-TOF MS: *m*/*z* 1631.77 [M + Na]^+^ (Calcd. 1631.63). Anal. Calcd. for C_82_H_105_NaO_29_S (1609.75): C, 61.18; H, 6.57; S, 1.99. Found: C, 61.24; H, 6.59; S, 2.08.

*Methyl-(6-O-benzyl-2,3,4-tri-O-methyl-α-d-glucopyranosyl)-(1→4)-[sodium (2,3-di-O-methyl-β-d-glucopyranosyl) uronate]-(1→4)-(sodium (2,3-di-O-benzyl-6-deoxy-6-C-sulfonatomethyl-α-d-glucopyranosyl)-(1→4)-[methyl (2,3-di-O-methyl-α-l-idopyranosyl)uronate]-(1→4)-(2,3,6-tri-O-benzyl-α-d-glucopyranoside)* (**28**). To a vigorously stirred solution of **27** (40 mg, 0.025 mmol) in CH_2_Cl_2_ (2.0 mL) and H_2_O (1.0 mL), TEMPO (0.8 mg, 0.005 mmol, 0.2 equiv.) and BAIB (32 mg, 0.099 mmol, 4 equiv.) were added. After 24 h, the TLC (9:1 CH_2_Cl_2_/MeOH) indicated moderate conversion of the starting material. Another portion of BAIB (32 mg, 0.099 mmol, 4 equiv.) were added and the stirring was continued for a further 24 h. The reaction mixture was quenched by the addition of 10% aq Na_2_S_2_O_3_ solution (4 mL). The mixture was then extracted twice with EtOAc (8 mL), and the combined organic layers were dried, and concentrated. The residue was purified by column chromatography (9:1 CH_2_Cl_2_/MeOH) to give a colourless syrup (31 mg). The mass spectrum contained peaks corresponding to **28** and its partially debenzylated derivatives; this mixture was used in the subsequent reaction without further purification. MALDI-TOF MS for C_82_H_102_Na_2_O_30_S (1644.60): *m*/*z* 1667.65 [M + Na]^+^ (Calcd. 1667.59); 1379.56 [M + Na (−3Bn)]^+^ (Calcd. 1379.45); 1487.56 [M + Na (−2Bn)]^+^ (Calcd. 1487.49).

*Nona sodium [methyl (2,3,4-tri-O-methyl-6-O-sulfonato-α-d-glucopyranosyl)-(1→4)-[sodium (2,3-di-O-methyl-β-d-glucopyranosyl)uronate]-(1→4)-[sodium (2,3-di-O-sulfonato-6-deoxy-6-C-sulfonatomethyl-α-d-glucopyranosyl)-(1→4)-[sodium (2,3-di-O-methyl-α-l-idopyranosyl)uronate]-(1→4)-2,3,6-tri-O-sulfonato-α-d-glucopyranoside* (**29**). An amount of 0.5 M NaOH solution (0.30 mL) was added to the solution of **28** (30 mg) in a mixture of THF (0.30 mL) and MeOH (0.30 mL) and stirred at room temperature for 24 h. The reaction was quenched by the addition of 1 N HCl solution (1 drop) and the mixture was concentrated. The crude product was converted to sodium salt by ion exchange resin (Dowex, MeOH) to give a colourless syrup (28 mg). The syrupy residue was dissolved in 96% EtOH (3.0 mL) and 10% Pd/C (10 mg) and acetic acid (100 μL) were added. The mixture was stirred at room temperature for 24 h under a 10 bar H_2_ atmosphere. The mixture was diluted with MeOH, the catalyst was filtered off through a pad of Celite and the filtrate was concentrated. The crude product was purified by column chromatography (7:6:1 CH_2_Cl_2_/MeOH/H_2_O, *R*_f_ = 0.13) and gel chromatography (Sephadex G-25, H_2_O) to give the corresponding pentaol (9 mg) which was characterized by MALDI MS. (MALDI-TOF MS for C_39_H_63_Na_3_O_30_S (1112.28): *m*/*z* 1091.39 [M − Na + 2H]^+^ (Calcd. 1091.31), 1113.39 [M + H]^+^ (Calcd. 1113.29), 1135.42 [M + Na]^+^ (Calcd. 1135.27). To the solution of the pentaol derivative (9 mg) in dry DMF (0.6 mL), SO_3_·Et_3_N complex (44 mg, 0.040 mmol) was added and the reaction mixture was stirred at 50 °C for 48 h. The reaction was quenched with satd. aq. NaHCO_3_ (21 mg, 0.24 mmol). The solution was concentrated. The crude product was treated with Dowex ion-exchange resin (Na^+^ form), and then purified by Sephadex G-25 column chromatography eluting with H_2_O to give **29** (7 mg, 17% from **27**) as a white solid. ESI-MS for C_39_H_57_Na_9_O_48_S_7_ (1723.91): *m*/*z* 795.110 [M − 6Na + 4H]^2−^ (Calcd. 795.00); 522.451 [M − 7Na + 4H]^3−^ (Calcd.: 522.34).

*Methyl [2,3,4-tri-O-methyl-6-deoxy-6-C-(ethylsulfonatomethyl)-α-d-glucopyranosyl]-(1→4)-[2,3-di-O-acetyl-β-d-glucopyranosyl]-(1→4)-[2,3-di-O-benzyl-6-deoxy-6-C-(ethylsulfonatomethyl)-α-d-glucopyranosyl]-(1→4)-[methyl (2-O-acetyl-3-O-methyl-α-l-idopyranosyl)uronate]-(1→4)-(2,3,6-tri-O-benzyl-α-d-glucopyranoside)* (**30**). Compound **6** (975 mg, 0.53 mmol) was converted to **30** according to general method **D**. The crude product was purified by column chromatography (94:6 CH_2_Cl_2_/acetone) to give compound **30** (612 mg, 68%) as a colourless syrup; *R*_f_ = 0.26 (93:7 CH_2_Cl_2_/acetone); [α]_d_ +30.56 (*c* 0.10, CHCl_3_); ^1^H-NMR (360 MHz, CDCl_3_) δ 7.45–7.17 (m, 25H, arom.), 5.23 (t, *J* = 9.3 Hz, 1H, H-3-E), 5.11 (s, 1H, H-1-G), 5.00 (d, *J* = 3.6 Hz, 1H, H-1-D), 4.96–4.49 (m, 16H, H-2-E, H-5-G, H-2-G, H-1-E, H-1-H, H-1-F, 10 × PhC*H*_2_), 4.29 (q, *J* = 7.1, 1.2 Hz, 4H, 2 × SO_3_C*H*_2_CH_3_), 3.93–3.07 (m, 21H, skeleton protons), 3.55, 3.52, 3.41, 3.41, 3.34, 3.31 (6s, 18H, 6 × OC*H*_3_), 3.01 (dd, *J* = 9.8, 3.6 Hz, 1H, H-2-D), 2.72 (t, *J* = 9.3 Hz, 1H, H-4-D), 2.34–2.22 (m, 2H, H-6_a_-D, H-6_a_-F), 2.03, 2.01, 1.96 (3s, 9H, 3 × COC*H*_3_), 1.94–1.75 (m, 3H, H-6_b_-D, H-6_b_-F, OH), 1.41 (m, 6H, 2 × SO_3_C*H*_2_CH_3_) ppm; ^13^C-NMR (91 MHz, CDCl_3_) δ 170.2, 170.0, 169.6, 169.6 (4 × *C*O), 139.1, 139.0, 138.1, 138.1, 137.9 (5C, *C*_q_ arom.), 128.6, 128.6, 128.4, 128.4, 128.2, 128.1, 127.9, 127.8, 127.6, 127.3, 127.1, 126.1 (25C, arom.), 100.9, 98.4, 98.0, 97.6, 96.8 (5 × C-1), 83.8, 82.8, 82.1, 81.7, 80.2, 79.8, 79.4, 79.1, 76.1, 75.3, 74.8, 74.2, 72.6, 72.2, 70.2, 69.6, 69.2, 68.4, 67.5 (20C, skeleton carbons), 74.6, 73.9, 73.4, 73.4, 73.4 (5 × Ph*C*H_2_), 68.4, 60.6 (C-6-H, C-6-E), 66.3, 66.1 (2 × SO_3_*C*H_2_CH_3_), 60.8, 60.7, 59.5, 58.2, 55.3, 51.8 (6 × O*C*H_3_), 46.8, 46.6 (C-7-D, C-7-F), 26.4, 25.5 (C-6-D, C-6-F), 21.1, 21.0, 20.6 (3 × COC*H*_3_), 15.2, 15.2 (2 × SO_3_CH_2_*C*H_3_) ppm; MALDI-TOF MS: *m*/*z* 1737.62 [M + Na]^+^ (Calcd. 1737.62); Anal. Calcd. for C_83_H_110_O_34_S_2_ (1714.63): C, 58.10; H, 6.46; O, 31.70; S, 3.74. Found: C, 58.21; H, 6.41; S, 3.70.

*Methyl [2,3,4-tri-O-methyl-6-deoxy-6-C-(ethylsulfonatomethyl)-α-d-glucopyranosyl]-(1→4)-[sodium (2,3-di-O-acetyl-β-d-glucopyranosyl)uronate]-(1→4)-[2,3-di-O-benzyl-6-deoxy-6-C-(ethylsulfonatomethyl)-α-d-glucopyranosyl]-(1→4)-[methyl (2-O-acetyl-3-O-methyl-α-l-idopyranosyl)uronate]-(1→4)-(2,3,6-tri-O-benzyl-α-d-glucopyranoside)* (**31**). Compound **30** (2.4 g, 1.87 mmol) was converted to **31** according to general method **A**. The reaction mixture was stirred for 24 h. The crude product was purified by column chromatography (98:2 CH_2_Cl_2_/MeOH) to give **31** (528 mg, 80%) as a colourless syrup; *R*_f_ = 0.50 (95:5 CH_2_Cl_2_/MeOH); [α]_d_ +29.26 (*c* 0.11, CHCl_3_); ^1^H-NMR (360 MHz, CDCl_3_) δ 7.38–7.19 (m, 25H, arom.), 5.26–5.18 (m, 1H), 5.08 (s, 1H, H-1-G), 5.00 (d, *J* = 3.5 Hz, 1H, H-1-D), 4.96–4.49 (m, 16H, 10 × PhC*H*_2_, skeleton protons), 4.30, 4.28 (2q, 4H, 2 × SO_3_C*H*_2_CH_3_), 4.04 (t, *J* = 8.8 Hz, 1H), 3.93–3.77 (m, 4H, skeleton protons), 3.77–3.60 (m, 4H, skeleton protons), 3.59–3.18 (m, 8H skeleton protons), 3.56, 3.52, 3.42, 3.39, 3.34, 3.30 (6s, 18H, 6 × OC*H*_3_), 3.16–3.05 (m, 1H, H-7_b_), 3.01 (dd, *J* = 9.8, 3.6 Hz, 1H, H-2-D), 2.72 (t, *J* = 9.3 Hz, 1H, H-4-D), 2.33–2.17 (m, 2H, H-6_a_-D, H-6_a_-F), 2.03, 2.01, 1.95 (3s, 9H, 3 × COC*H*_3_), 1.91–1.77 (m, 2H, H-6_b_-D, H-6_b_-F), 1.39, 1.38 (m, 6H, 2 × SO_3_C*H*_2_CH_3_) ppm; ^13^C-NMR (91 MHz, CDCl_3_) δ 170.4, 169.9, 169.5, 169.5, 169.0 (5 × *C*O), 139.1, 138.7, 138.1, 138.1, 137.8 (5C, *C*_q_ arom.), 128.6, 128.5, 128.4, 128.2, 128.2, 128.1, 127.9, 127.8, 127.7, 127.6, 127.5, 127.3, 127.2 (25C, arom.), 100.8, 98.4, 98.0, 97.6, 97.6 (5 × C-1), 83.8, 82.7, 81.9, 81.6, 80.2, 80.1, 79.8, 79.0, 76.2, 75.3, 74.7, 74.4, 74.4, 74.0, 72.4, 70.2, 69.5, 69.1, 68.3, 67.4 (20C, skeleton carbons), 74.9, 74.8, 73.8, 73.4, 73.4 (5 × Ph*C*H_2_), 68.5 (C-6-H), 66.6, 66.4 (2 × SO_3_*C*H_2_CH_3_), 60.7, 60.7, 59.5, 58.2, 55.3, 51.9 (6 × O*C*H_3_), 46.6, 46.5 (C-7-D, C-7-F), 26.0, 25.7 (C-6-D, C-6-F), 21.1, 20.9, 20.6 (3 × COC*H*_3_), 15.2, 15.2 (2 × SO_3_CH_2_*C*H_3_) ppm; MALDI-TOF MS: *m*/*z* 1773.53 [M + Na]^+^ (Calcd. 1773.58); Anal. Calcd. for C_83_H_107_O_35_S_2_ (1714.63): C, 58.10; H, 6.46; O, 31.70; S, 3.74. Found: C, 58.21; H, 6.41; S, 3.70.

*Methyl [2,3,4-tri-O-methyl-6-deoxy-6-C-(sulfonatomethyl)-α-d-glucopyranosyl]-(1→4)-[sodium (β-d-glucopyranosyl)uronate]-(1→4)-[2,3-di-O-benzyl-6-deoxy-6-C-(sulfonatomethyl)-α-d-glucopyranosyl]-(1→4)-[methyl (3-O-methyl-α-l-idopyranosyl)uronate]-(1→4)-(2,3,6-tri-O-benzyl-α-d-glucopyranoside)* (**32**). NaOMe (2 mg, 0.03 mmol) was added to the solution of compound **31** (500 mg, 0.29 mmol) and stirred at room temperature for 24 h. The mixture was quenched by the addition of acetic acid (1–2 drops) and then concentrated. The crude product was dissolved in acetone (20 mL) and NaI (128 mg, 0.86 mmol) was added to the solution. The reaction mixture was stirred at room temperature for 24 h. The mixture was concentrated and the crude product was purified by gel chromatography (Sephadex LH-20, MeOH) to give **32** (386 mg, 84% for two steps) as a colourless syrup. *R*_f_ = 0.47 (7:3 CH_2_Cl_2_/MeOH); [α]_d_ +34.32 (*c* 1.66, CHCl_3_); ^1^H-NMR (360 MHz, CDCl_3_) δ 7.44–7.15 (m, 25H, arom.), 5.51 (d, *J* = 3.5 Hz, 1H), 5.10 (s, 1H), 5.03 (d, *J* = 3.3 Hz, 1H), 5.02–4.50 (m, 14H, 5 × PhC*H*_2_, skeleton protons), 3.99–3.21 (m, 22H, skeleton protons), 3.57, 3.52, 3.52, 3.46, 3.36, 3.35 (6s, 18H, 6 × OC*H*_3_), 3.13 (dd, *J* = 9.8, 3.6 Hz, 1H, H-2-D), 3.08–2.95 (m, 2H, H-7_a_-D, H-7_a_-F), 2.84–2.75 (m, 1H, H-4-D), 2.43–2.31 (m, 1H, H-6_a_), 2.30–2.18 (m, 1H, H-6_a_), 2.11–1.78 (m, 2H, 2 × H-6_b_) ppm; ^13^C-NMR (91 MHz, CDCl_3_) δ 177.8, 171.6 (2 × *C*O), 140.2, 140.1, 139.5, 139.4, 139.0 (5C, *C*_q_ arom.), 129.6, 129.3, 129.2, 129.1, 128.9, 128.7, 128.4 (25C, arom.), 104.3, 102.3, 98.9, 98.2, 95.9 (5 × C-1), 85.1, 83.7, 81.8, 81.2, 80.8, 80.4, 79.3, 78.4, 78.2, 76.2, 75.9, 75.7, 75.2, 73.6, 72.5, 71.7, 70.9, 70.7, 69.1, 67.8 (20C, skeleton carbons), 76.3, 76.1, 75.0, 74.5, 74.0 (5 × Ph*C*H_2_), 69.8 (C-6-H), 60.8, 60.6, 59.3, 59.2, 58.5, 55.5 (6 × O*C*H_3_), 28.3, 27.8 (C-6-D, C-6-F) ppm; MALDI-TOF MS: *m*/*z* 1635.50 [M + Na]^+^ (Calcd. 1635.45); Anal. Calcd. for C_73_H_91_Na_3_O_32_S_2_ (1612.46): C, 54.34; H, 5.68; Na, 4.27; O, 31.73; S, 3.97. Found: C, 54.23; H, 5.66; S, 4.03.

*Methyl-[sodium 2,3,4-tri-O-methyl-6-deoxy-6-C-(sulfonatomethyl)-α-d-glucopyranosyl]-(1→4)-[sodium (2,3-di-O-methyl-β-d-glucopyranosyl)uronate]-(1→4)-[2,3-di-O-benzyl-6-deoxy-6-C-(sulfonatomethyl)-α-d-glucopyranosyl]-(1→4)-[methyl (2,3-di-O-methyl-α-l-idopyranosyl)uronate]-(1→4)-2,3,6-tri-O-benzyl-α-d-glucopyranoside* (**33**). An amount of 60 m/m% NaH (55 mg, 1.38 mmol) was added to the solution of compound **32** (370 mg, 0.23 mmol) in dry DMF (40 mL) at 0 °C. After 30 min of stirring at room temperature, MeI (64 μL, 1.04 mmol) was added to the reaction mixture and it was stirred for 4 h. The reaction mixture was quenched by the addition of MeOH and acetic acid (1–2 drops). The solution was concentrated and the crude product was purified by gel chromatography (Sephadex LH-20, MeOH) to give **33** (249 mg, 65%) as a colourless syrup; *R*_f_ = 0.53 (7:3 CH_2_Cl_2_/MeOH); [α]_d_ +4.09 (*c* 0.81, CHCl_3_); ^1^H-NMR (360 MHz, CDCl_3_) δ 7.49–7.10 (m, 25H, arom.), 5.15–4.51 (m, 17H, 5 × H-1, H-5-E, H-5-G, 10 × PhC*H*_2_), 4.00–3.24 (m, 20H, skeleton protons), 3.57, 3.55, 3.53, 3.49, 3.46, 3.43, 3.38, 3.36, 3.35 (9s, 27H, 9 × OC*H*_3_), 3.11 (dd, *J* = 9.7, 3.6 Hz, 1H, H-2-D), 3.09–2.77 (m, 2H, 2 × H-7_b_), 2.56–2.38 (m, 1H, H-6_a_), 2.30–2.17 (m, 1H, H-6_a_), 2.07–1.85 (m, 2H, 2 × H-6_b_) ppm; ^13^C-NMR (101 MHz, MeOD) δ 170.4, 170.0 (2 × *C*O), 139.3, 139.3, 138.5, 138.4, 138.4 (5C, *C*_q_ arom.), 128.8, 128.7, 128.6, 128.5, 128.3, 128.3, 128.2, 128.1, 128.0, 127.9, 127.6 (25C, arom.), 100.1, 100.0, 98.4, 96.7, 96.0 (5 × C-1), 86.2, 84.2, 83.8, 83.5, 82.7, 82.1, 81.7, 81.4, 80.3, 79.5, 79.2, 78.9, 76.5, 74.7, 74.3, 71.6, 71.4, 70.7, 70.1, 68.8 (20C, skeleton carbons), 75.7, 75.5, 75.2,73.8, 73.8 (5 × Ph*C*H_2_), 73.9 (C-6-H), 60.9, 60.9, 60.1, 60.0, 59.8, 59.3, 55.5, 53.0, 52.3, (9 × CO*C*H_3_), 47.6, 47.5 (C-7-D, C-7-F), 27.5, 27.3 (C-6-D, C-6-F) ppm; MALDI-TOF MS: *m*/*z* 1655.53 [M + Na]^+^ (Calcd. 1655.52); Anal. Calcd. for C_76_H_97_Na_3_O_32_S_2_ (1654.51): C, 55.13; H, 5.91; Na, 4.17; O, 30.92; S, 3.87. Found: C, 55.20; H, 5.97; S, 3.79.

*Methyl [2,3,4-tri-O-methyl-6-deoxy-6-C-sulfonatomethyl-α-d-glucopyranosyl]-(1→4)-[sodium (2,3-di-O-methyl-β-d-glucopyranosyl)uronate]-(1→4)-[2,3-di-O-benzyl-6-deoxy-6-C-(sulfonatomethyl)-α-d-glucopyranosyl]-(1→4)-[sodium (2,3-di-O-methyl-α-l-idopyranosyl)uronate]-(1→4)-(2,3,6-tri-O-benzyl-α-d-glucopyranoside)* (**34**). An amount of 0.5 M NaOH solution (2 mL) was added to the solution of compound **33** (206 mg, 0.12 mmol) in a mixture of THF (2 mL) and MeOH (2 mL) and stirred at room temperature for 24 h. The reaction was quenched by the addition of 1 N HCl solution (1–2 drops) and the mixture was concentrated. The crude product was converted to sodium salt by ion exchange resin (Dowex, MeOH) to give **34** (187 mg, 90%) as a colourless syrup; *R*_f_ = 0.24 (8:2 CH_2_Cl_2_/MeOH); [α]_d_ +42.81 (*c* 0.10, CHCl_3_); ^1^H-NMR (360 MHz, CDCl_3_) δ 7.43–7.20 (m, 25H, arom.), 5.46 (d, *J* = 3.6 Hz, 1H), 5.15 (d, *J* = 3.3 Hz, 1H), 5.10 (d, *J* = 3.9 Hz, 1H), 5.04–4.48 (m, 14H, 2 × H-1, H-5-E, H-5-G, 10 × PhC*H*_2_), 4.09–3.25 (m, 17H, skeleton protons), 3.58, 3.54, 3.53, 3.52, 3.50, 3.43, 3.35, 3.34 (8s, 24H, 8 × OC*H*_3_), 3.23–3.16 (m, 1H), 3.09 (dd, 1H, H-2-D), 3.07–3.86 (m, 2H, H-7_b_-D, H-7_b_-F), 2.81 (t, *J* = 9.8 Hz, 1H, H-4-D), 2.60–2.48 (m, 1H, H-6_a_), 2.33–2.19 (m, 1H, H-6_a_), 1.98–1.82 (m, 2H, H-6_b_-D, H-6_b_-F) ppm; ^13^C-NMR (101 MHz, MeOD) δ 171.1, 170.7 (2 × *C*O), 140.4, 140.2, 139.6, 139.5, 139.5 (5C, *C*_q_ arom.), 129.4, 129.3, 129.2, 129.0, 129.0, 128.8, 128.7, 128.6, 128.4, 128.2, 128.0 (25C, arom.), 104.6, 100.5, 98.9, 96.8, 96.7 (5 × C-1), 87.0, 85.4, 84.5, 84.1, 82.9, 82.8, 81.3, 81.2, 80.8, 80.6, 80.4, 76.6, 75.2, 74.9, 74.1, 71.6, 71.5, 71.0, 70.7 (20C, skeleton carbons), 76.0, 75.8, 74.5, 73.9, 73.8 (5 × Ph*C*H_2_), 69.6 (C-6-H), 61.2, 60.9, 60.6, 59.9, 59.5, 55.9, 55.9, 55.6 (8 × O*C*H_3_), 30.7, 28.3 (C-6-D, C-6-F) ppm; MALDI-TOF MS: *m*/*z* 1685.49 [M + Na]^+^ (Calcd. 1685.47); Anal. Calcd. for C_75_H_94_Na_4_O_32_S_2_ (1662.48): C, 54.15; H, 5.70; Na, 5.53; O, 30.78; S, 3.85. Found: C, 54.08; H, 5.67; S, 3.87.

*Methyl [2,3,4-tri-O-methyl-6-deoxy-6-C-sulfonatomethyl-α-d-glucopyranosyl]-(1→4)-[sodium (2,3-di-O-methyl-β-d-glucopyranosyl)uronate]-(1→4)-[6-deoxy-6-C-sulfonatomethyl-α-d-glucopyranosyl]-(1→4)-[sodium (2,3-di-O-methyl-α-l-idopyranosyl)uronate]-(1→4)-α-d-glucopyranoside* (**35**). An amount of 10% Pd/C (110 mg) and acetic acid (350 μL) were added to the solution of compound **34** (180 mg, 0.11 mmol) in 96 *v*/*v* % EtOH (10 mL). The mixture was stirred at room temperature for 24 h under a 10 bar H_2_ atmosphere. The mixture was diluted with MeOH and the catalyst was filtered off on Celite-pad. The filtrate was concentrated. The crude product was purified by column chromatography (7:6:1 CH_2_Cl_2_/MeOH/H_2_O) and gel chromatography (Sephadex G-25, H_2_O) to give **35** (123 mg, 92%) as a colourless syrup; *R*_f_ = 0.25 (7:6:1 CH_2_Cl_2_/MeOH/H_2_O); [α]_d_ +21.82 (*c* 0.21, CHCl_3_); ^1^H-NMR (360 MHz, CDCl_3_) δ 5.54 (s, 1H), 5.14 (s, 1H), 5.09 (s, 1H), 4.83 (s, 2H), 4.62 (s, 1H), 4.18 (s, 1H), 3.97–3.25 (m, 46H, skeleton protons, 8 × OC*H*_3_), 3.18–2.96 (m, 5H), 2.90 (d, *J* = 2.7 Hz, 1H), 2.49–2.35 (m, 1H, H-6_a_), 2.29–2.16 (m, 1H, H-6_a_), 1.99–1.85 (m, 2H, H-6_b_, H-6_b_) ppm; ^13^C-NMR (91 MHz, CDCl_3_) δ 193.3, 193.2 (2 × *C*O), 103.5, 103.4, 100.1, 100.0, 96.2 (5 × C-1), 86.8, 84.4, 83.9, 83.5, 82.3, 81.8, 80.4, 78.6, 78.1, 76.7, 73.9, 72.8, 72.6, 72.5, 72.4, 71.9, 71.6, 71.4, 70.3, 69.9 (20C, skeleton carbons), 61.2 (C-6-H), 61.5, 61.1, 60.4, 60.4, 59.9, 59.6, 58.9, 55.9 (8 × OC*H*_3_), 48.3, 48.2 (C-7-D, C-7-F), 27.3, 27.0 (C-6-D, C-6-F) ppm; ESI-MS: *m*/*z* 561.46 [M + 2H]^2−^ (Calcd. 561.15); Anal. Calcd. for C_40_H_64_Na_4_O_32_S_2_ (1212.24): C, 39.61; H, 5.32; Na, 7.58; O, 42.21; S, 5.29. Found: C, 39.56; H, 5.29; S, 5.24.

*Nona sodium [methyl (2,3,4-tri-O-methyl-6-deoxy-6-C-sulfonatomethyl-α-d-glucopyranosyl)]-(1→4)-[2,3-di-O-methyl-β-d-glucopyranosyluronate]-(1→4)-[2,3-di-O-sulfonato-6-deoxy-6-C-sulfonatomethyl-α-d-glucopyranosyl]-(1→4)-[2,3-di-O-methyl-α-l-idopyranosyluronate]-(1→4)-2,3,6-tri-O-sulfonato-α-d-glucopyranoside* (**36**). To the solution of compound **35** (58 mg, 0.047 mmol) in dry DMF (4 mL) sulfur trioxide–triethylamine complex (215 mg, 1.187 mmol) was added and the reaction mixture was stirred at 50 °C for 72 h. The reaction was quenched with satd. aq. NaHCO_3_ (262 mg, 3.12 mmol). The solution was concentrated. The crude product was treated with Dowex ion-exchange resin (Na^+^ form), and then purified by Sephadex G-25 column chromatography eluting with H_2_O to give **36** (28 mg, 36%) as a white powder. *R*_f_ = 0.53 (7:4:1 CH_2_Cl_2_/MeOH/H_2_O); ESI-MS: *m*/*z* for C_40_H_59_Na_9_O_47_S_7_ (1721.94): 837.771 [M − 2Na]^2−^ (Calcd. 837.979); 794.136 [M − 6Na + 4H]^2−^ (Calcd. 794.015).

## 4. Conclusions

Five new idraparinux-analogue pentasaccharide precursors bearing one or two primary sulfonatomethyl moieties at the **D**, **F** or **H** glucose units have been prepared using four disaccharides and two monosaccharides as the building blocks. The synthetic approach, including two subsequent glycosylation steps proved to be highly efficient, and the glycosylation reactions proceeded in good to excellent yields with complete stereoselectivity, regardless of the *C*-sulfonation pattern of the building blocks.

Unexpectedly, the transformation of the protected pentasaccharides into the fully *O*-sulfated and *O*-methylated end-products was troublesome. Upon synthesis of pentasaccharide **29** from **9** via a Zemplén deacetylation, *O*-methylation, NAP-deprotection and TEMPO-BAIB oxidation route, the glucuronide formation proceeded with low efficacy. Fortunately, pentasaccharide **6** could be converted into the desired disulfonic acid product in an acceptable yield by applying a reaction sequence in which the oxidative formation of the glucuronic acid residue preceded the introduction of the methyl ether groups. A study to improve the yields of the synthetic procedures at a pentasaccharide level is in progress in our laboratory.

Synthesis of further isosteric sulfonic acid analogues of idraparinux and evaluation of their anticoagulant activity will be reported in due course.

## Figures and Tables

**Table:** 

Compound	Anti-Xa Activity (U/mg)
Arixtra (**2**)	1195 ± 189
Idraparinux (**3**)	1911 ± 193
Pentasaccharide disulfonic acid **4**	2153 ± 153
Pentasaccharide trisulfonic acid **5**	384 ± 139

## Supplementary Materials

Supplementary materials can be accessed at: http://www.mdpi.com/1420-3049/21/11/1497/s1. copies of ^1^H-NMR and ^13^C-NMR spectra of described compounds.
