# Supplementary material for: A Modular Synthetic Approach to Isosteric Sulfonic Acid Analogues of the Anticoagulant Pentasaccharide Idraparinux
Source: Molecules. 2016 Nov 11;21(11):1497. doi: 10.3390/molecules21111497 (PMC6274384; doi:10.3390/molecules21111497)
Supplement: Supplementary file 1 [file molecules-21-01497-s001.pdf]

Supplementary Materials: A Modular Synthetic Approach to Isosteric Sulfonic Acid Analogues of the Anticoagulant Pentasaccharide Idraparinux

Erika Mező, Dániel Eszenyi, Eszter Varga, Mihály Herczeg and Anikó Borbás

^{1}H- and ^{13}C-NMR spectra of compounds **12**, **17**, **18**, **21**, **6**, **7**, **8**, **9**, **10**, **25**, **26**, **27**, **30**, **31**, **32**, **33**, **34** and **35**.

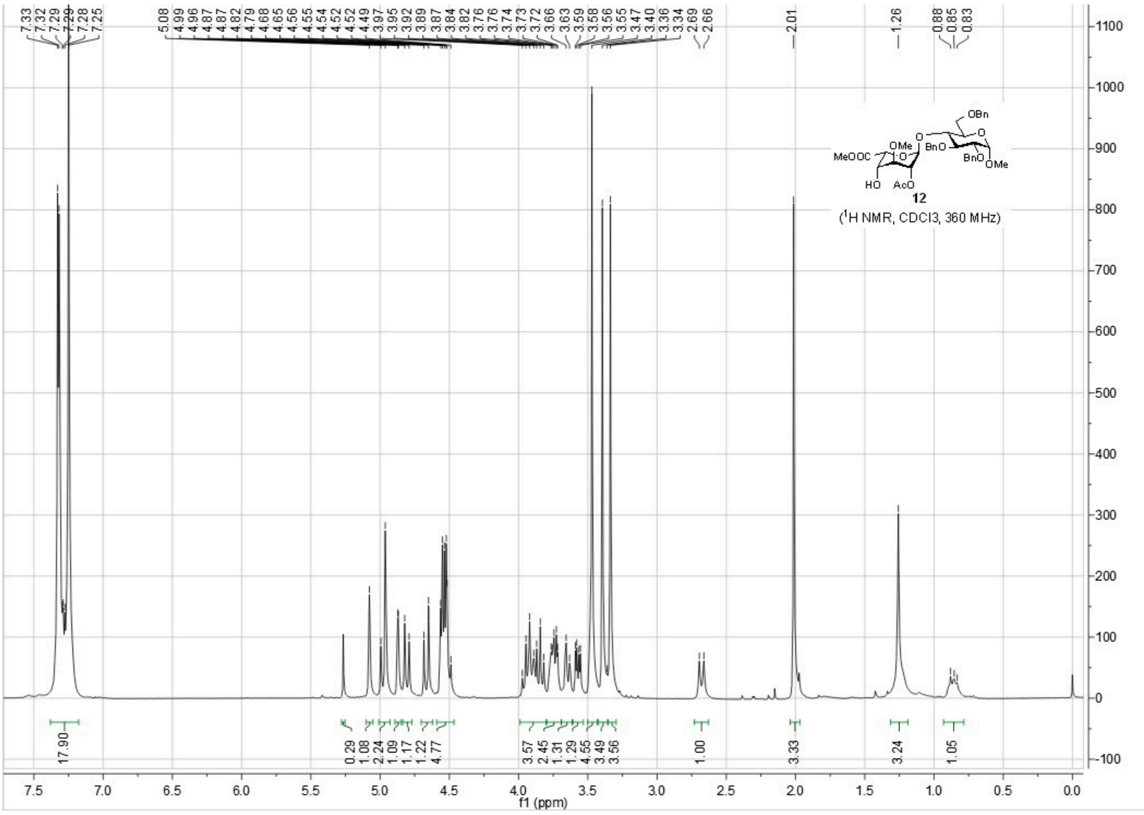

Figure S1. ^{1}H-NMR spectrum of compound **12** in $CDCl_3$.

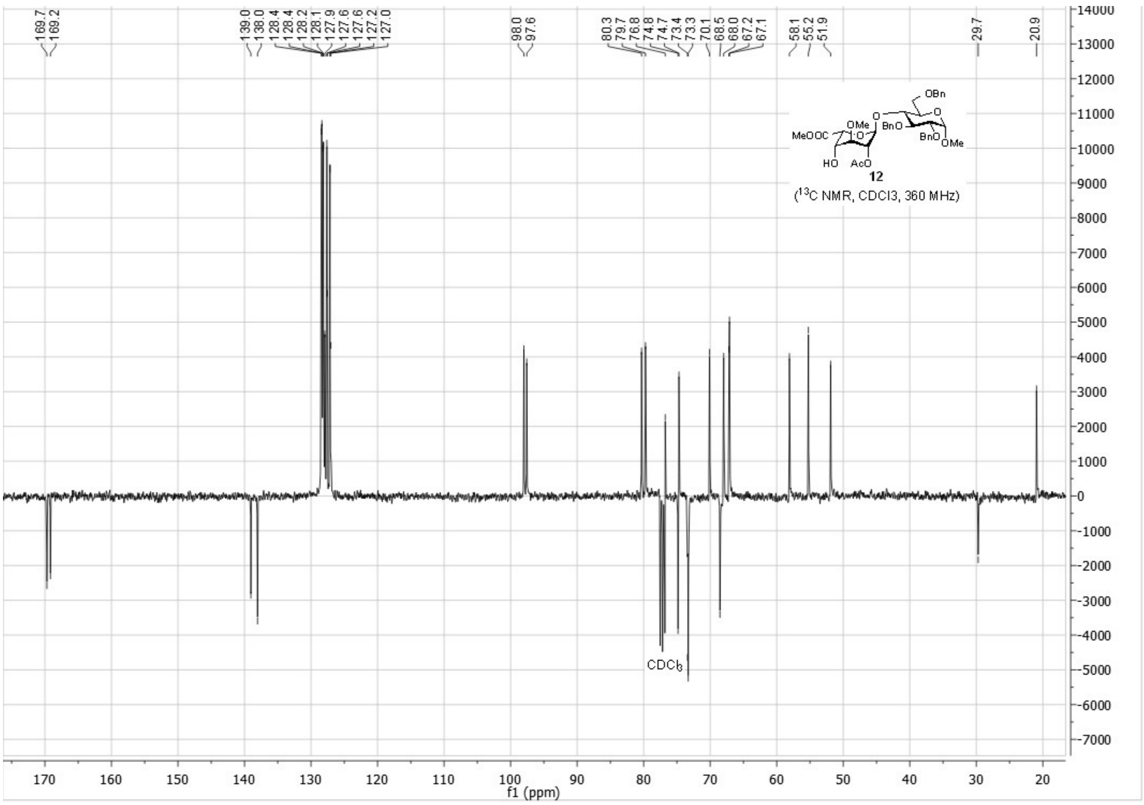

Figure S2. ^{13}C-NMR spectrum of compound **12** in $CDCl_3$.

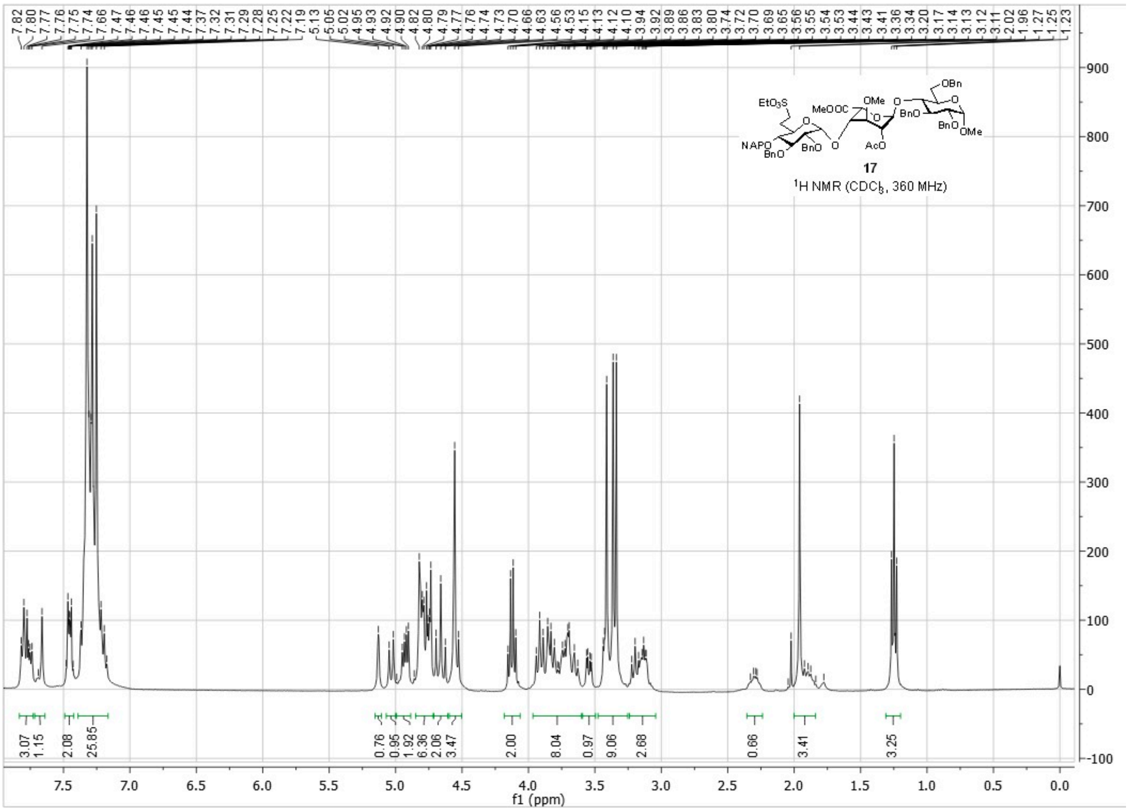

Figure S3. ^{1}H-NMR spectrum of compound **17** in $CDCl_3$.

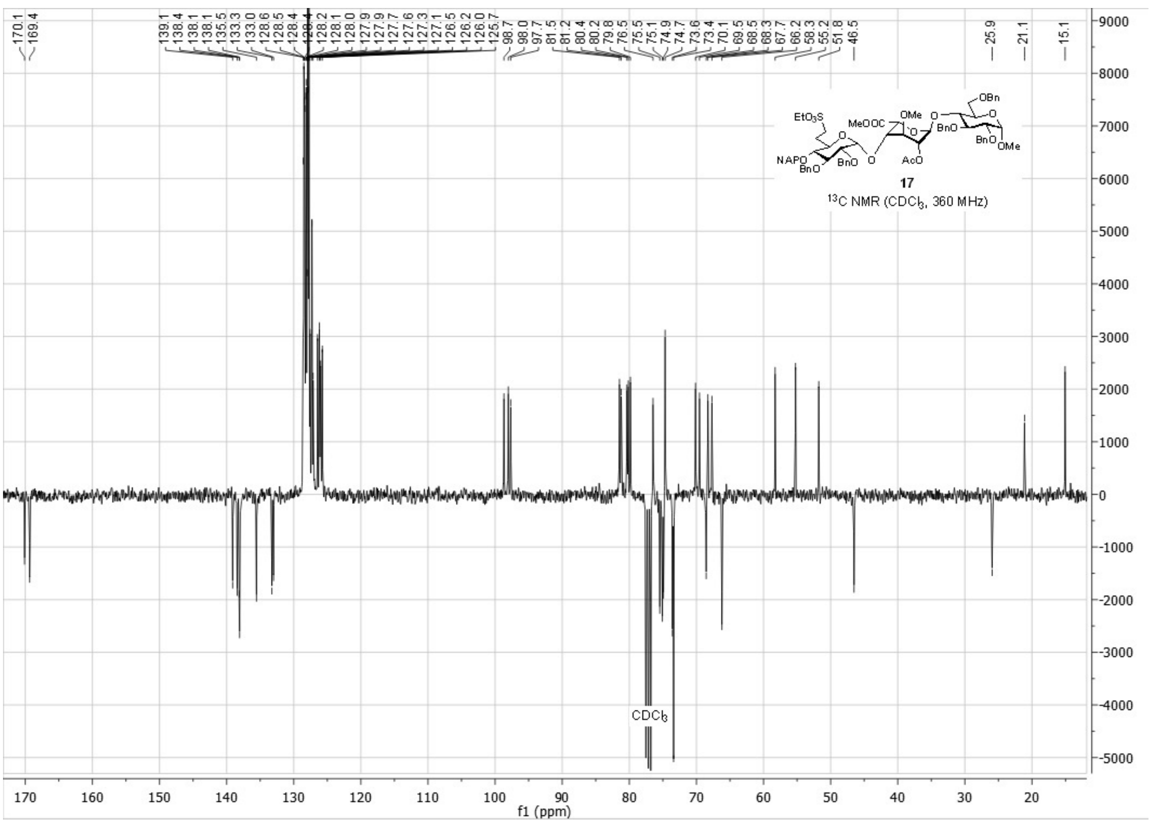

Figure S4. ^{13}C-NMR spectrum of compound **17** in $CDCl_3$.

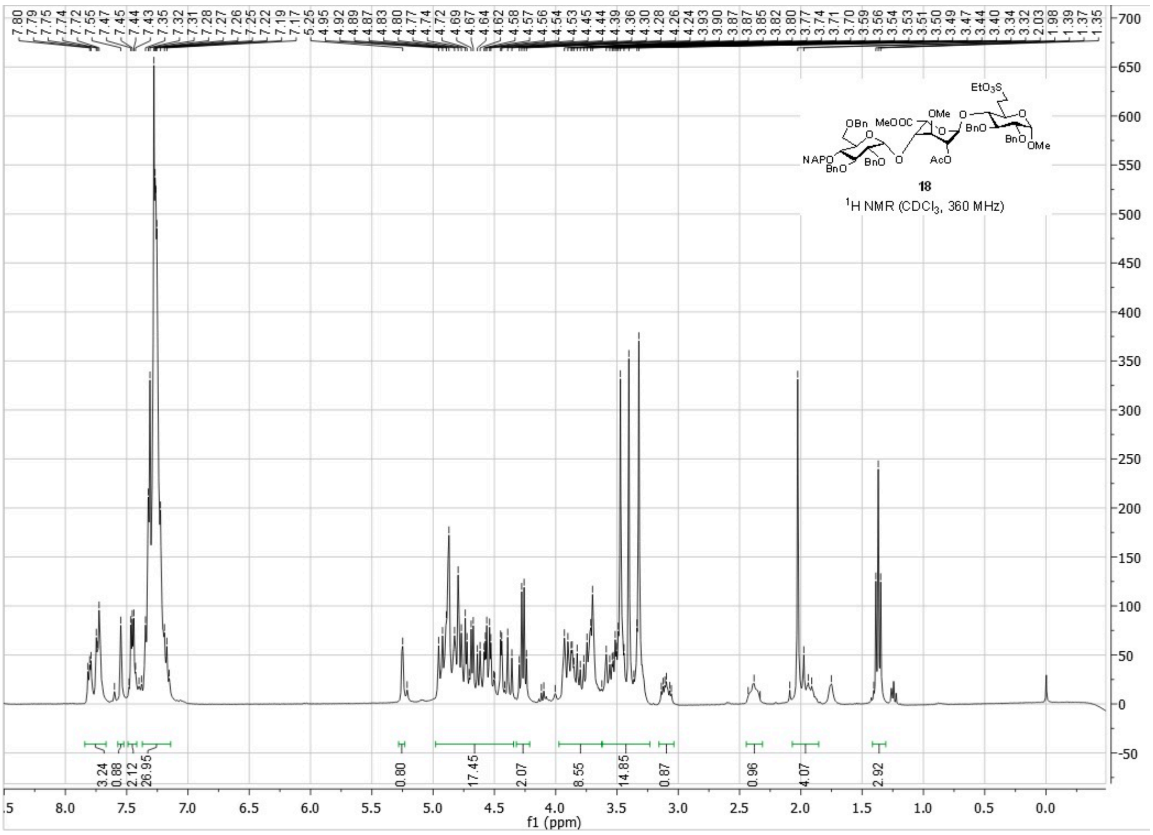

Figure S5. ^{1}H-NMR spectrum of compound **18** in $CDCl_3$.

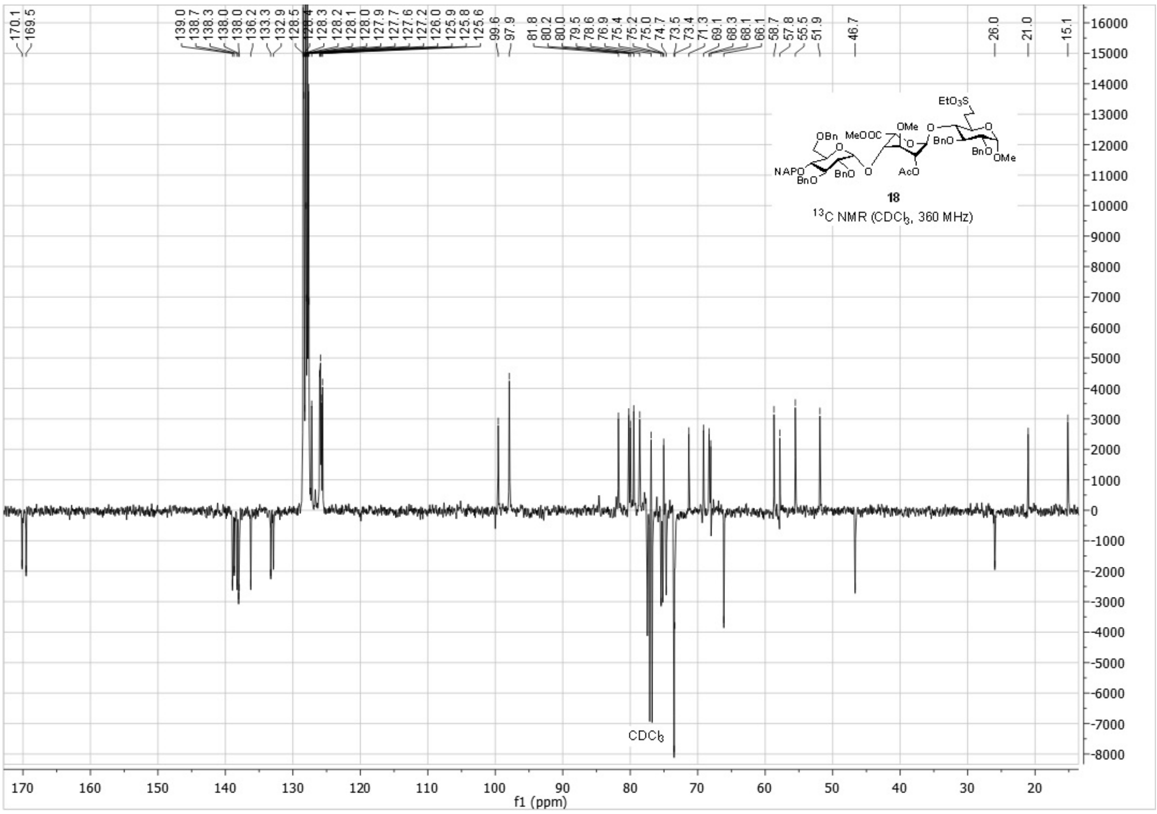

Figure S6. ^{13}C-NMR spectrum of compound **18** in $CDCl_3$.

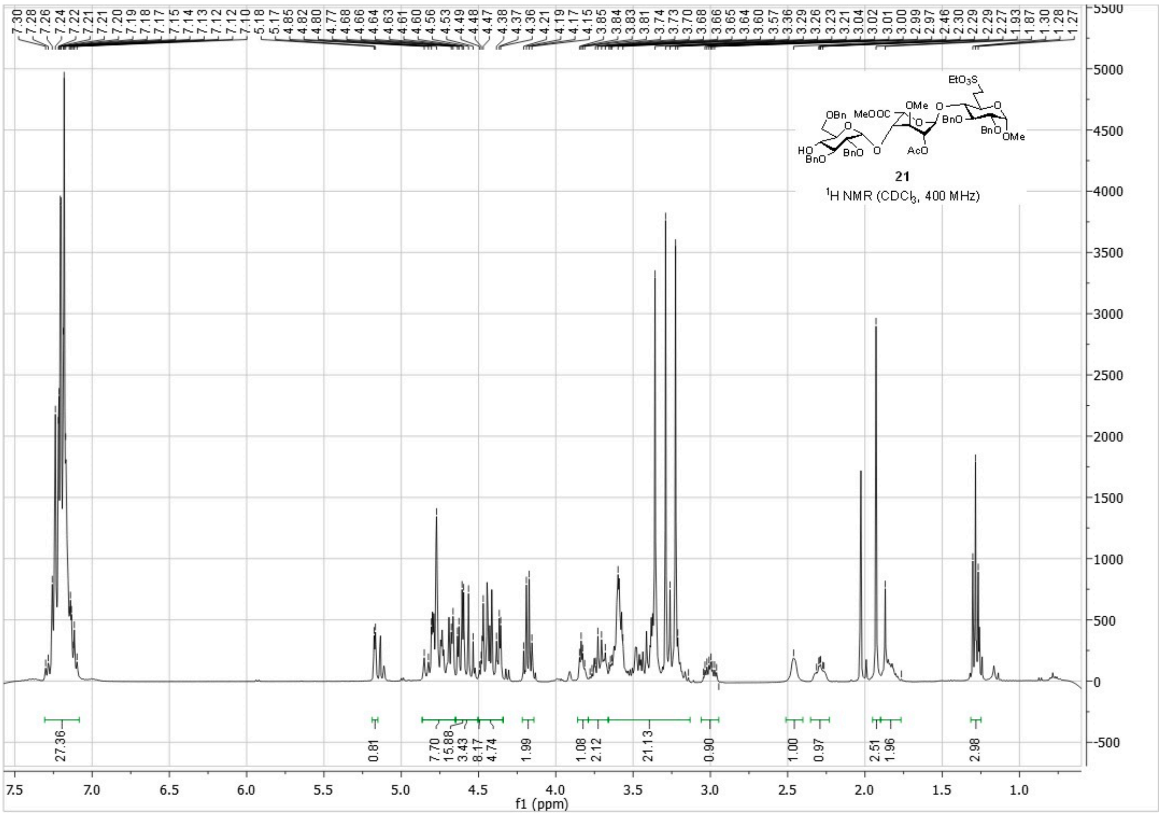

Figure S7. ^{1}H-NMR spectrum of compound **21** in $CDCl_3$.

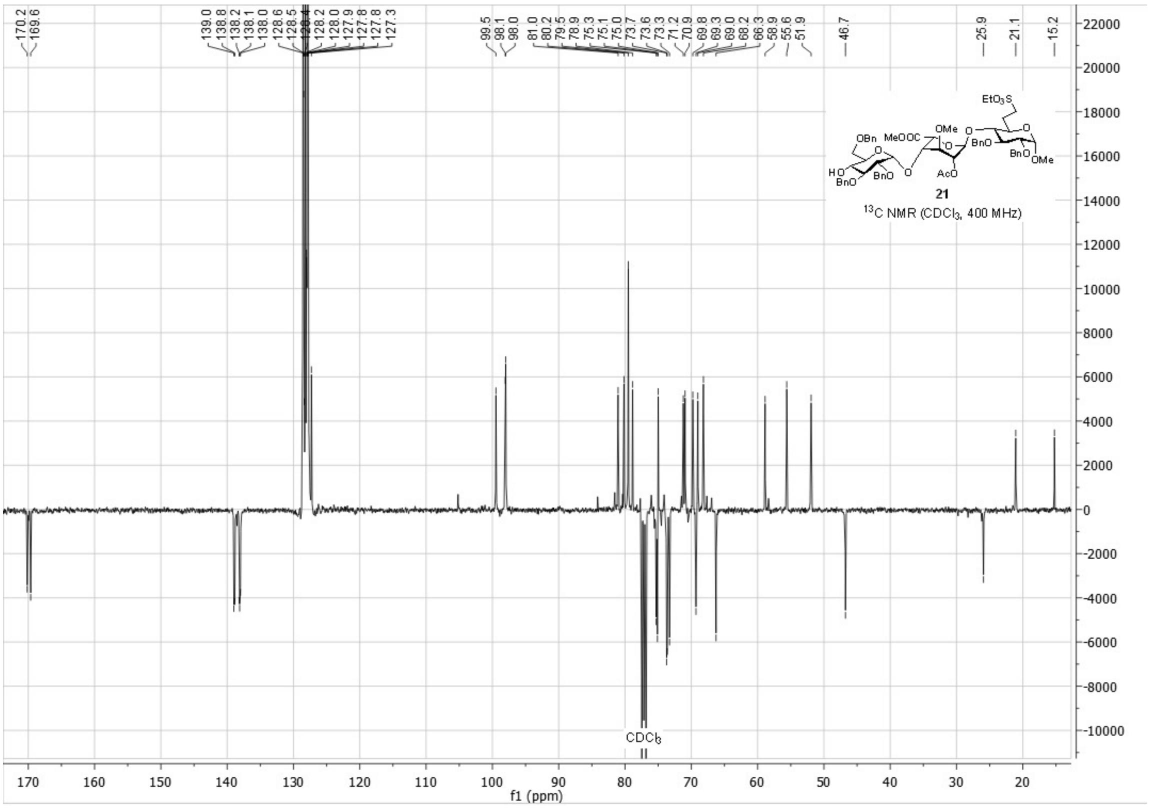

Figure S8. ^{13}C-NMR spectrum of compound **21** in $CDCl_3$.

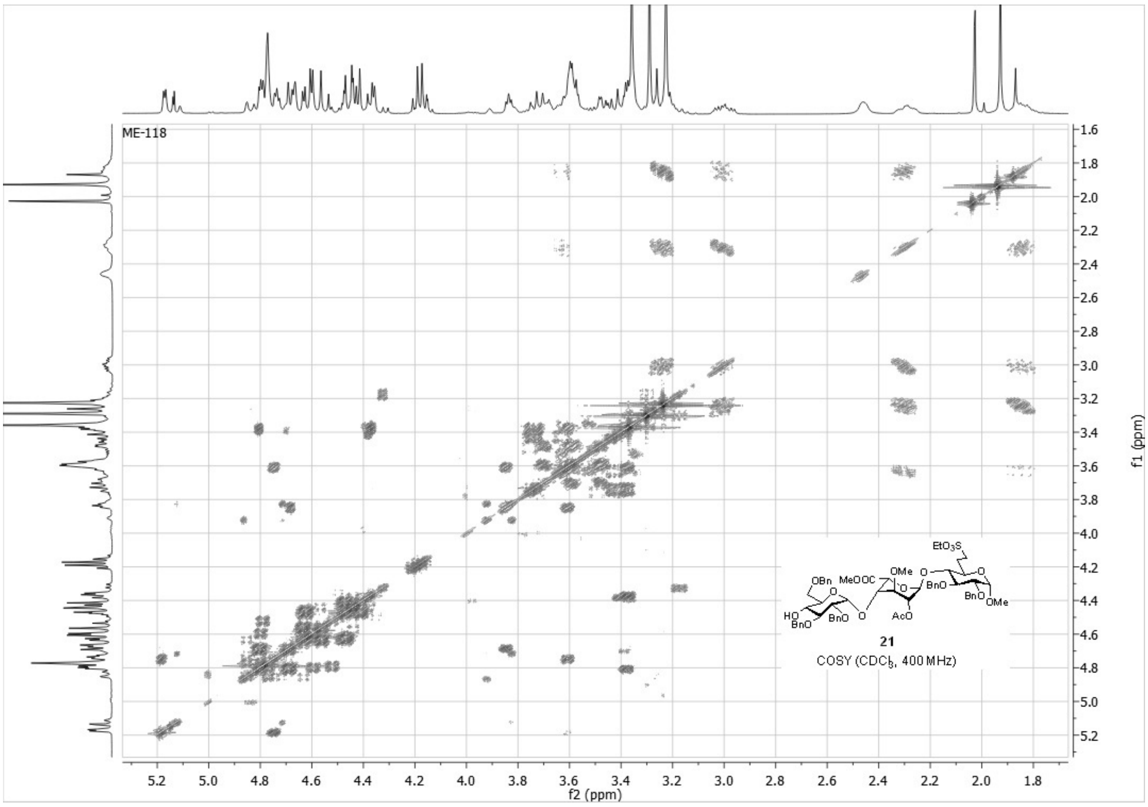

Figure S9. ^{1}H–^{1}H COSY (homonuclear correlation spectroscopy) spectrum of compound **21** in $CDCl_3$.

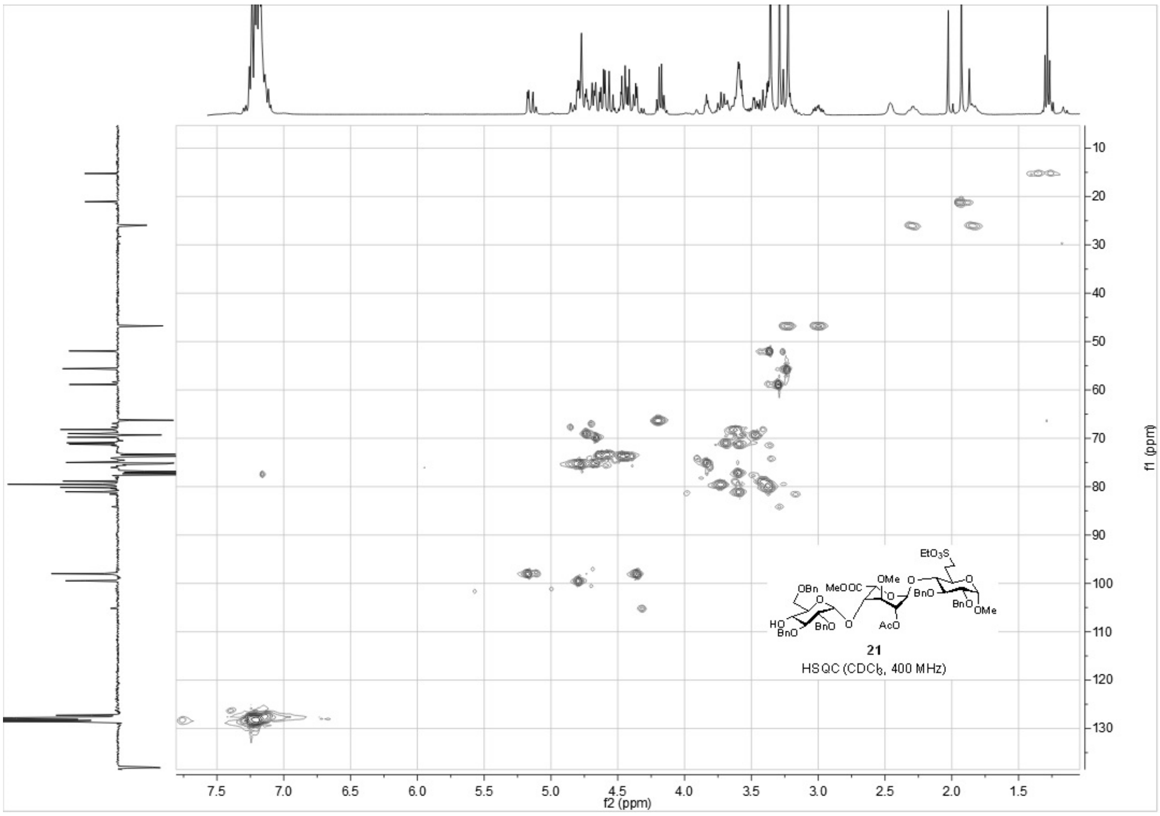

Figure S10. ^{1}H–^{13}C HSQC (heteronuclear single-quantum correlation spectroscopy) spectrum of compound **21** in $CDCl_3$.

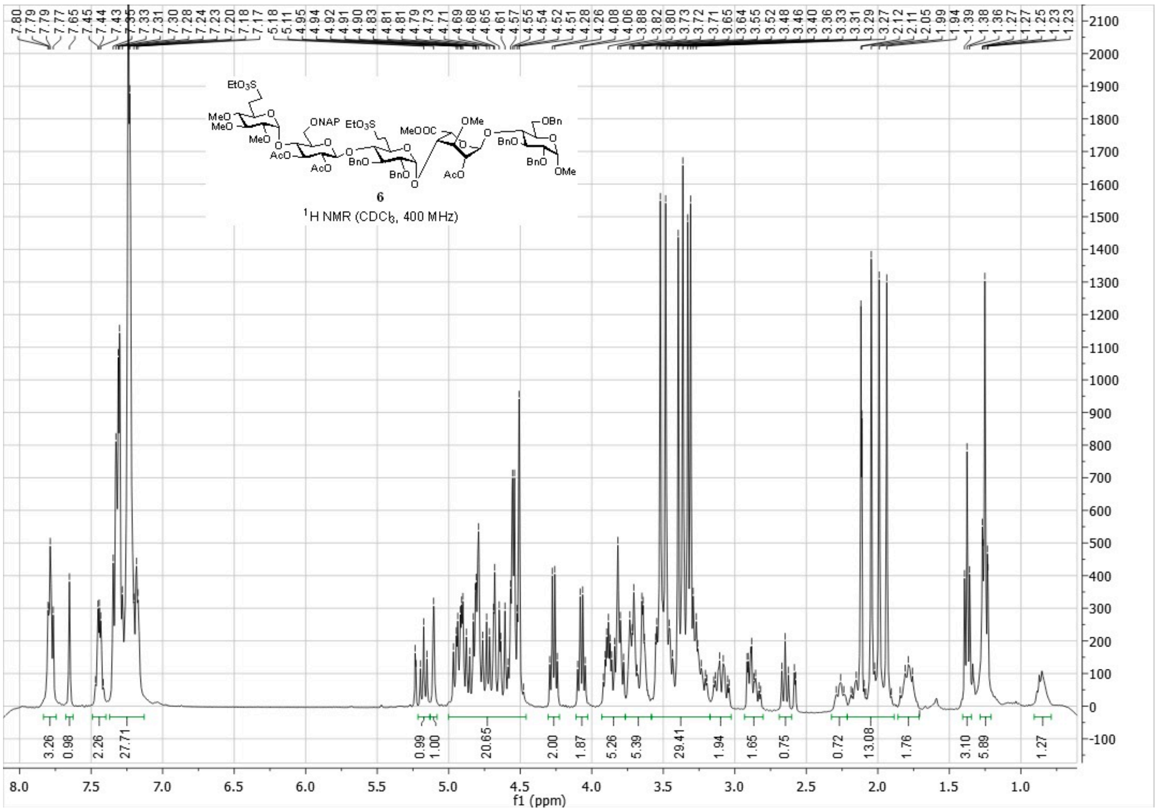

Figure S11. ^{1}H-NMR spectrum of compound **6** in $CDCl_3$.

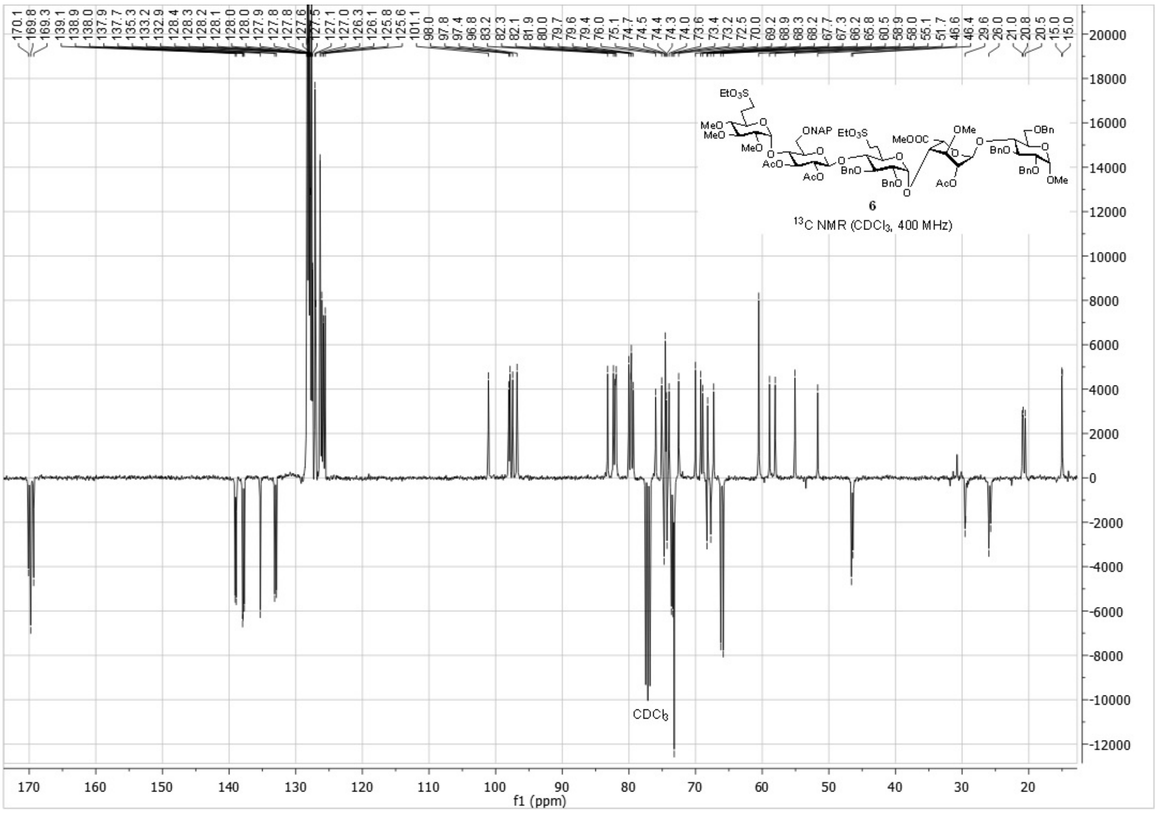

Figure S12. ^{13}C-NMR spectrum of compound **6** in $CDCl_3$.

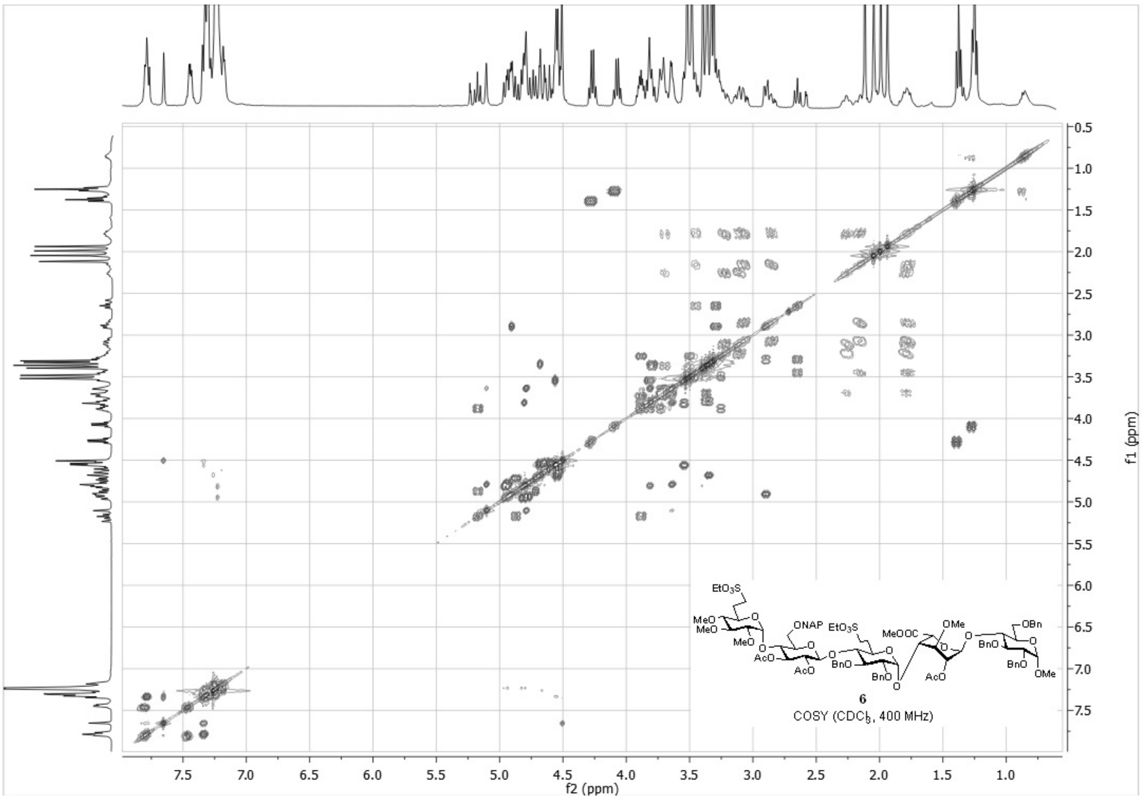

Figure S13. ^{1}H–^{1}H COSY spectrum of compound **6** in $CDCl_3$.

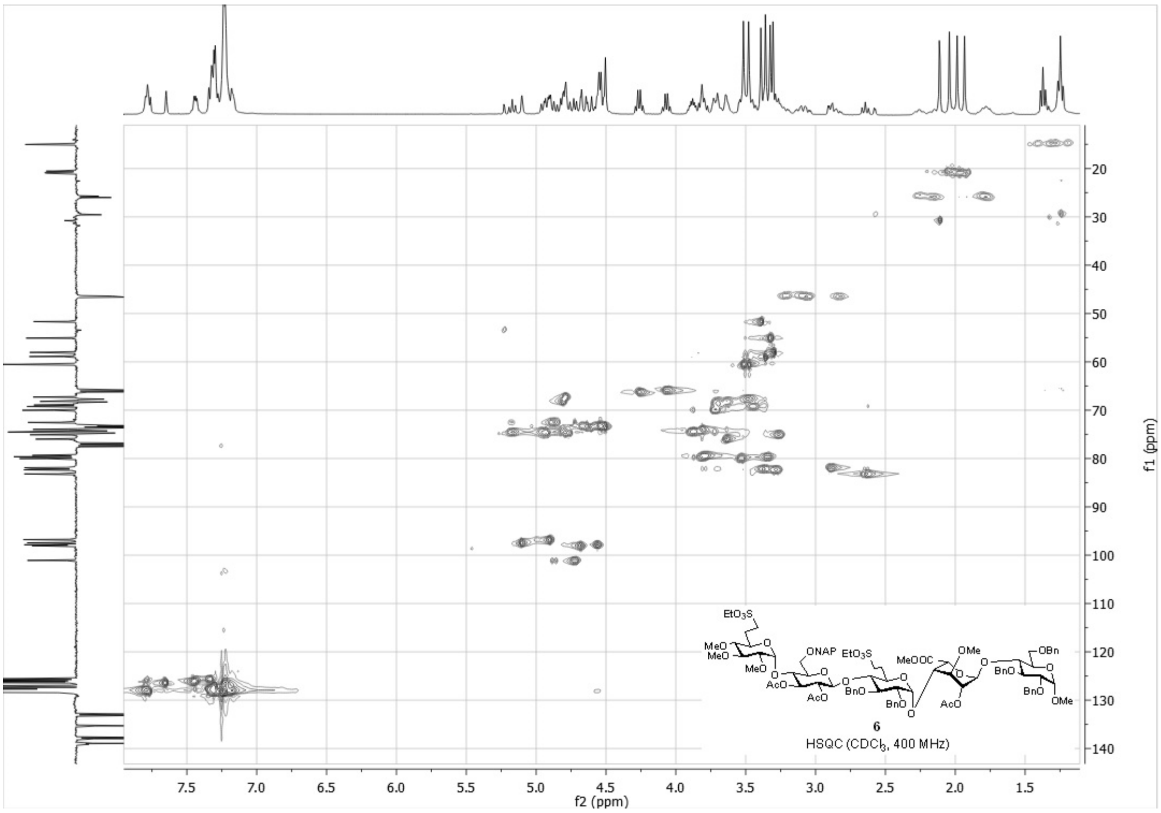

Figure S14. ^{1}H–^{13}C HSQC spectrum of compound **6** in $CDCl_3$.

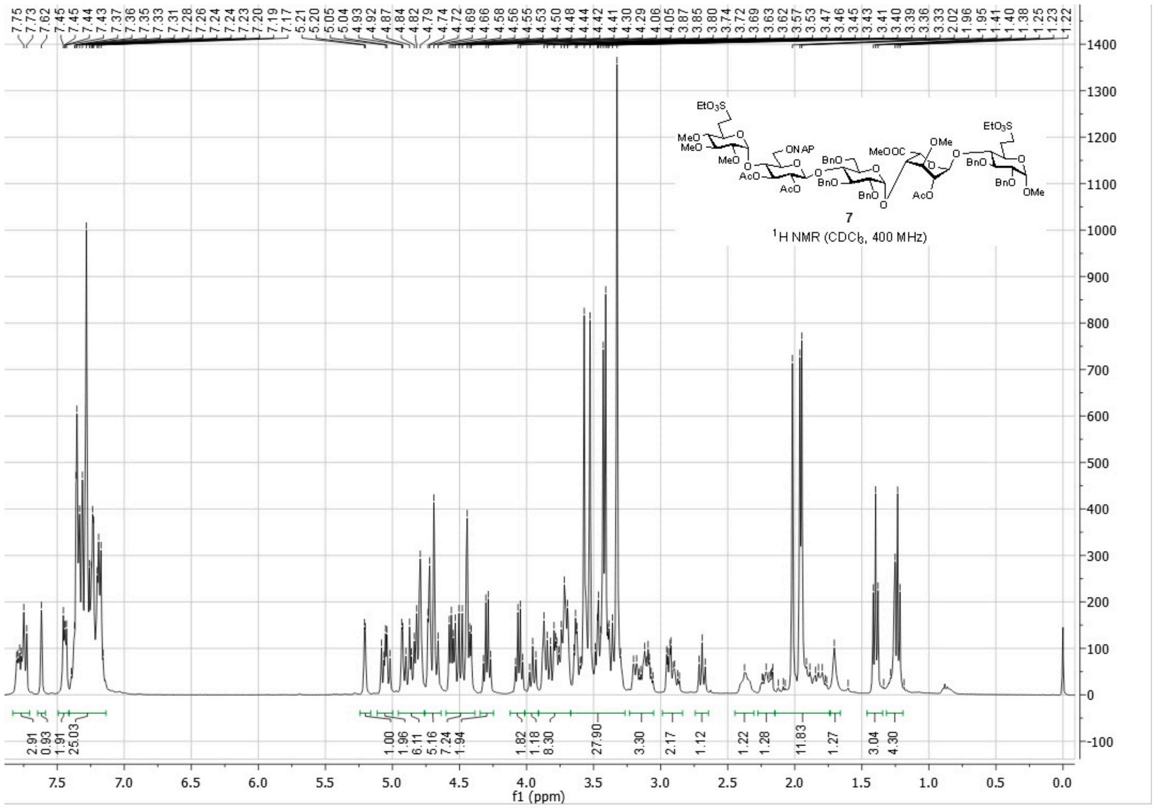

Figure S15. ^{1}H-NMR spectrum of compound **7** in $CDCl_3$.

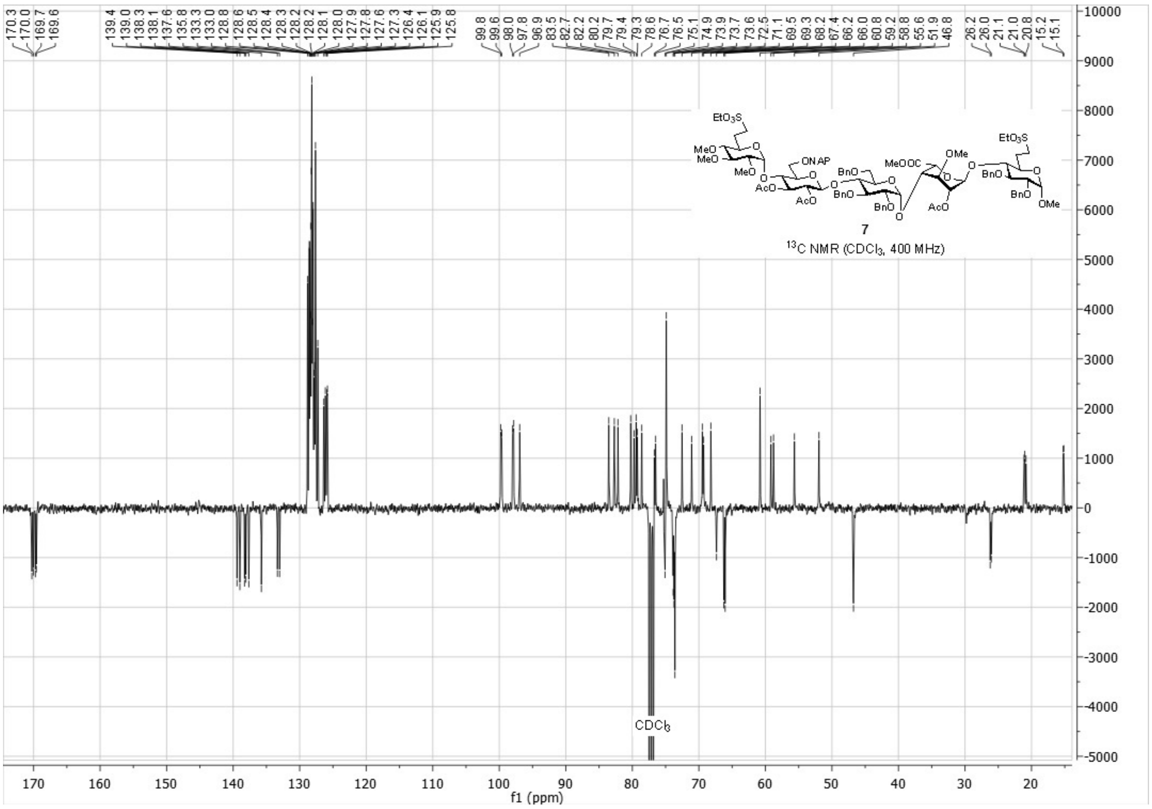

Figure S16. ^{13}C-NMR spectrum of compound **7** in $CDCl_3$.

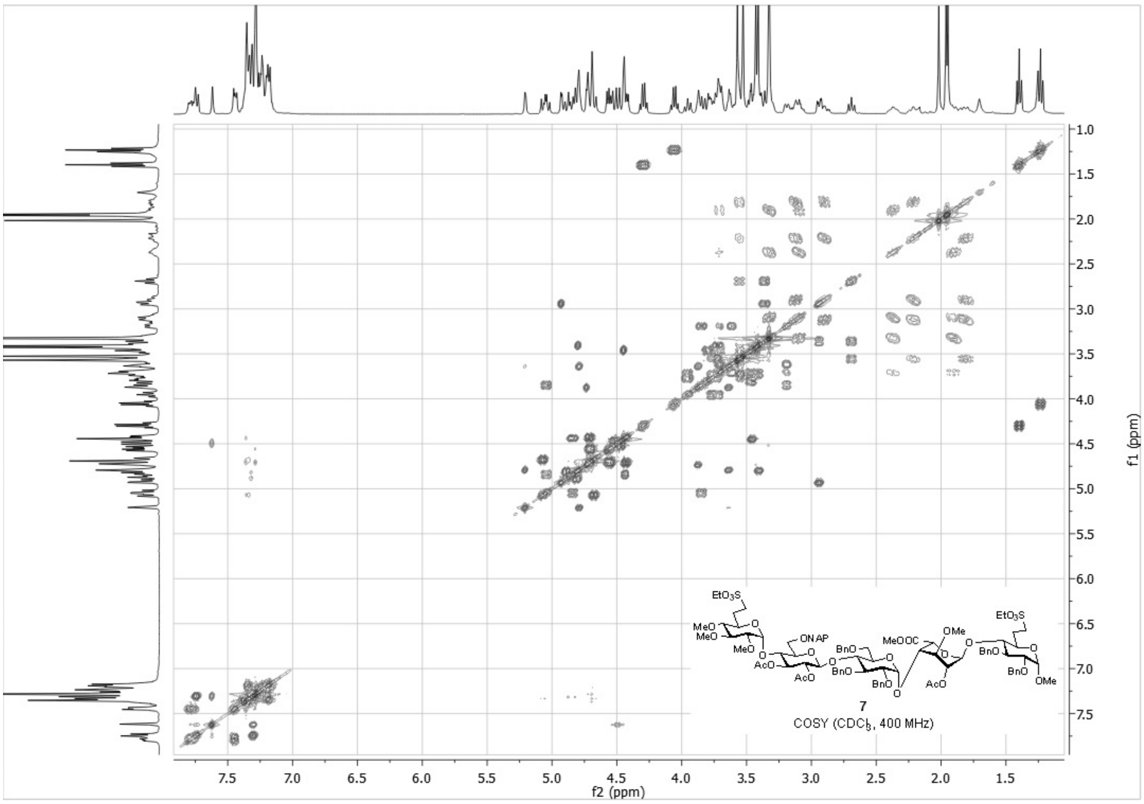

Figure S17. ^{1}H–^{1}H COSY spectrum of compound **7** in $CDCl_3$.

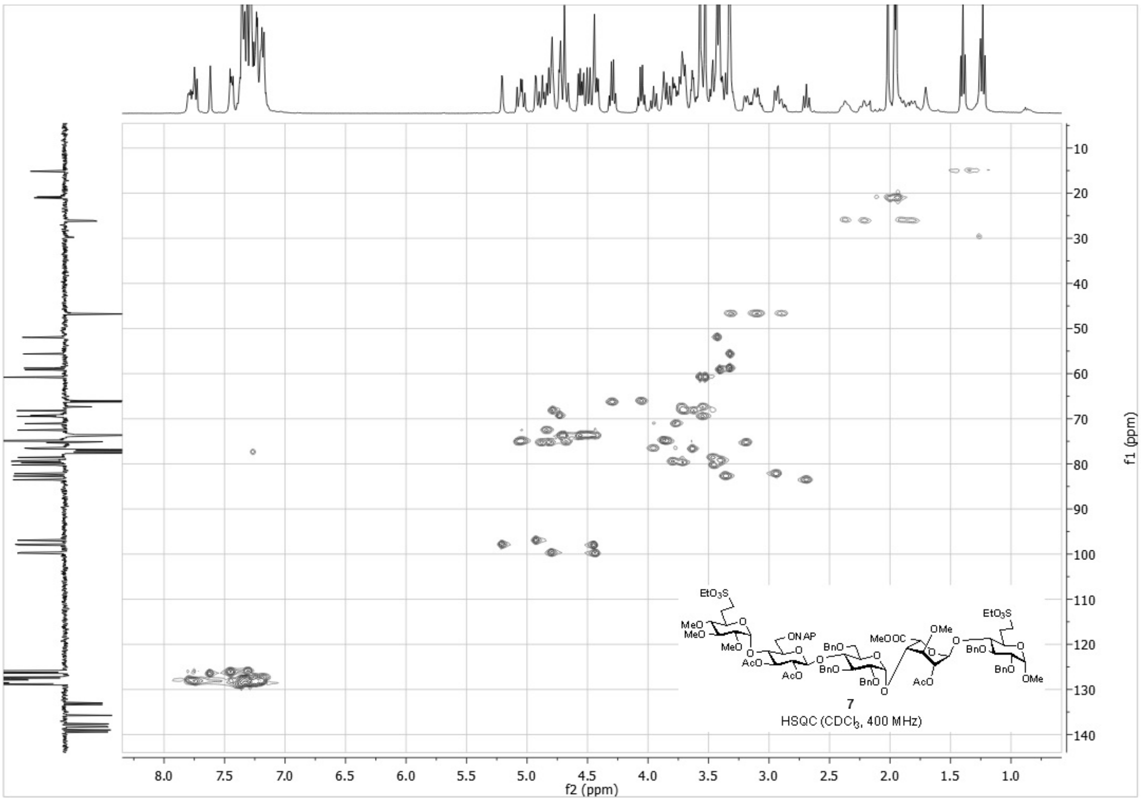

Figure S18. ^{1}H–^{13}C HSQC spectrum of compound **7** in $CDCl_3$.

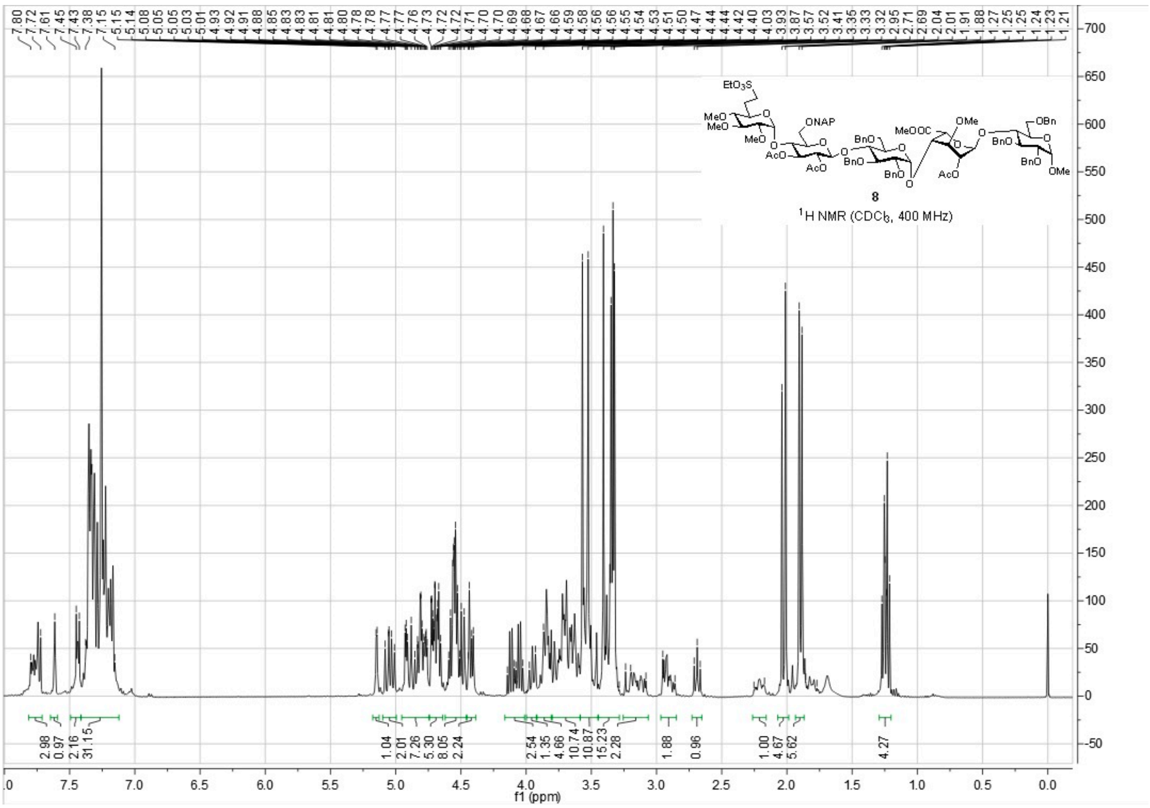

Figure S19. ^{1}H-NMR spectrum of compound **8** in $CDCl_3$.

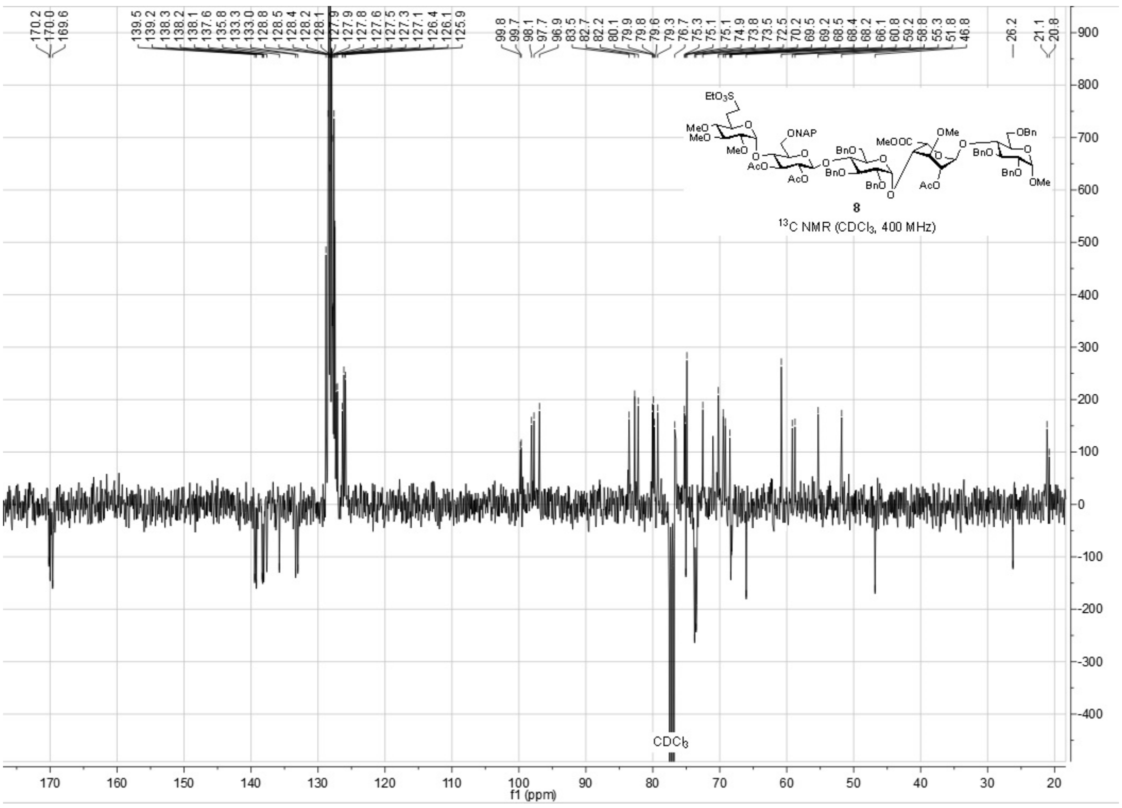

Figure S20. ^{13}C-NMR spectrum of compound **8** in $CDCl_3$.

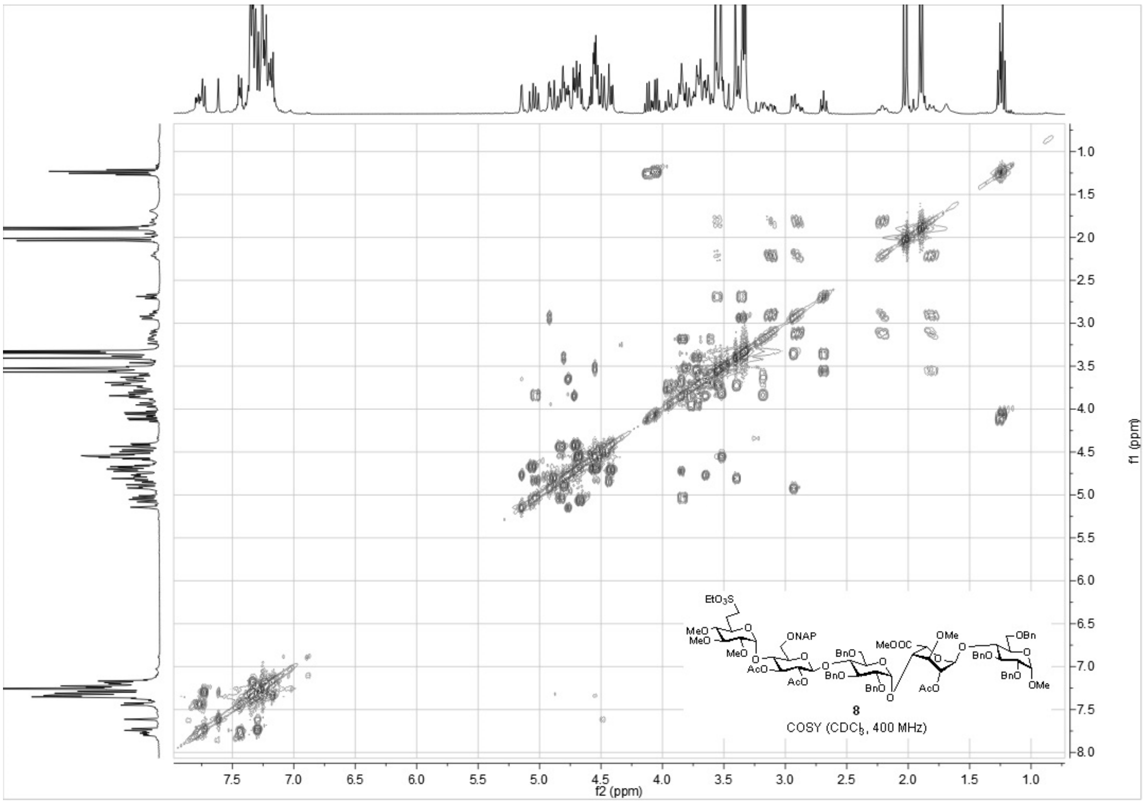

Figure S21. ^{1}H–^{1}H COSY spectrum of compound **8** in $CDCl_3$.

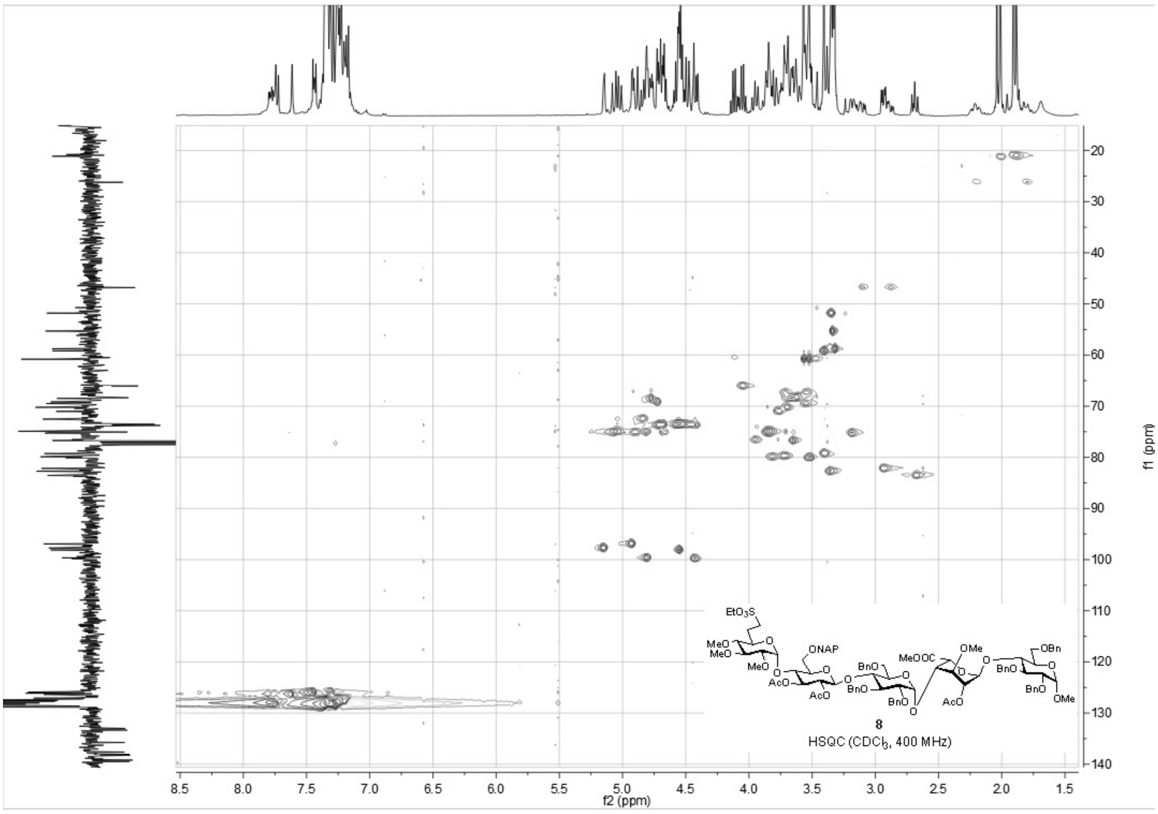

Figure S22. ^{1}H–^{13}C HSQC spectrum of compound **8** in $CDCl_3$.

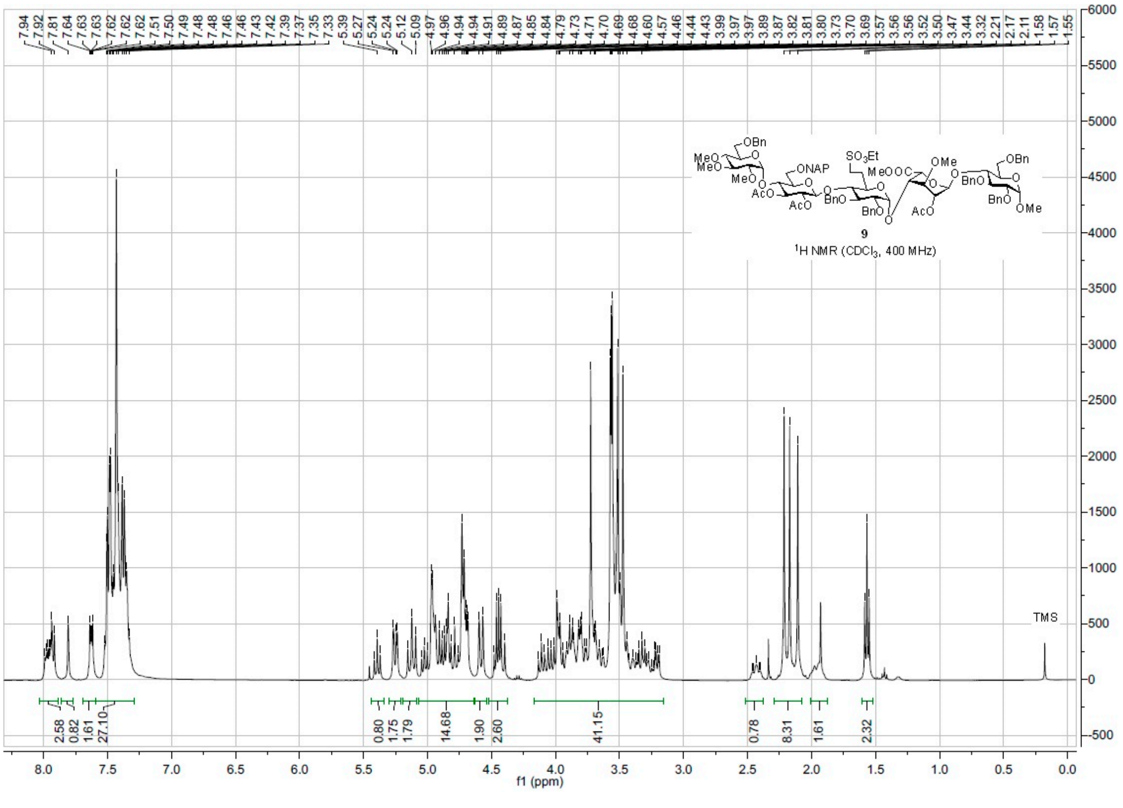

Figure S23. ^{1}H-NMR spectrum of compound **9** in $CDCl_3$.

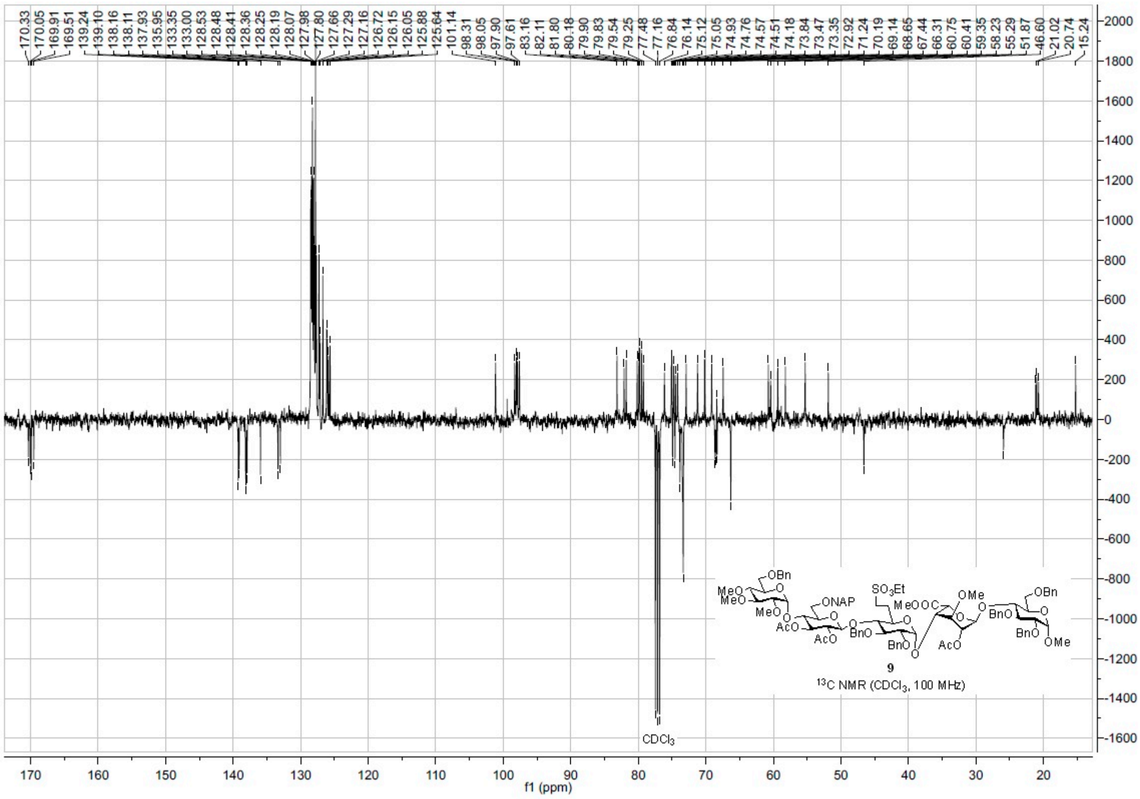

Figure S24. ^{13}C-NMR spectrum of compound **9** in $CDCl_3$.

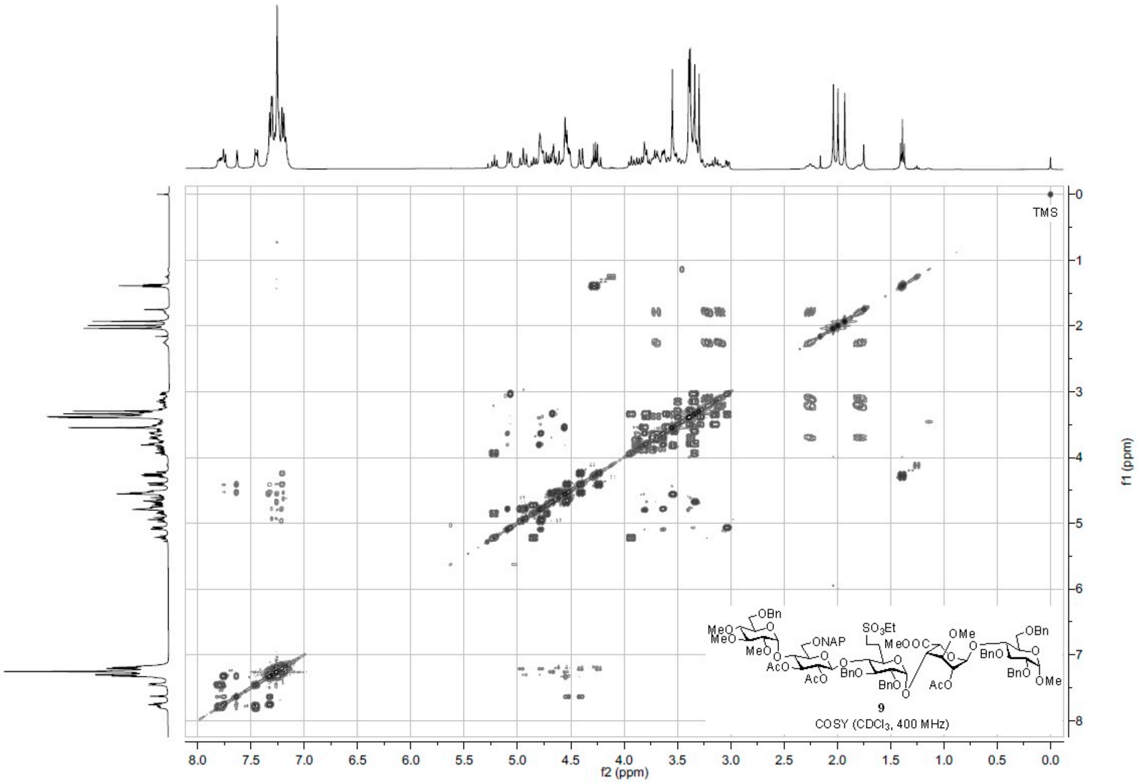

Figure S25. ^{1}H–^{1}H COSY spectrum of compound **9** in $CDCl_3$.

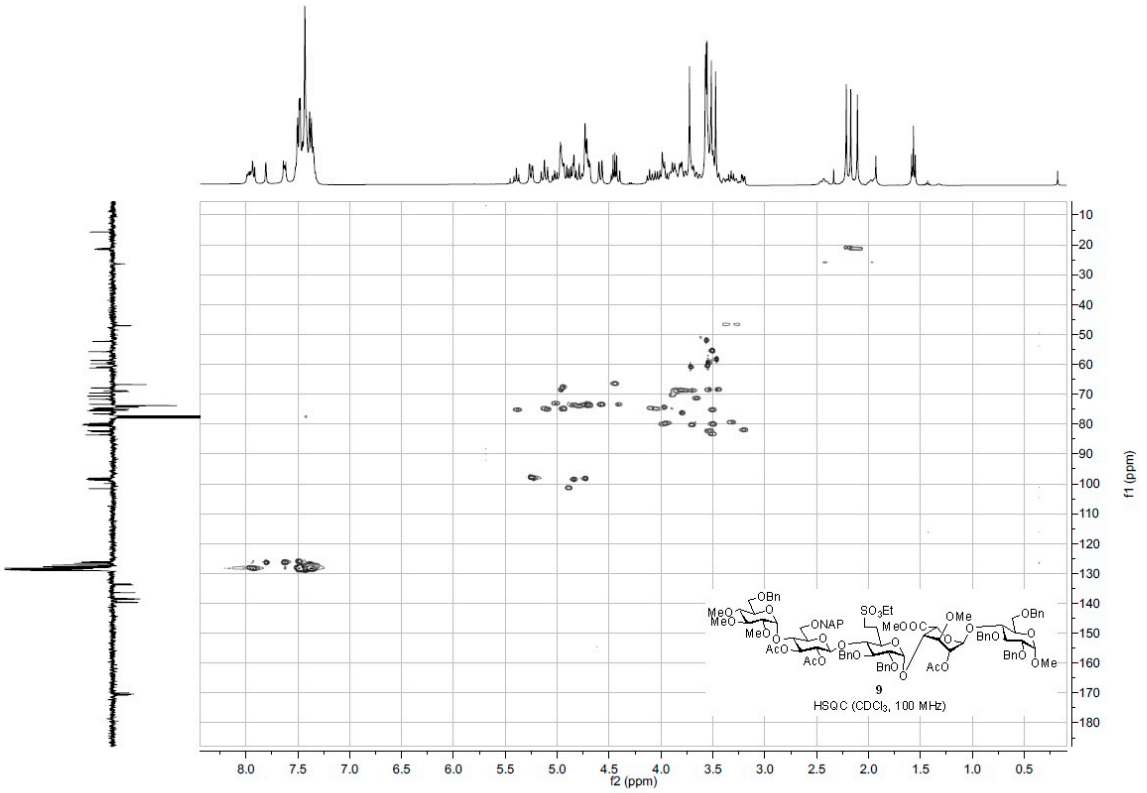

Figure S26. ^{1}H–^{13}C HSQC spectrum of compound **9** in CDCl3.

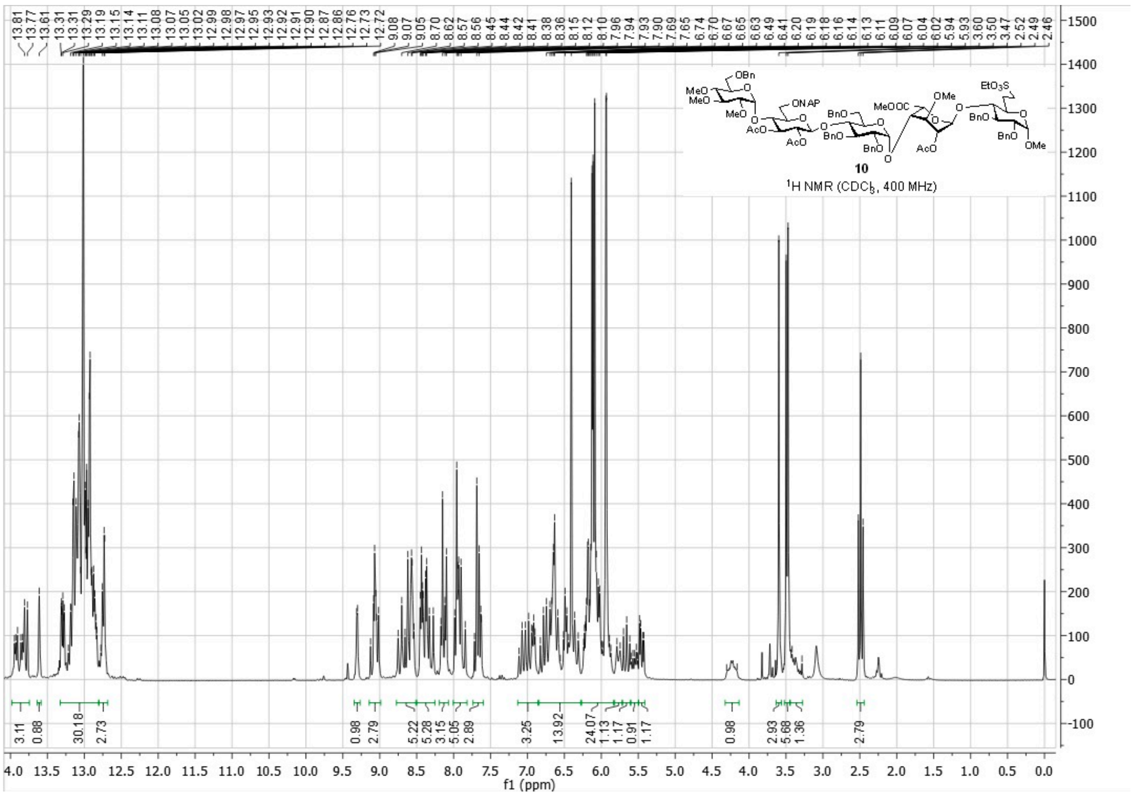

Figure S27. ^{1}H-NMR spectrum of compound **10** in CDCl3.

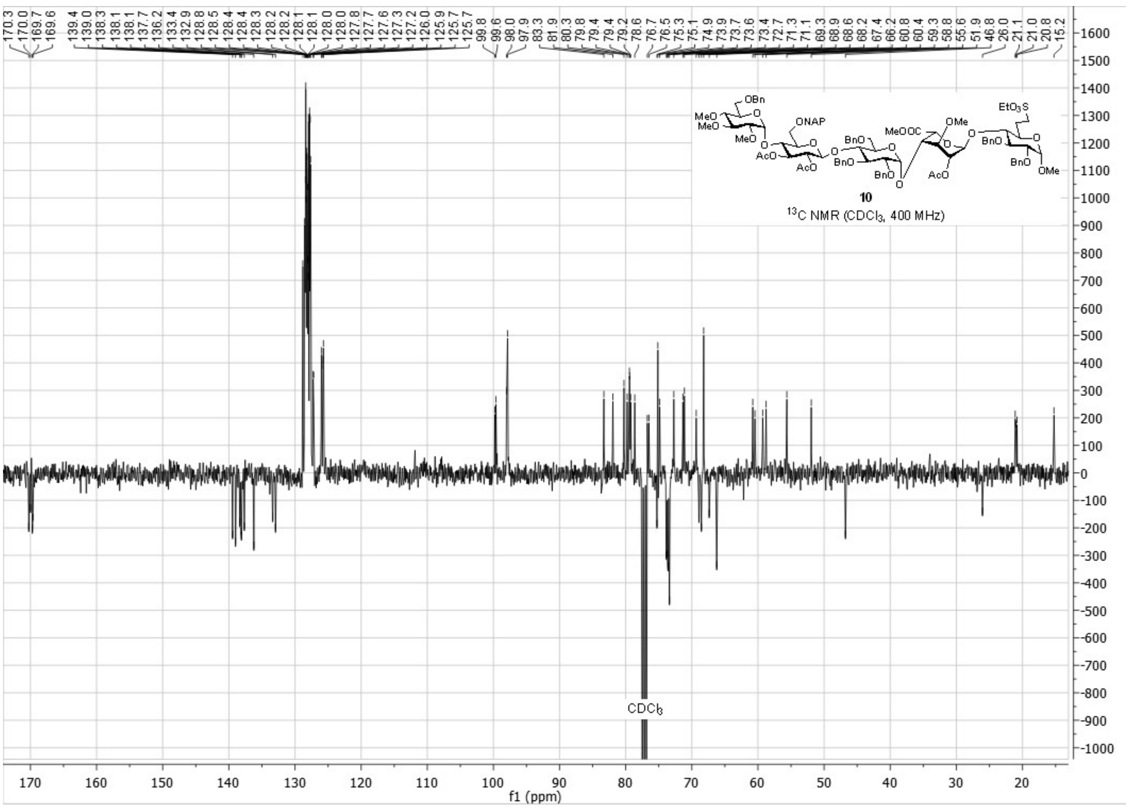

Figure S28. ^{13}C-NMR spectrum of compound **10** in CDCl$_3$.

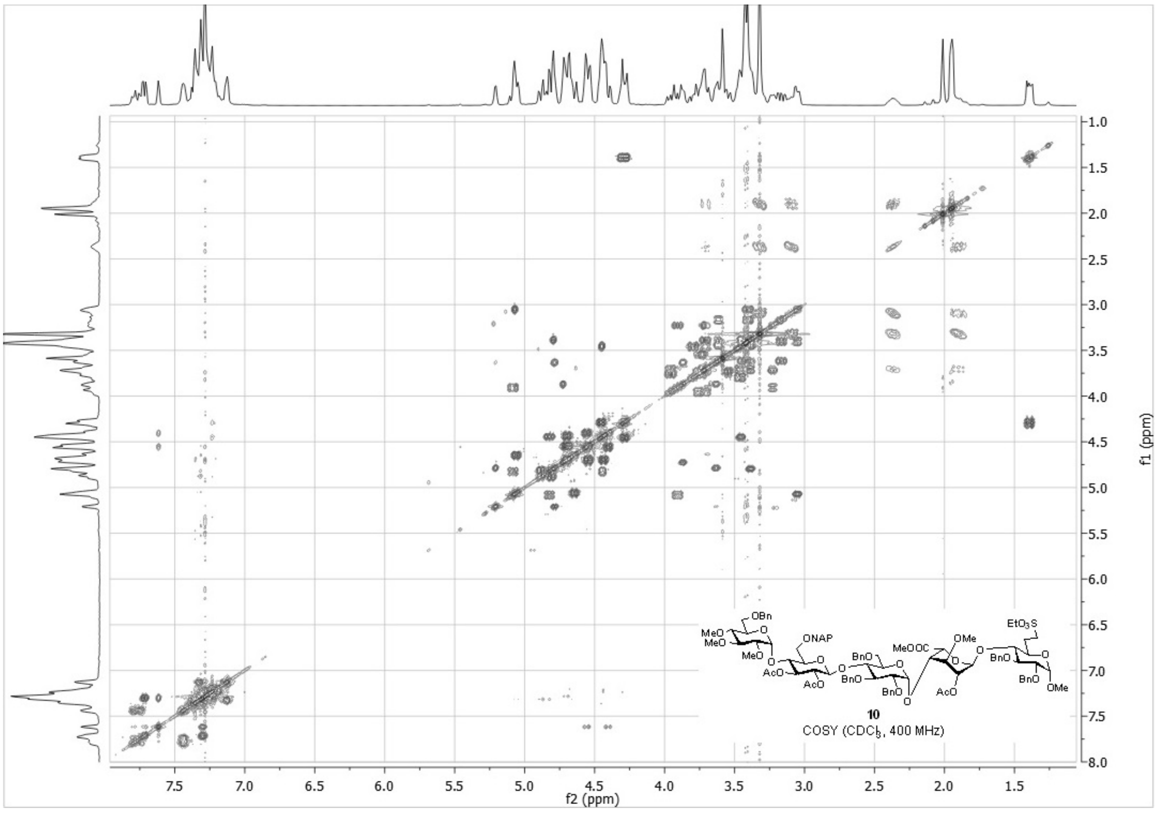

Figure S29. ^{1}H–^{1}H COSY spectrum of compound **10** in CDCl$_3$.

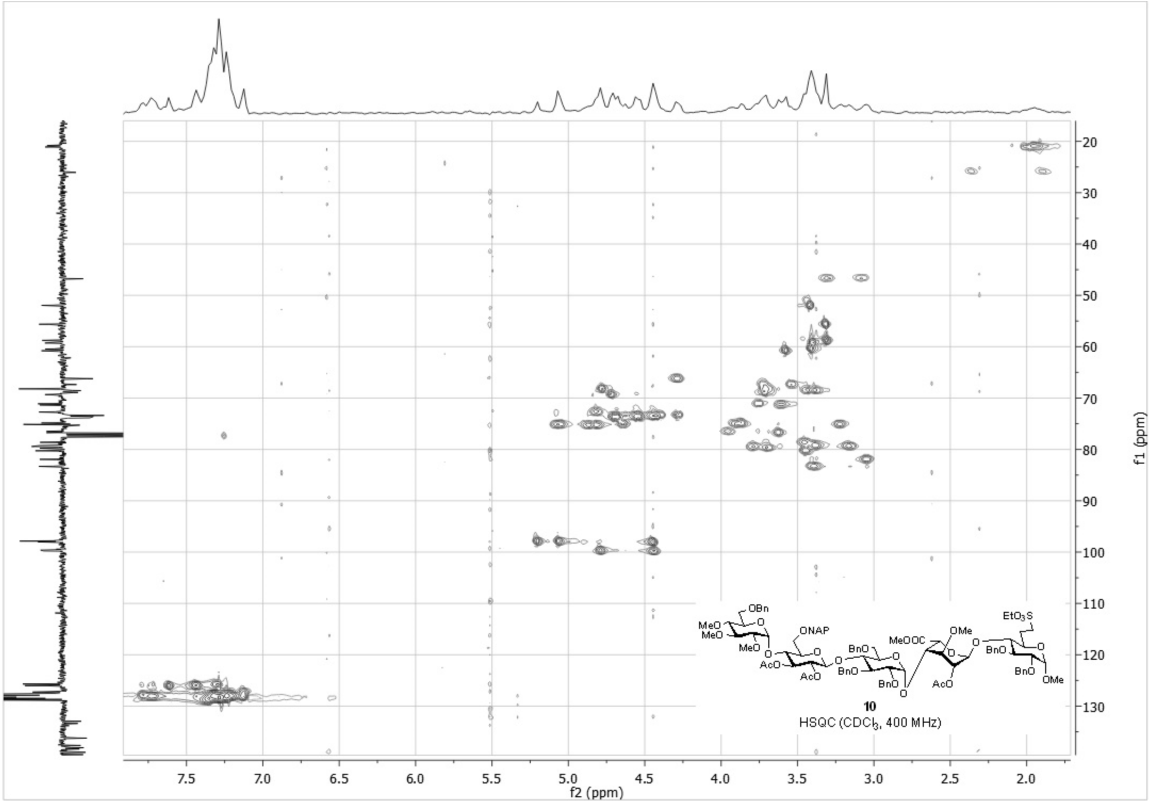

Figure S30. ^{1}H–^{13}C HSQC spectrum of compound **10** in $CDCl_3$.

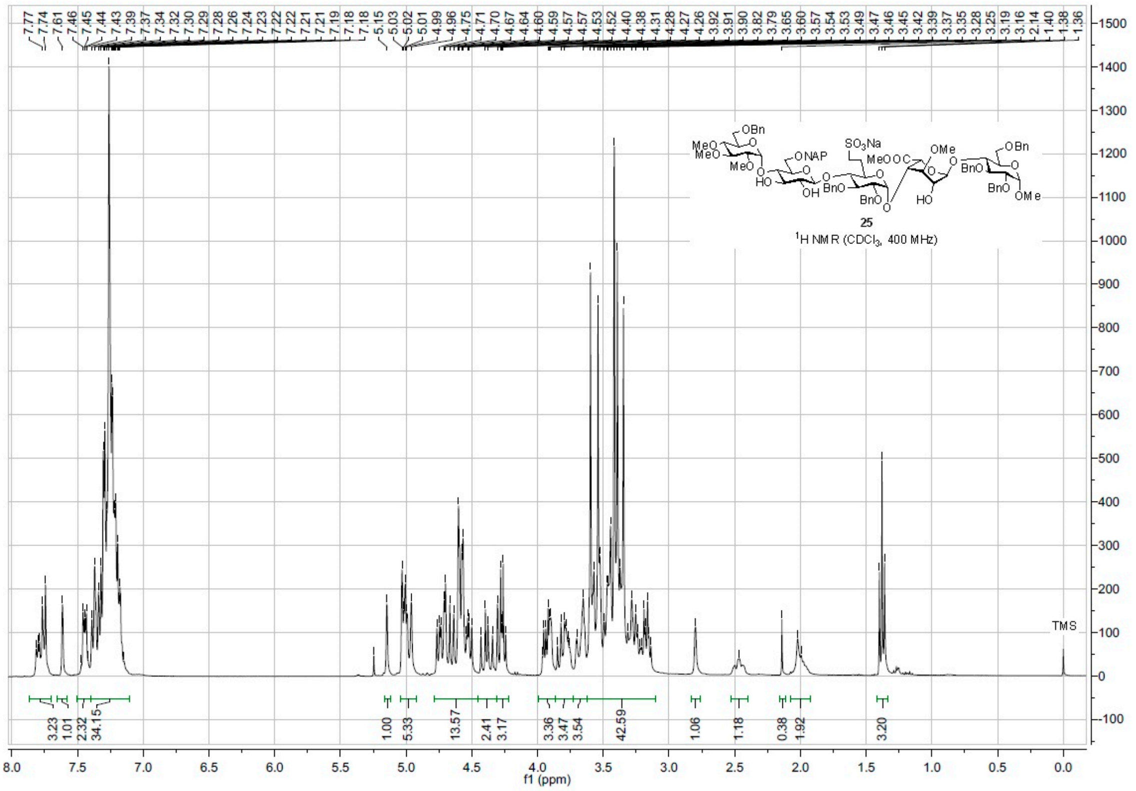

Figure S31. ^{1}H-NMR spectrum of compound **25** in $CDCl_3$.

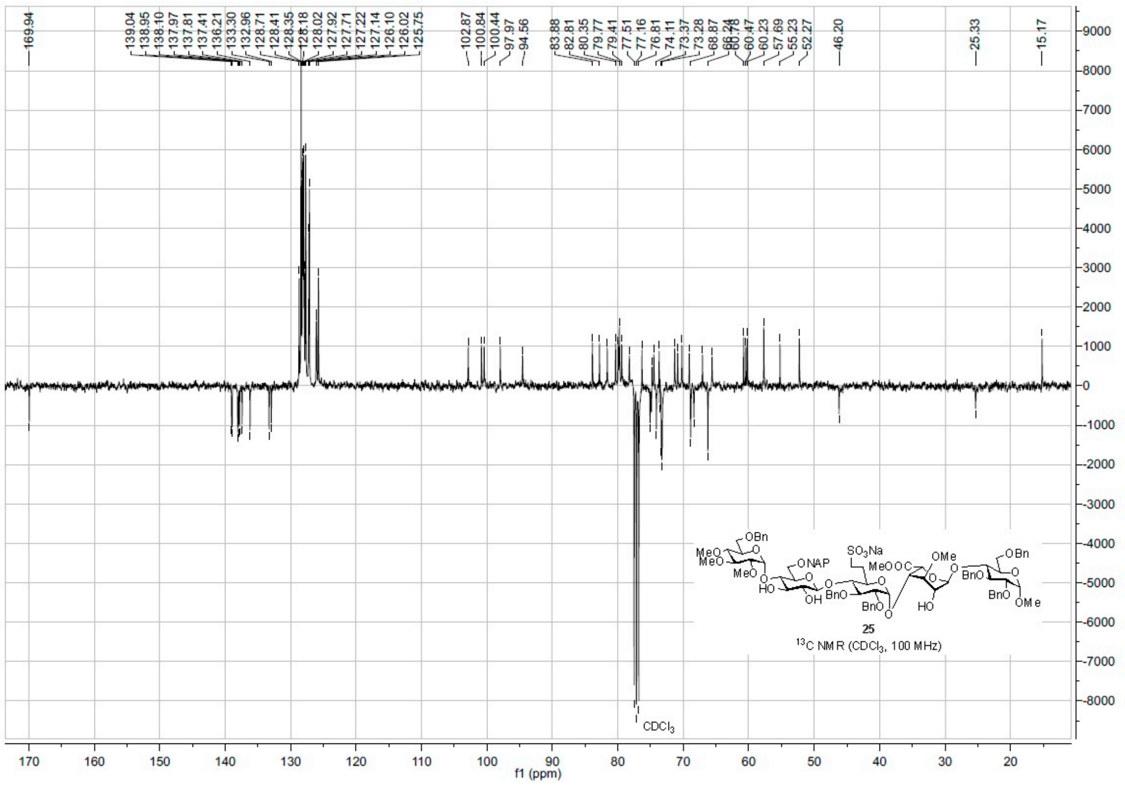

Figure S32. ^{13}C-NMR spectrum of compound **25** in $CDCl_3$.

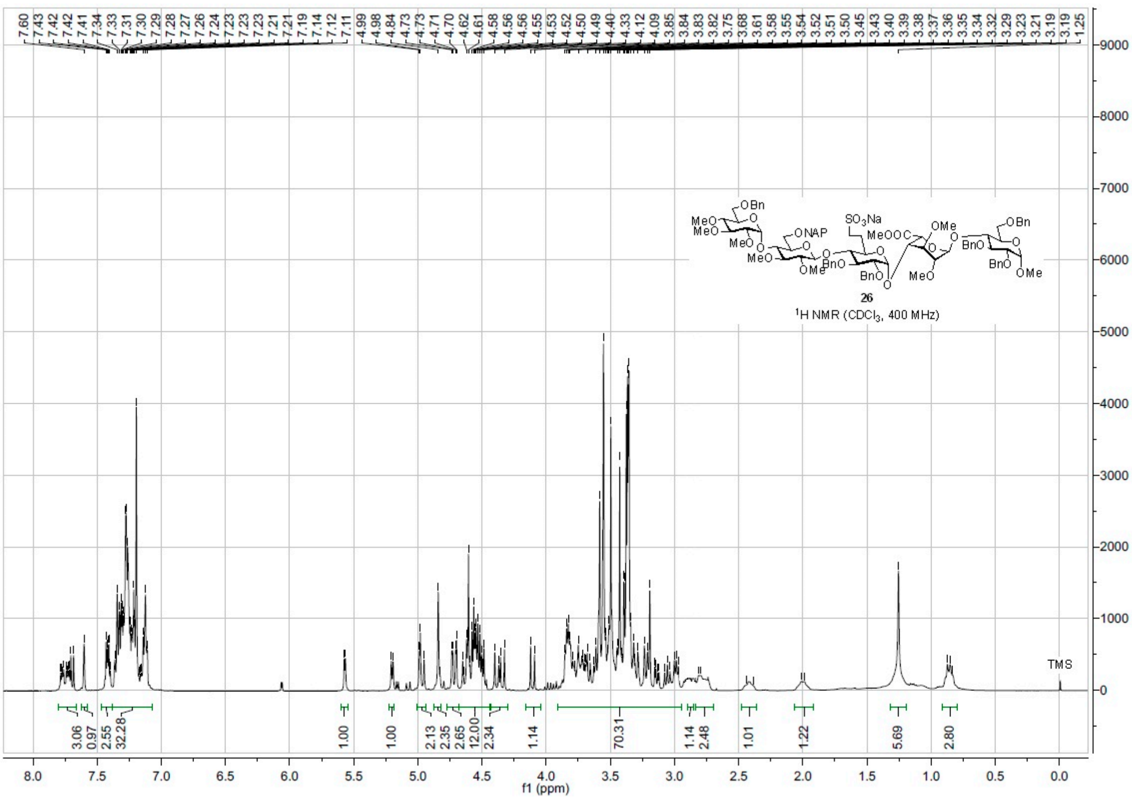

Figure S33. ^{1}H-NMR spectrum of compound **26** in a mixture of $CDCl_3$ and CD_3OD.

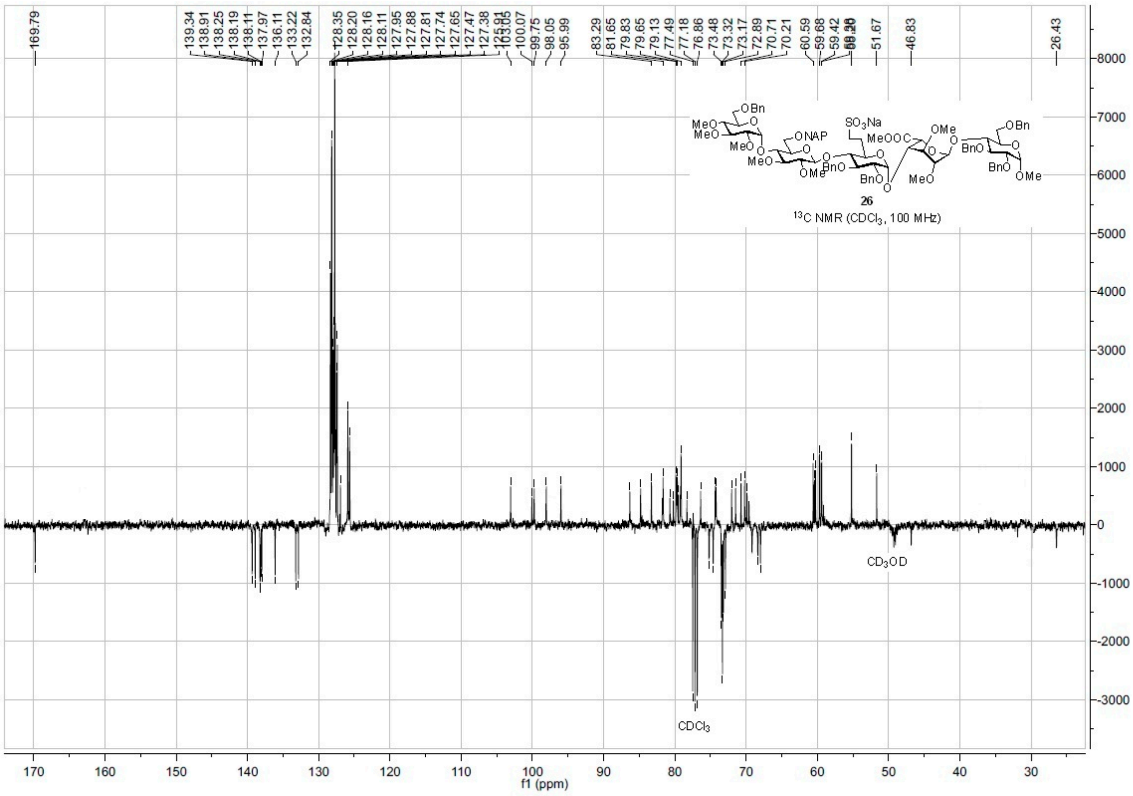

Figure S34. ^{13}C-NMR spectrum of compound **26** in a mixture of $CDCl_3$ and CD_3OD.

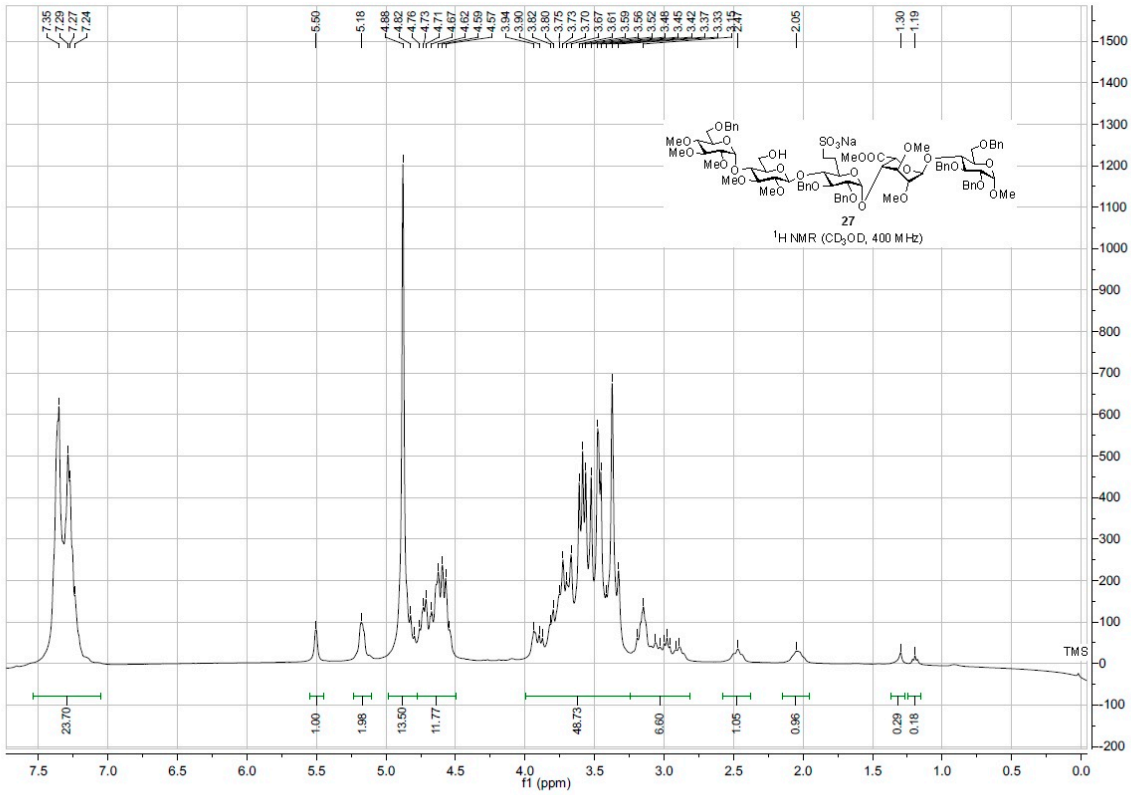

Figure S35. ^{1}H-NMR spectrum of compound **27** in CD_3OD.

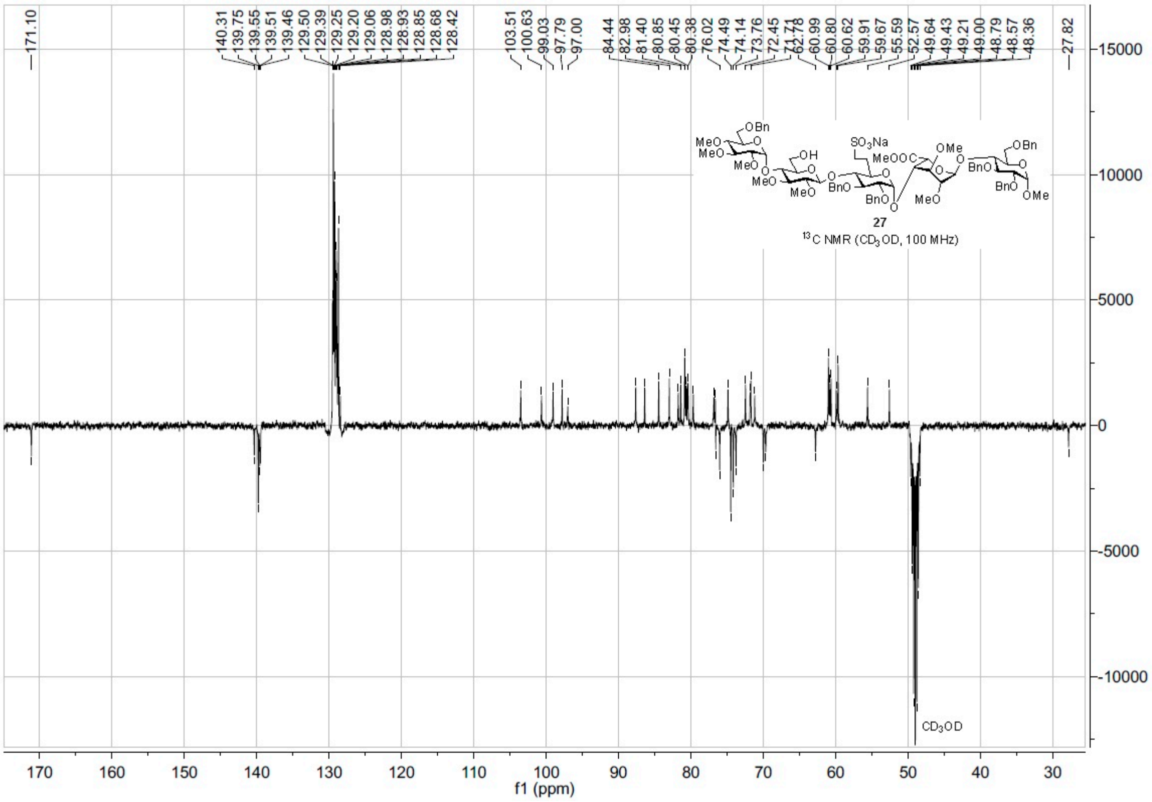

Figure S36. ^{13}C-NMR spectrum of compound **27** in CD$_3$OD.

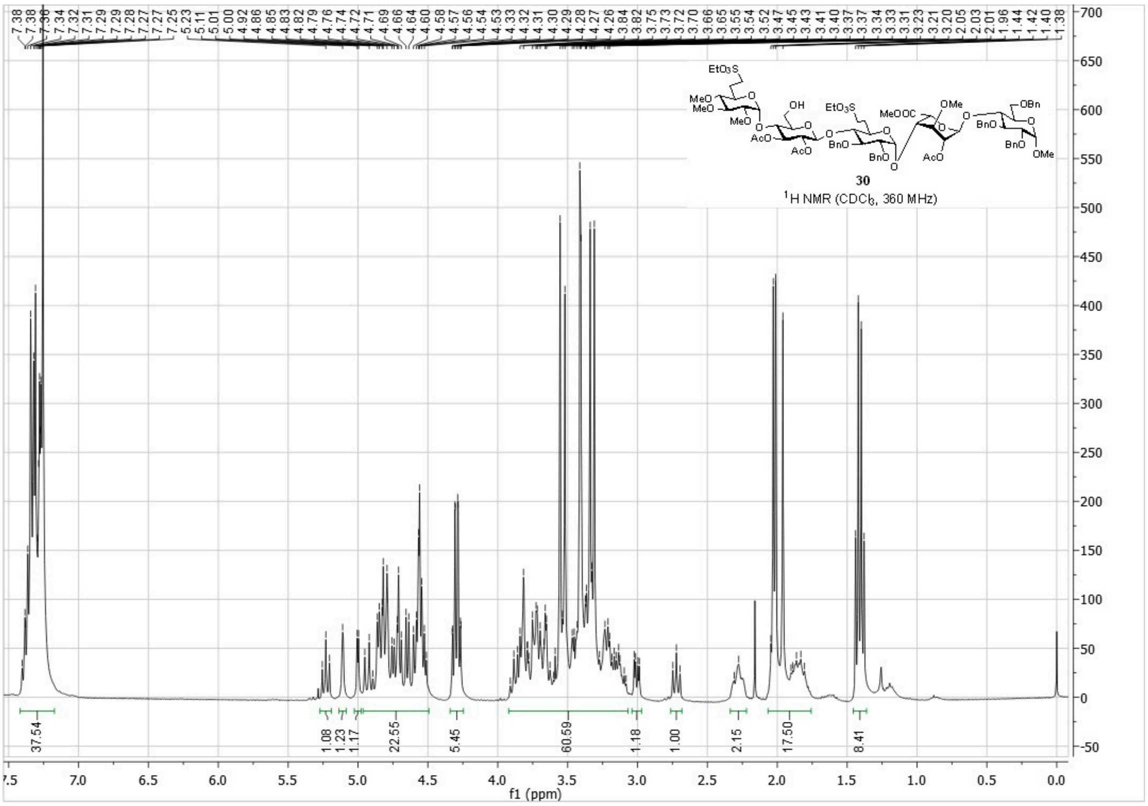

Figure S37. ^{1}H-NMR spectrum of compound **30** in CDCl$_3$.

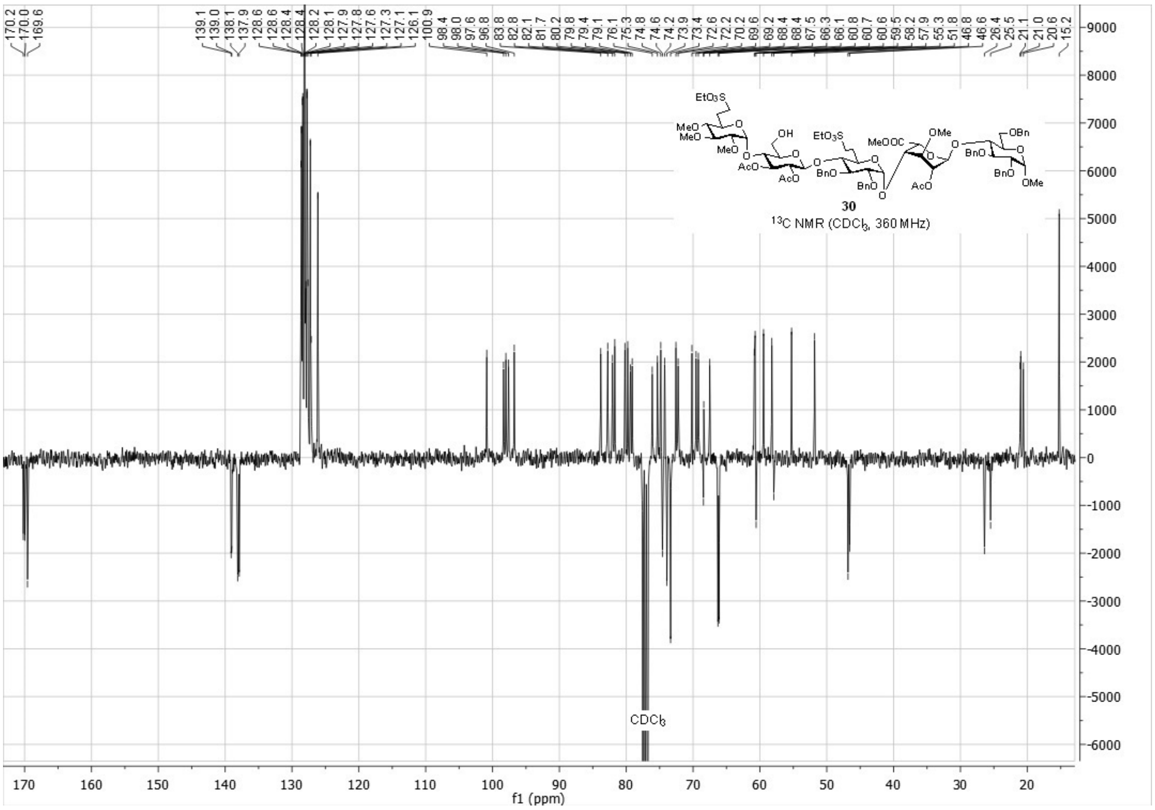

Figure S38. ^{13}C-NMR spectrum of compound **30** in $CDCl_3$.

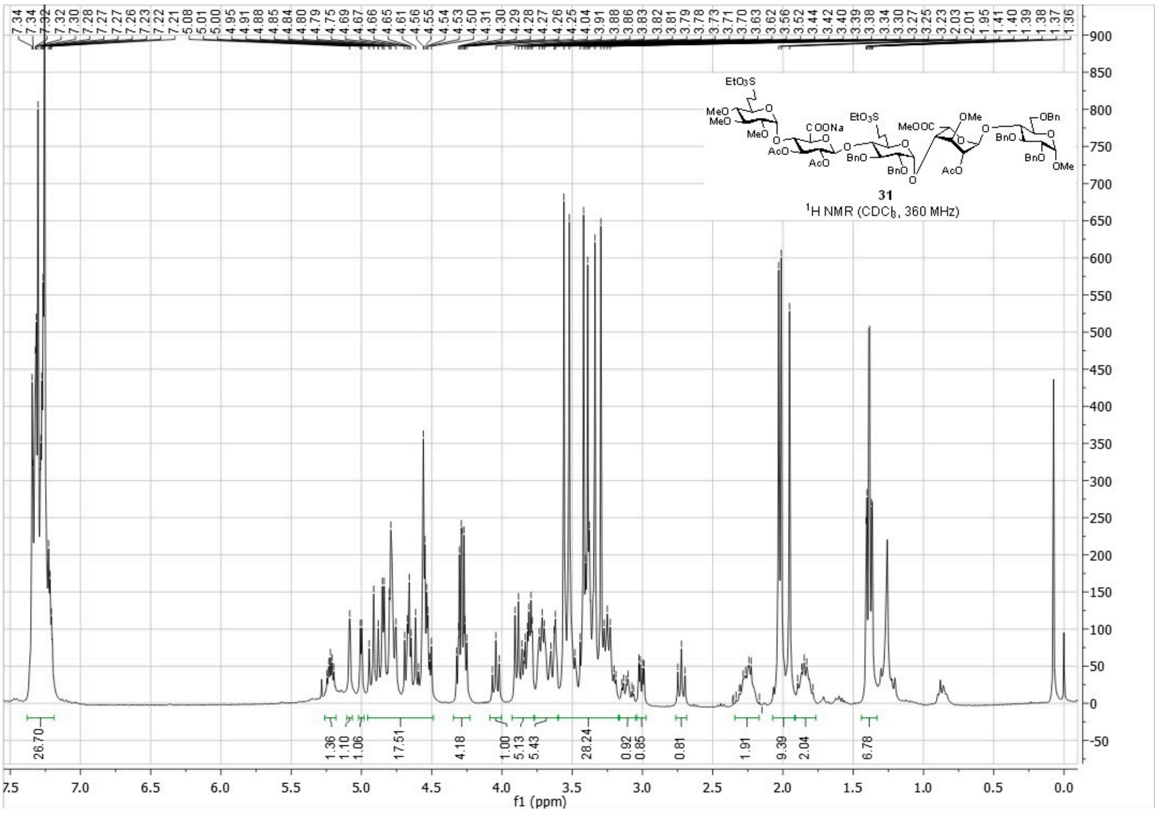

Figure S39. ^{1}H-NMR spectrum of compound **31** in $CDCl_3$.

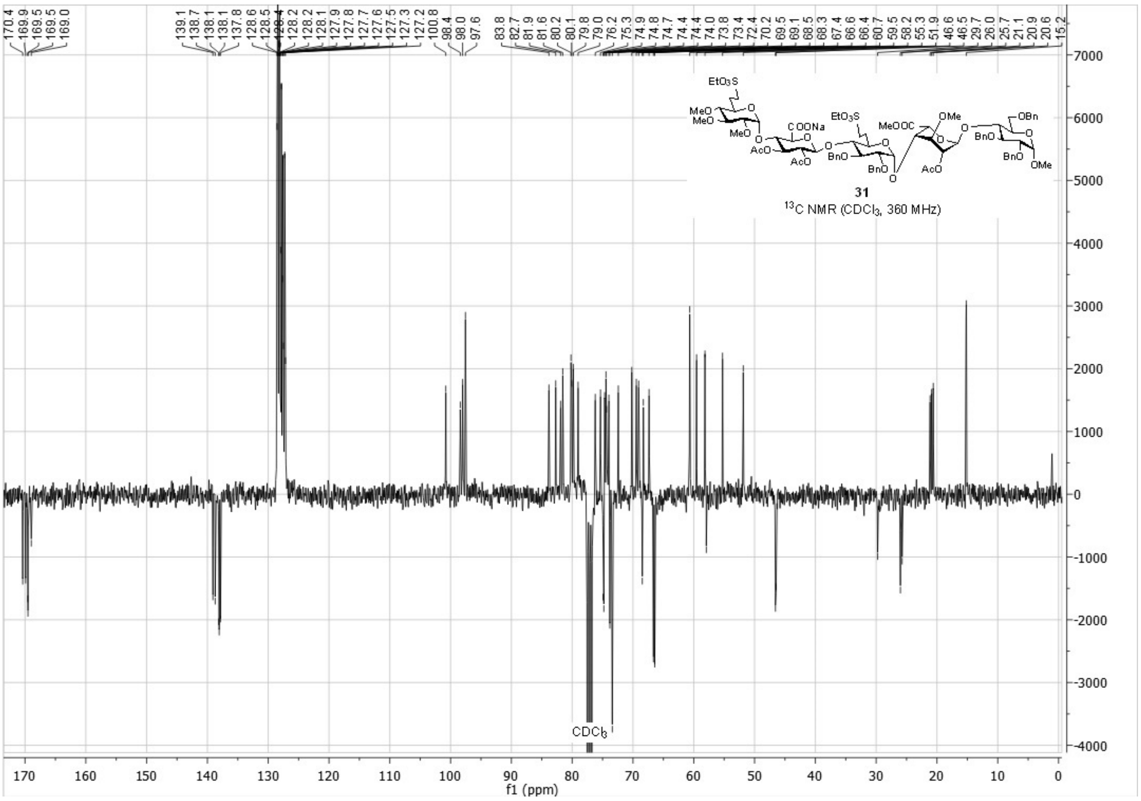

Figure S40. ^{13}C-NMR spectrum of compound **31** in $CDCl_3$.

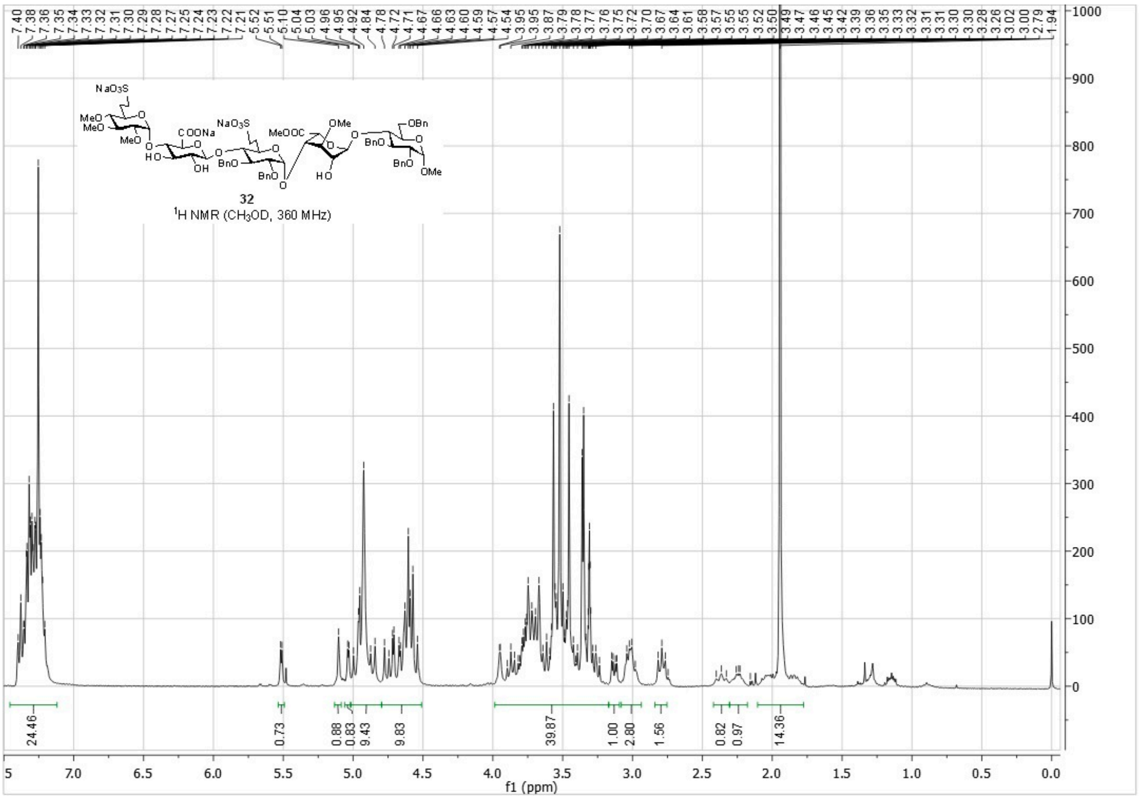

Figure S41. ^{1}H-NMR spectrum of compound **32** in CD_3OD.

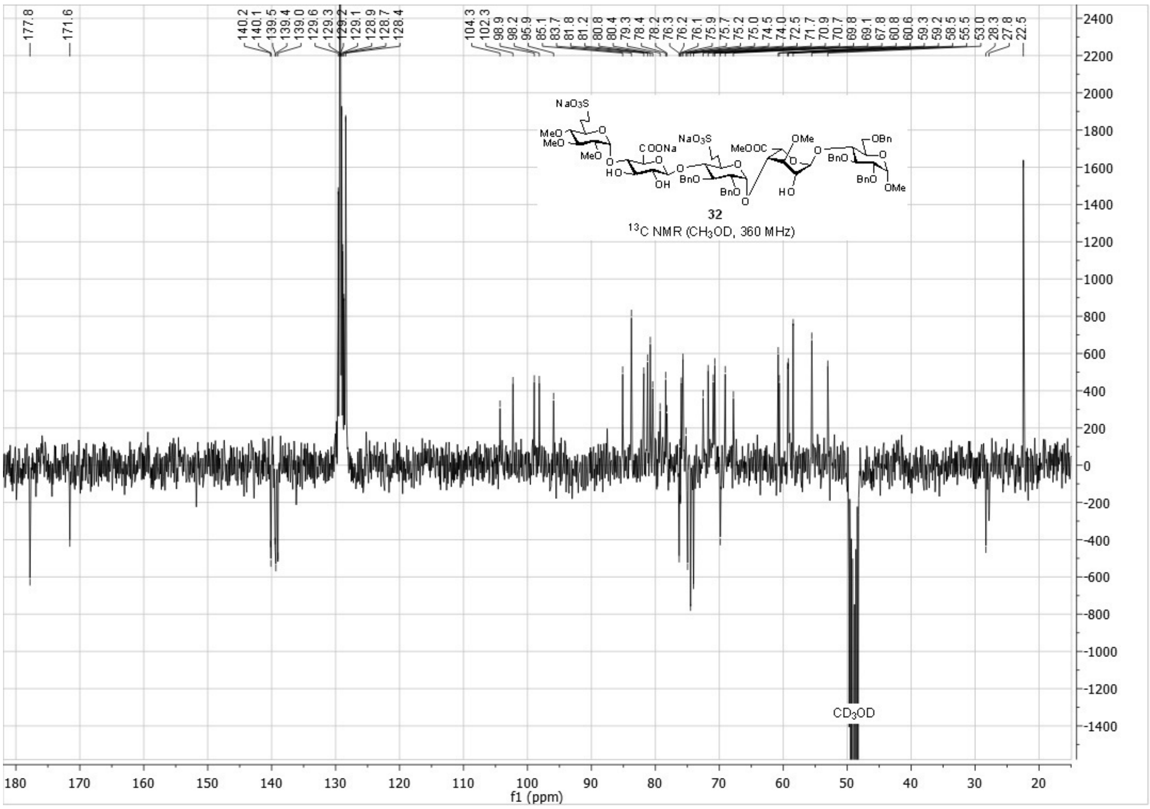

Figure S42. ^{13}C-NMR spectrum of compound **32** in CD_3OD.

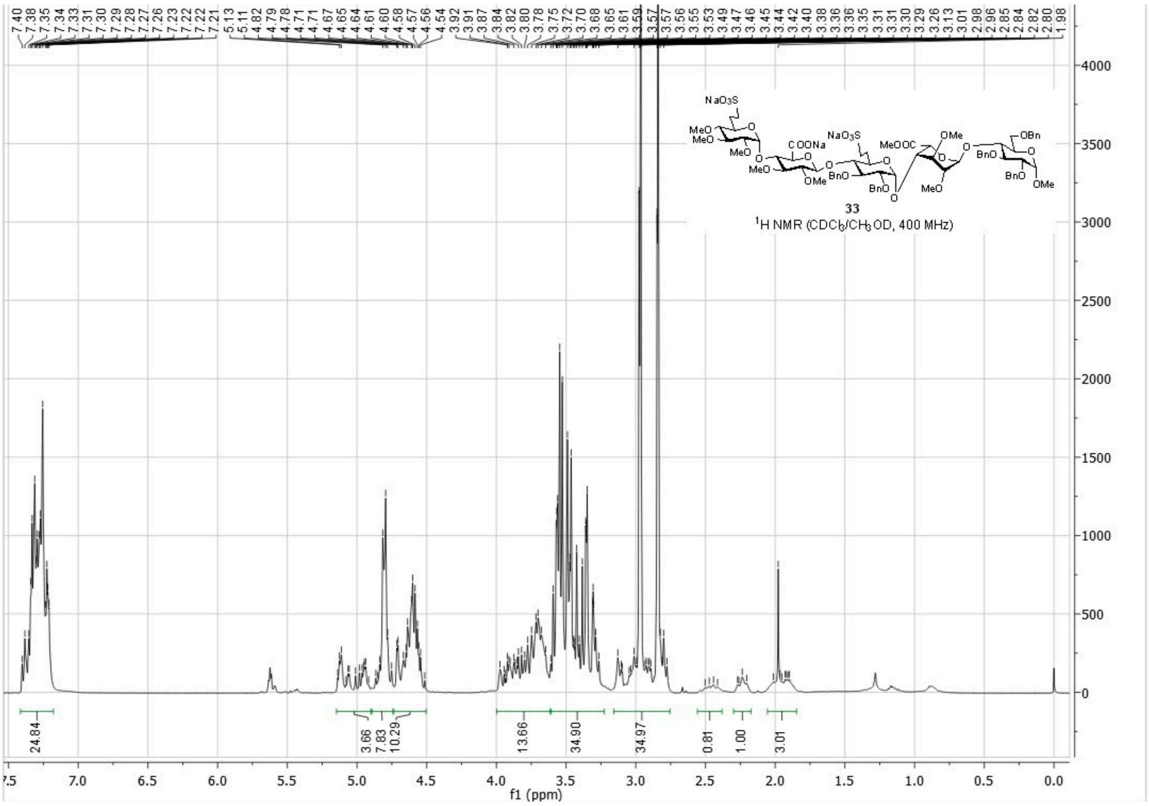

Figure S43. ^{1}H-NMR spectrum of compound **33** in a mixture of $CDCl_3$ and CD_3OD.

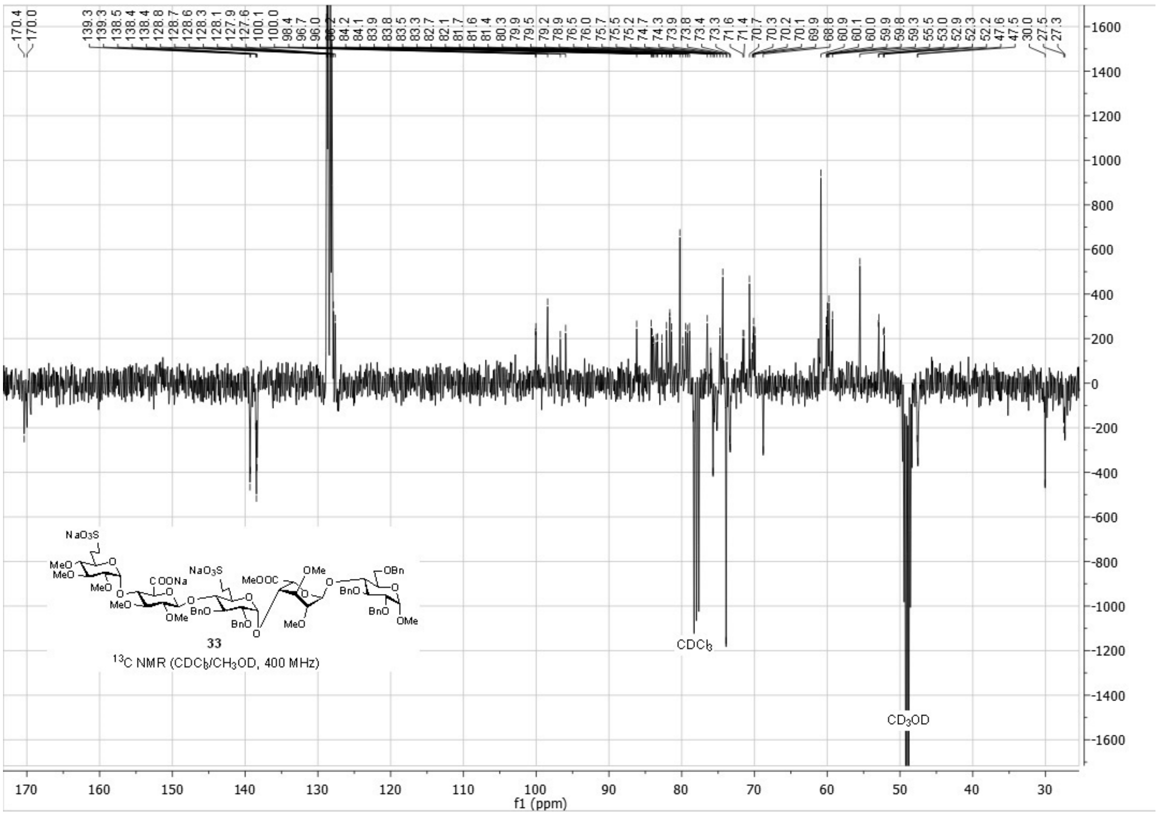

Figure S44. ^{13}C-NMR spectrum of compound **33** in a mixture of $CDCl_3$ and CD_3OD.

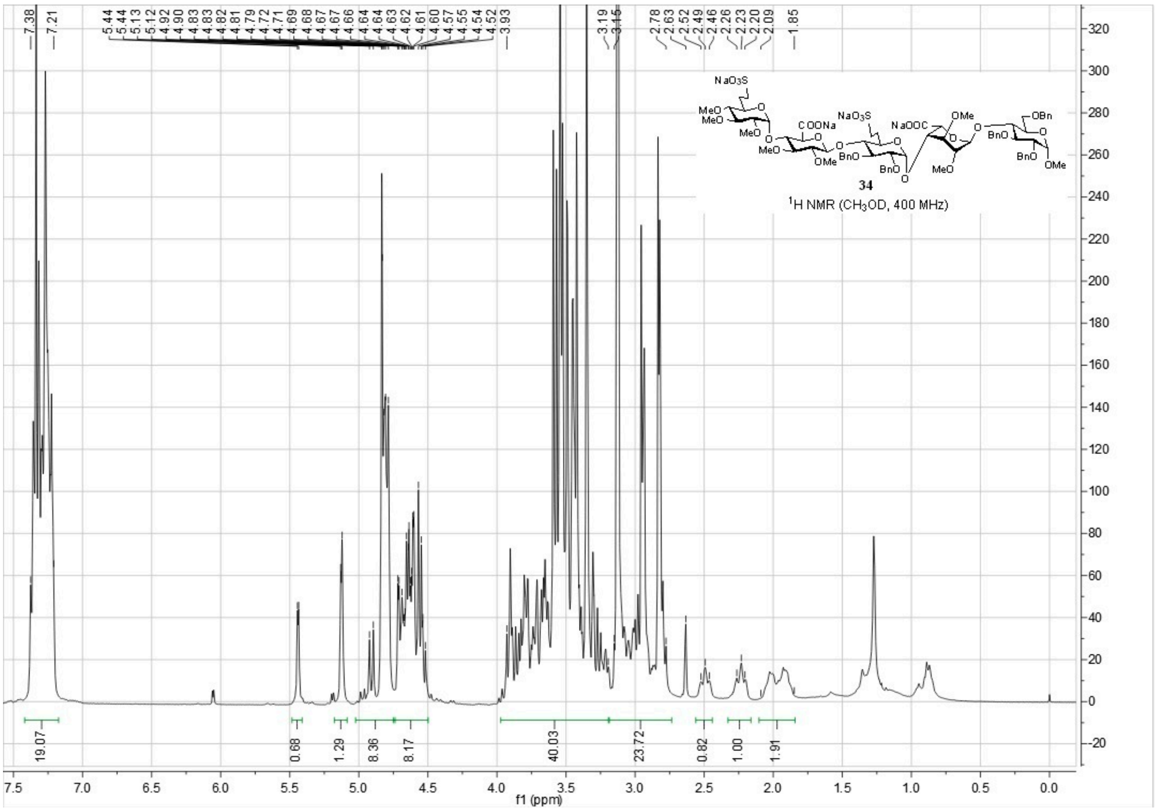

Figure S45. ^{1}H-NMR spectrum of compound **34** in CD_3OD.

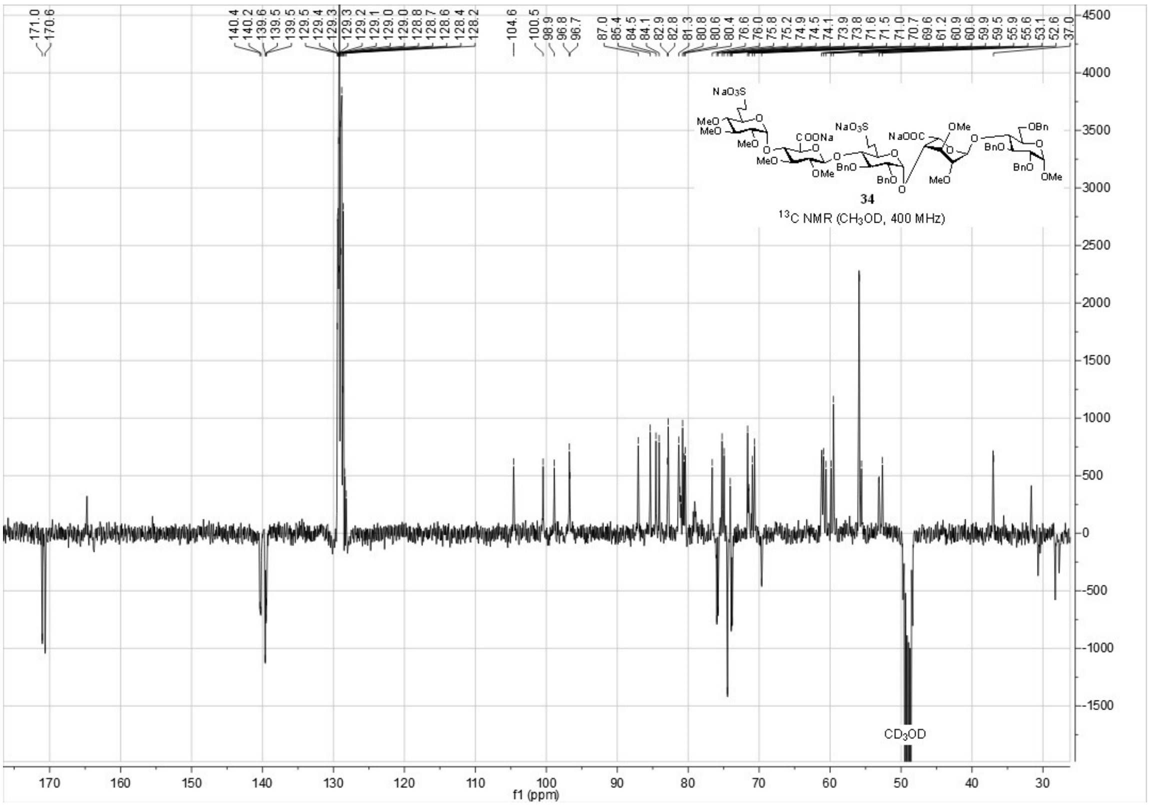

Figure S46. ^{13}C-NMR spectrum of compound **34** in CD_3OD.

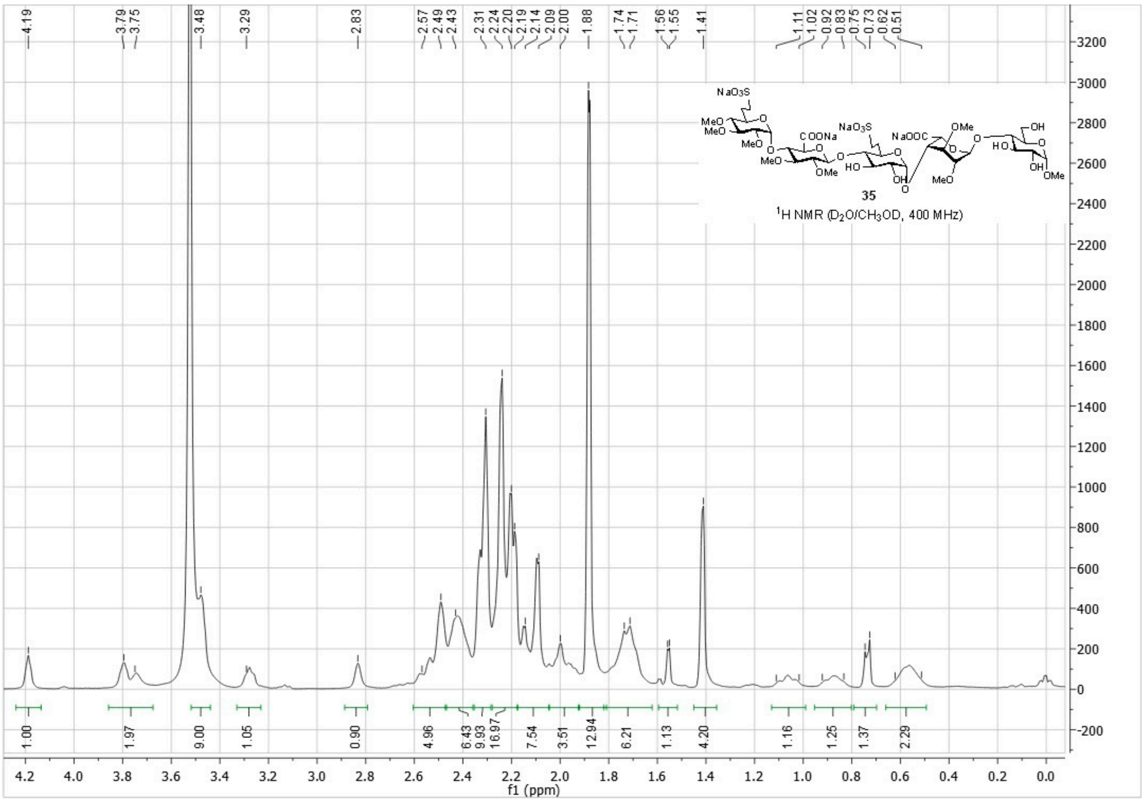

Figure S47. ^{1}H-NMR spectrum of compound **35** in a mixture of D_2O and CD_3OD.

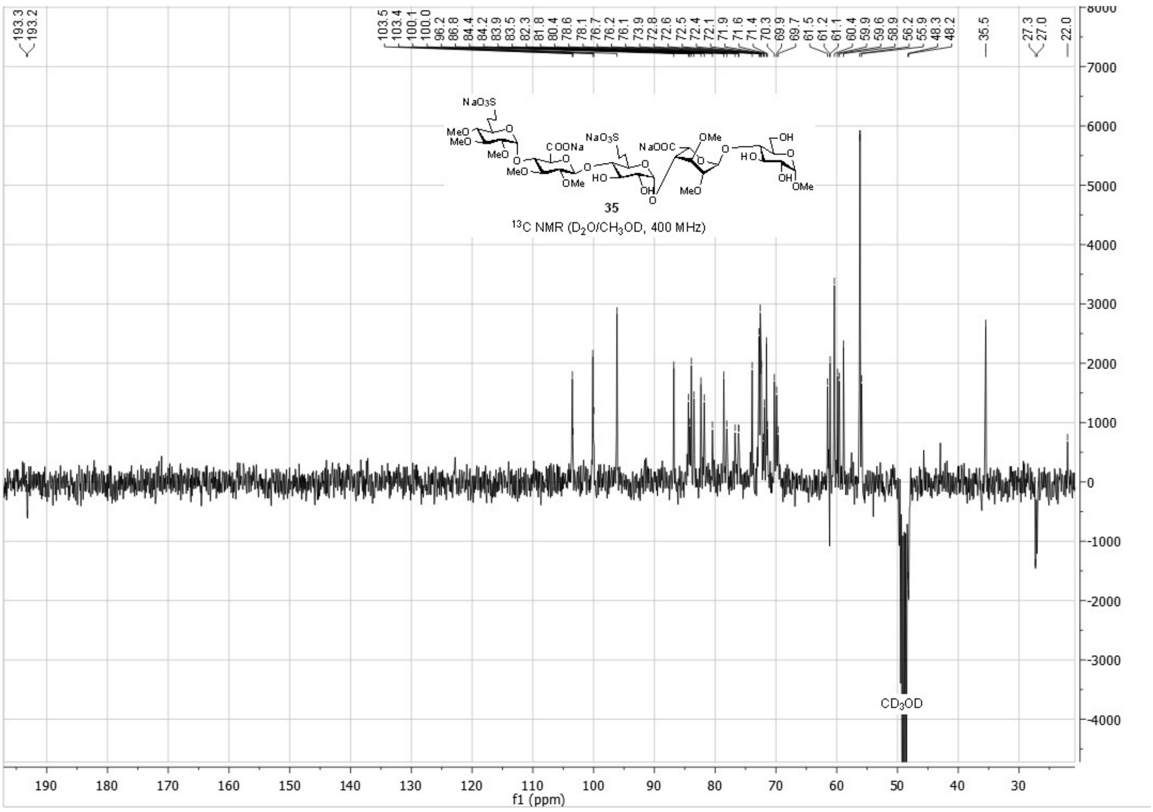

Figure S48. ^{13}C-NMR spectrum of compound **35** in a mixture of D_2O and CD_3OD.
